# Bridging material culture networks and social networks in archaeology

**DOI:** 10.1371/journal.pone.0358860

**Published:** 2026-10-16

**Authors:** Robert J. Bischoff, Cecilia Padilla-Iglesias

**Affiliations:** 1 School of Human Evolution and Social Change, Arizona State University, Tempe, Arizona, United States of America; 2 HUMAN ORIGINS - Cluster of Excellence for Integrative Human Origins Studies (EXC 3101), University of Tübingen, Tübingen, Germany; The University of Tulsa, UNITED STATES OF AMERICA

## Abstract

This study examines whether material culture can serve as a proxy for past social interaction. Using the ArchMatNet agent-based model, we simulate social behavior in small-scale communities and resulting material culture production, then compare social and material culture networks. We compare networks using graph correlation and the quadratic assignment procedure, evaluate parameter effects with generalized additive models, and assess underlying structures with exponential random graph models. Within this simulated system, results show strong correlations between social interaction networks and material culture networks, especially when material culture traits are sufficiently variable. However, correlations weaken under some conditions, particularly low material culture variation, indicating limits to archaeological network inference. Key factors include interaction frequency, learning strategy, and trait visibility. These results characterize hypothetical relationships generated under the model’s assumptions; they do not constitute empirical validation. They highlight both the promise and the constraints of using material culture as a proxy for social networks and underscore the need for empirical testing and careful attention to network structure.

## Introduction

Over the last two decades, network studies have proliferated in archaeology and the social and behavioral sciences. In archaeology, much of this work has focused on building empirical networks from artifact distributions and frequencies. A core assumption is that similarities in artifact categories or types can serve as proxies for connections among people at different scales [see 1]. Although researchers often acknowledge the complexity of relationships between material similarity and social relationships [2–4], relatively little work has explicitly tested the assumptions underlying these network models [but see 5,6]. This raises a key question: to what extent can similarities in artifact attributes represent past interaction patterns, and how do these patterns relate to formal network models (Fig 1), we address this question using agent-based models and statistical methods for network comparison.

**Fig 1:**
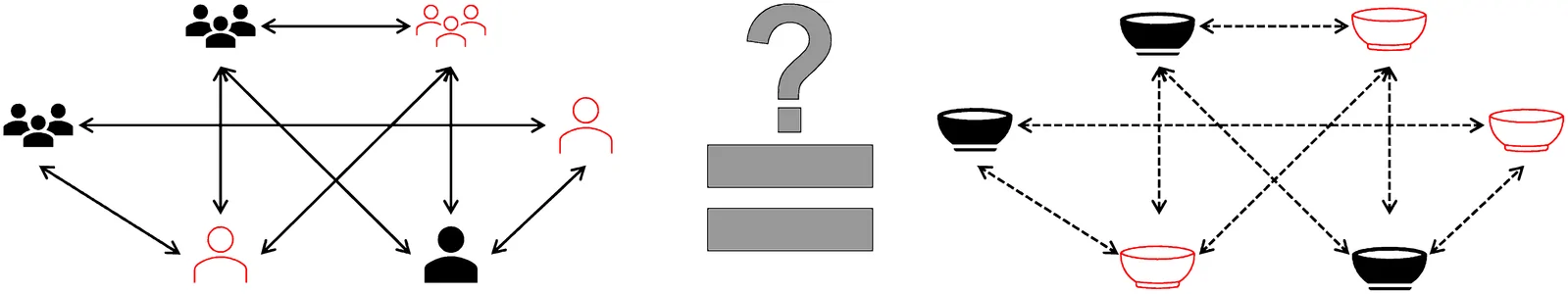
An abstract comparison of a social network and a material culture network, representing the core research question: to what extent do patterns in material culture reflect underlying social interactions?.

### Material culture as a social proxy

Discussions about material culture in archaeology, particularly its use as a proxy for social interaction, are extensive [7–11]. Although the arguments about the connections between material patterns and social processes are diverse, there are a few commonalities that have been recognized across a broad array of contexts. For example, highly visible aspects of style (e.g., painted designs, public buildings, ceramic decoration, textile patterns, personal adornments) are widely seen and displayed. Thus, material similarities may indicate some degree of interaction between producers or simply imitation without direct contact. On the other hand, similarities in low-visibility technological aspects of design and production (e.g., hidden foundation stones) tend to result from direct, frequent interactions, such as those found in shared learning environments.

This essentially mirrors older debates in archaeology and the anthropology of technology focused on style vs. isochrestic variation and relations with other social processes [12–14, see 8]. Numerous studies have examined the connection between material culture and social identity [7,9,11,15–22], revealing that variation in technological practices–whether in building, pottery, or weapons–can sometimes reflect social identity, though this is not always the case. Determining the significance of such variation requires empirical analysis on a case-by-case basis. We define social identity in a broad sense recognizing that it is mutable and multiple for each person.

There are some recurring patterns in the literature on material culture and identity, such as the role visibility plays in linking material style to social behavior [16,23]. Visibility influences transmission levels: low-visibility traits are harder to observe and replicate, typically requiring more skill and closer involvement, while high-visibility traits are often more easily copied. For instance, the spread of low-visibility technologies is often interpreted as evidence of migration, while the spread of highly visible styles is more likely to signify emulation or diffusion [17]. This is because high-visibility traits are more likely to be copied than low-visibility traits. Communities of practice tend to exhibit commonalities in low-visibility attributes, reflecting the strong relational connections within them. By contrast, constellations of practice (groups of related communities) show greater diversity yet still share relational connections through history, kinship, geography, or other ties [24,25]. Sharing high-visibility attributes suggests a social connection but does not necessitate frequent interaction.

Two primary strategies through which high- or low-visibility traits may be learned are prestige and conformism. Prestige-based transmission occurs when individuals preferentially imitate those perceived as successful or esteemed within their group, thereby acquiring skills or knowledge associated with status. These traits are presumed to be more advantageous [26,27]. In contrast, conformist transmission refers to the tendency of individuals to adopt the most common behaviors or beliefs within their social group, leading to homogenization of cultural traits. This mechanism can enhance group cohesion and maintain cultural boundaries because it tends to reduce within-group variation while increasing between-group differences [26,28]. However, while conformist transmission can stabilize cultural norms, it may also impede the spread of innovation, especially in dynamic environments where adaptability is crucial [27]. Both transmission types occur in any given society, although conformism is often dominant [29].

As the literature suggests, the relationship between material culture and social interaction is complex. It becomes clear that there is no universal answer to how effectively any form of material culture can act as a proxy for social interaction. Instead, we must approach the question from a broader perspective while accounting for historical contingencies that shape interpretations of specific types of material culture at different times.

### The role of simulation in archaeological validation

Leaving aside the question of how specific types of material culture relate to social identity or relationships, how can the relationship between social interaction networks and material culture be evaluated if archaeologists cannot observe the past? How can we validate our methods and assumptions? Moreover, how do various social processes like exchange, migration, intermarriage, and cultural diffusion affect these patterns [see 30 for additional discussion on these questions]?

One avenue to address these questions is through ethnoarchaeological studies of material culture. General models linking material culture and identity are often informed by such case studies [17]. In our review of candidate datasets, however, we identified four major limitations: (1) data availability, (2) study scale, (3) temporal depth, and (4) insufficient variables. An important exception is Golitko’s study [5] using ethnographic data from New Guinea. He compared material culture networks based on bone dagger attributes with ethnographic interaction records. He found some evidence of correlation between material and social networks, but correlations were modest, and centrality and neighborhood detection performed poorly. Golitko recommended caution in archaeological applications of network methods while still supporting their broader utility. This study is a strong example of how ethnographic data can be used to evaluate relationships between material culture and social networks.

In general, ethnoarchaeological studies are often limited to a single village or a small number of villages. Data collection is time consuming and difficult even at smaller scales. The time depth of these studies is usually limited and is orders of magnitude shorter than the time depth of archaeological studies. Finally, ethnoarchaeological studies often lack the variables needed to answer the questions we are interested in. For example, many studies do not record the social networks related to the material culture they are studying or vice versa.

What is needed is a dataset of material culture that spans several generations, encompasses a large region with sufficient material culture variability to recognize cultural boundaries, and results from the direct social interaction between individuals. If we cannot observe the past or the present to obtain the necessary data, then we are left with the virtual. This is precisely what agent-based models (ABMs) provide.

ABMs have been likened to “behavioral laboratories” [31] where experiments can be conducted under controlled conditions. ABMs are computer simulations where agents make decisions based on a set of rules. Over time these simple rules can lead to complex, emergent behaviors [32].

We are not the first to investigate the relationship between material culture and social networks using an ABM. Work by Gravel-Miguel [6,33], a co-author of the agent-based model discussed herein, found that similarities in artifact attributes can serve as adequate representations of past social interactions, particularly when cultural transmission is a key factor. She found that material culture, influenced by mechanisms like conformism and prestige-based learning, correlates with social networks, though indirect transmission and environmental constraints may complicate the accuracy of these reconstructions, and careful consideration of external factors is necessary for accurate interpretation.

The model used in this analysis, ArchMatNet [30,34,35], is designed to replicate simple social interactions among hunter-gatherers. Elsewhere we used the ArchMatNet model to shed light on how variability in the settlement patterns and mobility strategies among prehistoric societies affected the structure of the archaeological record and our ability to recover past social networks from it [30]. Here, we investigate more broadly how social interaction networks correlate with material culture networks, investigate the correlations between specific activities and material culture, compare the generative properties of each type of network, and investigate the contributions of various parameters from the model.

### Networks in archaeology

Network science approaches to archaeological research continue to grow [36,37] for good reasons. Network methods have already proven useful as tools for exploring and formalizing complex relational patterns in archaeological data across a huge array of contexts, scales, and questions [see 1, 38]. Beyond this, network theory and network thinking also have the potential to help us move beyond some of the “culture-historical baggage” [39], and rigidly defined categories of traditional archaeological models towards an approach that better accommodates the empirical ethnographic and archaeological evidence of variability and fluid boundaries [39–41]. In their critiques of the tacit continuation of culture-history methods in archaeology, Feinman and Neitzel [40] and Holland-Lulewicz [39] suggest relational perspectives, such as social network analysis, are key to moving beyond the rigid categorical approaches currently fundamental to archaeological practice.

The relational perspective fundamental to network approaches holds that we must consider both the nature and structure of interactions among social units as well as the attributes of individuals or groups to fully understand social phenomena [1,42]. Network science is concerned with all aspects of relational data [43], and networks can be generated from many types of archaeological data.

Common network types include material culture [44], movement [45], spatial proximity [46], and inter-visibility [47]. In each of these examples, a person, group, or place is represented by a node and a relationship is created to other nodes. It is the relationship between these nodes that defines the structure of the network.

Relationships are defined in many ways, but here we are interested in similarity networks in which the relationships represent commonalities in the relative frequencies of material culture attributes. Similarities in material culture are seen as a proxy for the probability of a connection between a set of actors or contexts and can be treated as weighted relationships based on the strength of such similarities [48].

Each network can be analyzed visually in a network graph or quantified using various formal metrics, such as centrality scores. Network centrality measures the potential influence of a node on the network and different centrality metrics can be used to characterize different kinds of flows and movements across networks [49]. Nodes that have a high centrality score for a given metric may have more connections than average or may be positioned centrally in the network and thus may gain some advantage based on their position.

Despite the promise of network methods for addressing a broad range of archaeological questions pertaining to interaction, there are many challenges. Even the creation of a network representation can be difficult. In archaeological practice, a network is an abstraction of the phenomena we wish to study [36]. Prignano and colleagues [50] list three common difficulties in creating network representations: (1) defining boundaries, (2) nodes, and (3) relations. None of these are easy tasks to handle, and all existing caveats to archaeological data are applicable to network datasets: missing or incomplete data, inaccurate data, taphonomic issues, taxonomic issues, and others. Some work has been done on the issues mentioned by Prignano and colleagues. Re-sampling networks has shown that many large networks are robust to missing nodes [2,51], as long as the samples are random or non-biased [35]. However, time-averaging in these datasets can significantly reduce the fidelity of network interpretations. Daems and colleagues [52] show that this “palimpsest” effect often leads to overestimating settlement hierarchies and the importance of central hubs.

Even once a network representation has been created, there are many issues regarding its interpretation. Here we are concerned particularly with archaeological similarity networks. These networks are typically created with a site or context as a node and relations are defined based on a similarity measure based on the relative frequencies of different categories of material culture. Rather than interpreting a relation between nodes as the existence of a face-to-face interaction, such as exchange or migration, this tie should be considered a representation of the probability of a connection having existed between the sites [1].

As this literature demonstrates, network analysis in archaeology has not been adopted uncritically, yet there remains more to be done. If similarity networks represent only the probability of interaction, as Peeples indicates, then this leaves an important question: how well does a similarity tie that represents the probability of interaction compare to the actual interaction that took place in the past?

As mentioned, Golitko [5] addressed this question with an ethnoarchaeological study, but he recognizes that the study has limitations. For example, the ethnographic network is composed of binary ties indicating whether a relationship existed between communities. How might the results change if the network were weighted by the frequency of interaction? Eigenvector centrality, one of the centrality measures used by Golitko, is commonly used by archaeologists and is a good way to measure the importance of a node in a network. Calculating the eigenvector centrality for the entire ethnographic network shows that all of the sites that contain bone dagger data had a centrality score of zero. How might the results be different if more central locations in the network had bone dagger data? These questions are not meant to disparage the work of Golitko, but to highlight the complexity of working with network data or any archaeological or ethnographic data. This complexity is why we turned to agent-based models to complement the work of Golitko and others.

### Agent-based models

An agent-based model (ABM) is a computer simulation in which agents are programmed to make decisions based on a set of rules [see 53 for an overview]. Over time these rules can lead to complex, emergent behaviors [32]. Characteristics of emergent behaviors include self-organization, unpredictability, and bottom-up processes. In other words, the end result of the simulation is not preprogrammed into the model but develops as the model progresses from the base set of rules.

ABMs can be useful in many archaeological contexts, although they are “not a miracle cure” or “a panacea,” as Rogers and Cegielski note [54]. They highlight several areas where ABMs are particularly useful, including exploring links between material culture and social dynamics [54]. That is precisely our goal here. ABMs cannot replicate reality or replace empirical data, but they are valuable because they force researchers to state assumptions explicitly. Assumptions can be overlooked in narrative interpretation of archaeological data, but in ABMs they must be implemented directly in the model.

ABMs can be generalized [55–58] or specific and based on empirical data [33,59–61]. In our case, we use a more generalized model. By eliminating variables specific to any one place or time, we can more clearly isolate relationships between material culture and social interaction networks without introducing place-specific variables. This improves experimental control but does not establish that the results apply directly to any empirical case.

## Materials and methods

The ArchMatNet model is a generalized agent-based model written in NetLogo [62] and designed to represent patterns of social interaction and material culture among hunter-gatherers or small-scale horticulturalists (Fig 2). It evaluates hypothetical relationships generated under explicit social, behavioral, and cultural-transmission assumptions rather than replicating a specific empirical society. For a fuller model description and parameter investigation, see Bischoff and Padilla-Iglesias [34] and Supporting Information (S1 Text).

**Fig 2:**
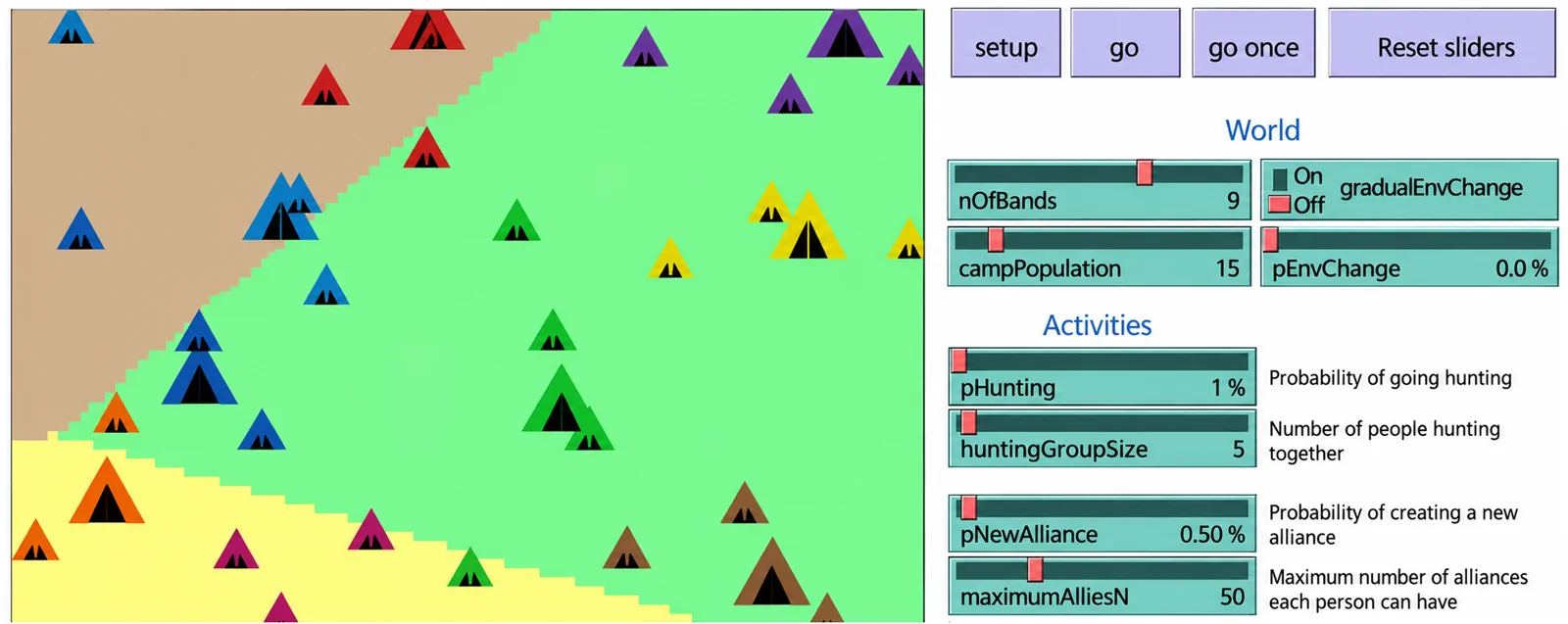
NetLogo interface for the ArchMatNet agent-based model. Small tents represent camps. Large tents represent areas where camps aggregate as bands. The background of the model world represents different environmental areas. Various parameters can be set using sliders or switches, and metrics can be monitored using plots. Only part of the interface is shown to preserve visibility.

### Social structure and spatiotemporal scale

Agents represent individuals organized into hierarchically nested social groups: each agent belongs to a camp, and each band consists of three camps. Bands are spatially dispersed across a virtual landscape, and the simulation proceeds in discrete time steps representing monthly intervals.

The model assumes a one-month timestep to balance temporal resolution and computational efficiency. A typical run includes 4,000 time steps, equivalent to ~333 years, which is appropriate for archaeological time scales. Although camps are dispersed, the model assumes that agents can visit any other camp and return within a month–consistent with estimated round-trip travel distances of ~540 km based on known daily mobility rates [63].

Opportunities for interaction are hierarchically structured to mimic expected real-world social behavior:

- Intra-camp: Most frequent and intense interactions.
- Intra-band: Moderate frequency among agents in different camps of the same band.
- Inter-band: The least frequent interactions, geographically weighted.

Each agent maintains a dynamic personal network of friends/allies. While these relationships can span different camps or bands, intra-band connections dominate, reinforcing stronger cohesion at the band level in line with ethnographic expectations.

### Agent activities and model schedule

We use *interaction* as an umbrella term for four recorded activities: visiting, hunting, trading, and learning. *Aggregation* refers specifically to the periodic gathering of the three camps belonging to a band at a common location. Aggregation creates visiting ties and opportunities for hunting, trading, and learning, but it is not a separate interaction network. The *aggregationFreq* parameter records the number of monthly time steps between these gatherings, so smaller values represent more frequent aggregation.

Each monthly time step proceeds through the following sequence:

1. Agents maintain their personal alliances, may innovate new pot or point traits, and may produce objects using traits they know.
2. If an aggregation is scheduled, agents move to their band’s aggregation location. Co-present agents record visiting ties, may form alliances and trade, and may hunt with bandmates. When an aggregation is not occurring, agents may visit an ally in another camp or hunt with members of their band.
3. Visits and hunts bring agents together and create additional probabilistic opportunities to trade objects and transmit cultural traits. Trading requires both agents to possess an object, while learning depends on the transmission rate and the traits available from co-present agents.
4. Agents may lose a known trait, discard pots or points, and, after the first 1,000 time steps, migrate to another camp. The model accumulates interaction counts and discarded artifacts throughout the run.

### Artifact representation and transmission

Pots and projectile points are represented as abstract artifact classes rather than as specific archaeological forms. Each agent begins knowing one point trait and one pot trait. When *uniqueTraits* is false, all agents begin with the same point and pot traits. When it is true, agents in each camp share starting traits that differ from those assigned to other camps. Thus, the initial number of distinct point traits and pot traits is either one of each or one of each per camp. The number is not fixed over a run: agents can innovate new main types and variants, learn traits from others, and forget traits.

A *type* is a categorical trait that an agent can use to produce an object. Main types are represented by numeric identifiers, and variants add a letter to a main type. Visibility is a property that changes how likely a trait is to be selected during cultural transmission; it is distinct from the difference between pot and point types. High-visibility traits have a greater probability of being copied than low-visibility traits. Agents learn through either conformism (copying common traits among co-present agents) or prestige bias (copying traits from a selected model).

The primary difference between points and pots lies in prestige logic. Prestige for pots is assigned randomly. In contrast, each point type is associated with the environment in which it was innovated. During hunting, an agent’s point prestige depends on the proportion of point types in its inventory that match the current environment, averaged over recent hunts. This adds a functional evolutionary component to points that is not present in pottery in this model.

### Data generation and simulation logistics

As agents interact, pots and points are randomly dropped, forming the virtual archaeological record. All visiting, trading, learning, and hunting events are recorded, producing separate interaction counts for each activity.

To account for the stochastic nature of ABMs [64], we performed over 30,000 simulations using Arizona State University’s Sol supercomputer [65]. This large sample size allows us to explore permutations of parameters (see Table 1) related to population, learning, and activity frequency. Because hunting is restricted to within-band interactions, hunting is not reported as a separate pairwise network comparison; its counts are retained only in the combined interaction matrix.

**Table 1:** Primary ArchMatNet parameters.

Parameter	Default Value	Role in model
aggregationDuration	1	Number of monthly time steps an aggregation lasts
aggregationFreq	12	Number of monthly time steps between band aggregation events; smaller values mean more frequent gatherings
campPopulation	15	Number of agents in each camp
pTrading	5	Probability (out of 100) of trading during each opportunity when co-present agents have objects to exchange
pHunting	1	Probability (out of 100) that an agent initiates a hunt during a time step
learningMethod	50	Probability of prestige-based rather than conformist learning; 0 is entirely conformist and 100 entirely prestige-based
nOfBands	3	Number of bands (three camps are created per band)
nTicks	4000	Number of monthly time steps in the simulation
pMigration	0.05	Probability (out of 100) that migration occurs after the first 1000 time steps
pLuckyLeap	1	Per-agent innovation threshold (out of 1000) for a new main artifact type
pNewObject	10	Probability (out of 100) that an agent produces a new object
pNewVariant	6	Per-agent innovation threshold (out of 1000) for a new variant of a known main type
transmissionRate	5	Probability (out of 100) that cultural transmission occurs during a learning opportunity
uniqueTraits	TRUE	Determines whether all camps begin with the same traits or each camp begins with distinct traits
visibility	TRUE	Determines whether trait visibility changes the probability of cultural transmission
pVisiting	5	Probability (out of 100) that an agent visits an ally during a non-aggregation time step

### Output matrices

The model produces two primary data formats for analysis:

- Raw Data: Cumulative artifact assemblages and social interaction counts per camp.
- Condensed Matrices: Separate matrices of visiting, trading, learning, and hunting counts between camps, together with point- and pot-similarity matrices calculated from discarded artifacts using a weighted Jaccard index.

### Statistical analysis

The goal of this analysis is to compare networks generated from directly modeled social behaviors with networks generated from the material assemblages those behaviors produce. This allows us to assess how well material culture patterns can reconstruct generative social behaviors and to identify potential sources of equifinality.

Numerous methods can be used to compare similarities in network graphs [66]. The applicability of each method depends on the type of graphs being compared and the research questions addressed. In our case, we are comparing two types of networks. The social interaction network represents how often different camps interacted with each other, and the material culture network shows how similar the artifacts from these camps are. The social interaction network is a weighted, undirected graph where the weight of the edge represents the frequency of interaction between two camps. Undirected means that the relationships do not have a specific direction. Weighted means the edge (or relationship) between each node is represented by a value instead of a binary tie that either exists or does not exist. The material culture network is a weighted, undirected graph where the weight of the edge represents the similarity of the material culture between two camps.

The first difficulty we encountered in comparing our networks was the incommensurability of edge weights for the material culture and interaction networks. For the material culture networks, the strength of the connections between camps was determined by how similar the artifacts found in each camp at the end of the model (accumulated through random breaking of artifacts) were to one another. Each pot or point had a type assignment in the model. Similarities between pot and point assemblages were calculated using a weighted Jaccard index. The Jaccard index is a statistic used to measure the similarity between two sets by dividing the size of their intersection by the size of their union [see 34 for a more detailed description]. The interaction networks were created simply by aggregating the counts of each interaction of the given type (learning, trading, visiting, or hunting). Table 2 summarizes each network. Given that interaction intensity or similarity is the desired output, transforming the edge weights into z-scores is appropriate. Z-scores help standardize the data by showing how much each value differs from the mean, making it easier to compare different networks. Z-scores were calculated for each edge weight in the networks, which were then scaled from 0 to 1 to facilitate easier interpretation of network metrics. This method makes the edge weights more suitable for network comparison methods.

**Table 2:** ArchMatNet network types and edge definitions.

Network	Description
Learning	Edge weights count successful cultural-transmission events between agents from each pair of camps
Trading	Edge weights count exchanges of pot or point objects between agents from each pair of camps
Visiting	Edge weights count visiting contacts between agents from each pair of camps during ally visits or band aggregations
Point	Edge weights are weighted Jaccard similarities between discarded point assemblages from each pair of camps
Pot	Edge weights are weighted Jaccard similarities between discarded pot assemblages from each pair of camps

Three methods were used to compare and analyze networks: graph correlation, the quadratic assignment procedure (QAP), and exponential random graph models (ERGMs). Graph correlation was implemented in the *sna* package [67] in R [68]. This measure is equivalent to Pearson’s product-moment correlation and ranges from 1 (perfect correlation) to −1 (perfect negative correlation), indicating agreement in edge presence/absence or edge weights across two graphs with the same nodes.

A QAP test [69] uses Monte Carlo permutations to evaluate whether the alignment of nodes and edges across two networks is stronger than expected under random relabeling. We calculated the graph correlation between two matrices, repeatedly permuted their row and column labels, and recalculated the correlation after each permutation. The analysis used the *qaptest* function in the *sna* package with *gcor* as the test statistic. The supplemental analysis code records the function’s pleeq value: the proportion of permuted correlations less than or equal to the observed graph correlation. Values close to 1 indicate that the observed correlation exceeds nearly all correlations obtained after random relabeling.

The final method of comparison was the use of ERGMs as implemented in the *ergm* package [70,71] in R. ERGMs help us understand how different parts of a network, such as nodes and connections, form by estimating the processes that may have shaped the observed network structure. This enabled us to test whether certain network features, like the connections between camps, were likely due to chance or driven by underlying interaction patterns. Although ERGMs are not commonly used in archaeology [72–75], they are valuable for modeling relationships between camps in our study. By estimating parameters that describe the processes likely to have generated the network, ERGMs allow for the simulation of multiple networks, which can then be compared to the observed network to assess the likelihood that observed patterns are due to specific generative processes. These models also allow for the inclusion of various variables to predict the network structure, with different models being compared to determine the best fit.

## Results

### Network comparisons

The first question we address is the overall relationship among the different networks produced by the model. These include three interaction networks (visiting, trading, and learning) and two material culture similarity networks (pots and points). In the model, pots and points differ in prestige transmission: pot prestige is randomly assigned, whereas point prestige depends on hunting success. Point attributes are also adapted to specific environments, which increases hunting success.

Fig 3 illustrates the correlations between pairs of networks (refer to Table 2 for network descriptions; the numerical scores are listed in Table 3). The visiting and trading networks have almost identical edge patterns, with a correlation approaching 1.0. Learning is also closely correlated with both visiting and trading, but to a lesser degree. The six comparisons between the three interaction networks (visiting, trading, and learning) and the two material culture networks (points and pots) have lower but still strong mean correlations greater than 0.75; the correlation between the point and pot networks is similar. Comparisons involving material culture also have larger ranges and more outliers, as expected given the stochasticity built into the ABM and the more than 30,000 simulations.

**Table 3:** Pairwise network comparison results.

Network Comparison	Mean Graph Correlation	Mean QAP Lower-Tail Probability (pleeq)
Point vs. combined interaction	0.747	0.996
Pot vs. combined interaction	0.749	0.996
Point vs. pot	0.756	0.997
Learning vs. point	0.762	0.997
Learning vs. pot	0.764	0.998
Visiting vs. pot	0.767	0.997
Trading vs. pot	0.767	0.998
Visiting vs. point	0.769	0.997
Trading vs. point	0.770	0.997
Trading vs. learning	0.965	0.999
Visiting vs. learning	0.966	0.999
Visiting vs. trading	0.999	1.000

**Fig 3:**
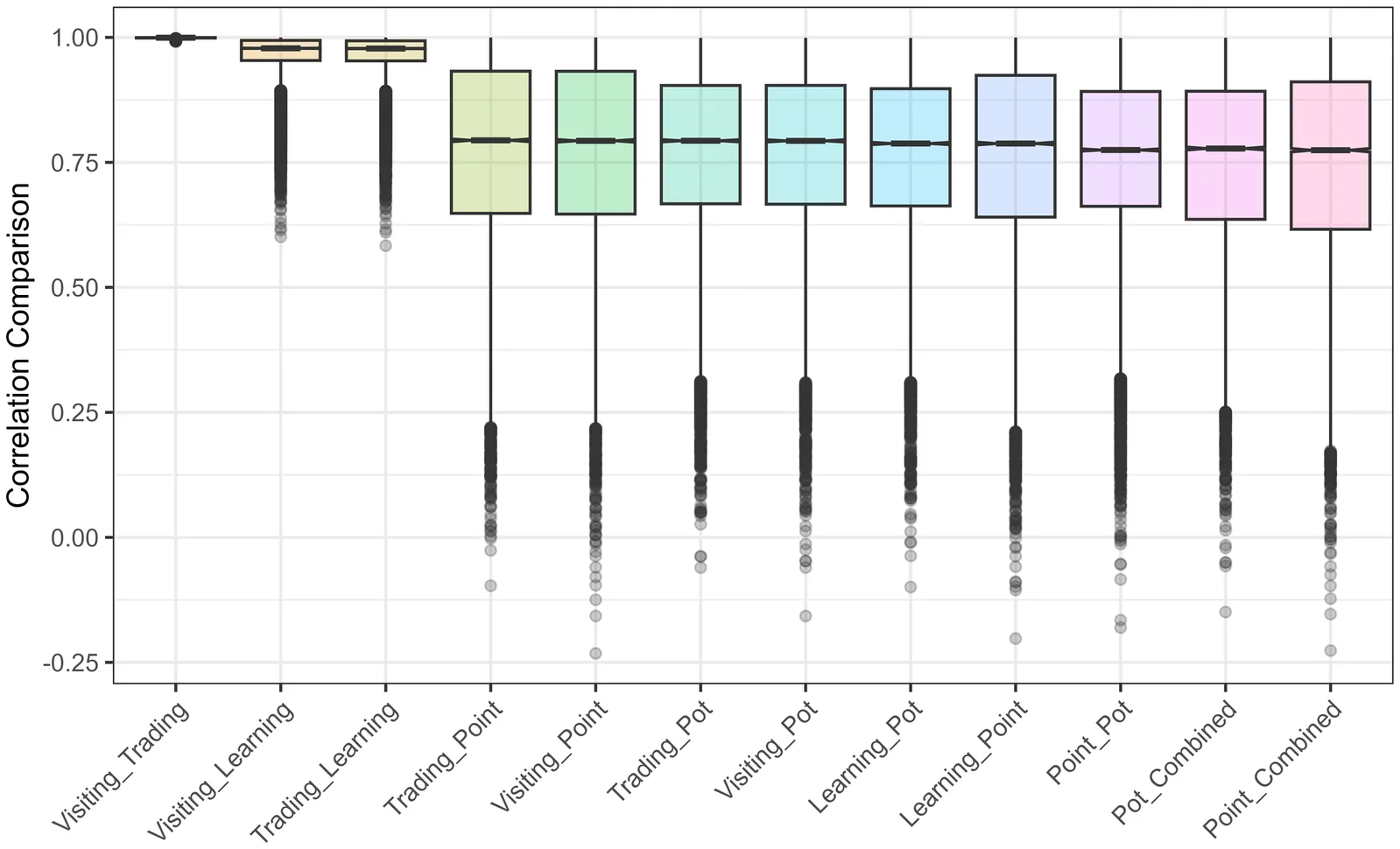
Distributions of graph correlations between pairs of visiting, trading, learning, point-similarity, and pot-similarity networks generated by ArchMatNet. Interaction-to-interaction comparisons are shown separately from comparisons involving material culture networks.

The high correlations among visiting, trading, and learning are partly a predictable consequence of the model structure. Visits and aggregations create co-presence, which in turn creates opportunities for trading and learning. These networks are therefore not independent. Their correspondence is not completely deterministic, however, because trading requires appropriate inventories and is controlled by *pTrading*, while learning depends on the transmission rate, available traits, visibility, and learning method. We treat these interaction-to-interaction correlations primarily as a model diagnostic. The more substantively informative comparisons are those between interaction networks and the material culture networks they generate.

The “Point vs. combined interaction” and “Pot vs. combined interaction” rows in Table 3 compare each artifact-similarity matrix with a combined interaction matrix constructed by summing visiting, trading, learning, and hunting counts for each pair of camps. These comparisons produced the lowest mean graph correlations, although the values were close to those obtained when points and pots were compared with each interaction type separately. Because archaeological datasets may reflect multiple forms of interaction, these results suggest limited ability to distinguish among interaction types using this approach alone.

The lower-tail QAP probabilities in Table 3 are consistent with the graph correlation results. Values close to 1.0 mean that the observed graph correlations exceed nearly all correlations generated by randomly relabeling the nodes.

Outliers shown in Fig 3 were investigated further. Some can be attributed to runs where trading probability was zero. In those runs, the average correlation between visiting and point networks was 0.12, much lower than values observed under other parameter settings. Because trading is one of three mechanisms for inter-camp material transmission (along with learning and migration), this effect is expected. However, relatively few runs had zero trading probability, and many runs with default trading probability also produced low correlations. Runs with high probabilities of creating new objects also showed lower correlations, likely because large virtual assemblages make interaction markers harder to detect. This finding has implications for interpreting large archaeological assemblages. Notably, no outliers occurred in runs where all parameters were set to default values.

We expected differences in how interaction and material culture networks would correlate. However, as shown in Fig 3 and Table 3, correlation values were similar across interaction and material culture types. In this model, we therefore find limited evidence that one interaction type better predicts material culture networks than another, likely because trading and learning co-occur with visiting. Several parameters did affect network correlations, and these effects were stronger than those observed in initial model testing [34].

### Model parameters

All model parameters were tested to evaluate their effects on network correlations. In ABMs, parameters are values that vary between simulations and shape agent behavior. Parameters discussed here are listed in Table 1. The full parameter list is provided in S1 Table, and parameter roles are described by Bischoff and Padilla-Iglesias [34]. Mobility-related parameters (aggregation frequency, population size, migration rates, and visiting frequency) were tested in additional runs and are discussed elsewhere [see 30].

One of the key factors affecting the correlation between material culture and interaction networks is whether all agents start with the same types of pots and points or if each camp begins with its own unique set of traits. This significantly affected how well the material culture networks aligned with the interaction networks. In other words, does every agent make the same type of pot and point or are agents making different types of pots and points depending on the camp they belong to? The average graph correlation value between visiting interaction networks and point networks is 0.16 when the starting traits are the same versus 0.77 when the starting traits are unique per camp. The graph correlation for every agent starting with the same trait is almost nonexistent when compared to the strong correlation that exists when each camp has unique starting traits.

Clearly, differences in material culture are necessary to use material culture networks as a representation of social interaction networks. This effect has been observed in other network studies using artifact similarity or geochemical sourcing [e.g., 5,76–79]. These studies indicate that few sources of artifacts or indistinct sources of artifacts create uniformity in the network. For this reason, all results shown in this analysis, including the graph correlations shown previously, only include model runs with unique traits.

Isolating the effect of a single parameter is challenging when multiple parameters vary simultaneously. To estimate parameter effects, we fit model parameters to graph correlation values (the dependent variable) using generalized additive models (GAMs). GAMs can capture complex nonlinear relationships and handle both categorical and continuous predictors [80]. This makes GAMs well suited for evaluating how ArchMatNet parameters influence the relationship between social interaction and material culture.

ArchMatNet includes 24 adjustable parameters. When all are included in a GAM (excluding *uniqueTraits*, which is fixed at 1 so camps begin with different traits), the $r^{\text{2}}$ from the *gam* function in *mgcv* [81] is 0.451 (full results in S2 Table). Thus, these parameters explain nearly half of the variance, with the remainder attributable to model stochasticity. When most parameters are removed, $r^{\text{2}}$ decreases only to 0.372 with four remaining parameters (*aggregationFreq*, *learningMethod*, *nTicks*, and *visibility*). Removing 19 parameters reduces explained variance by only about 10 percentage points, suggesting limited sensitivity to most parameters.

The variables with the strongest effects are shown in Fig 4. Because *aggregationFreq* records the number of monthly time steps between gatherings, lower values represent more frequent aggregation. Correlations were strongest at six-month intervals and declined as the interval increased to 12 and 24 months. Within this model, more frequent aggregation therefore produces stronger correlations between visiting and material culture networks.

**Fig 4:**
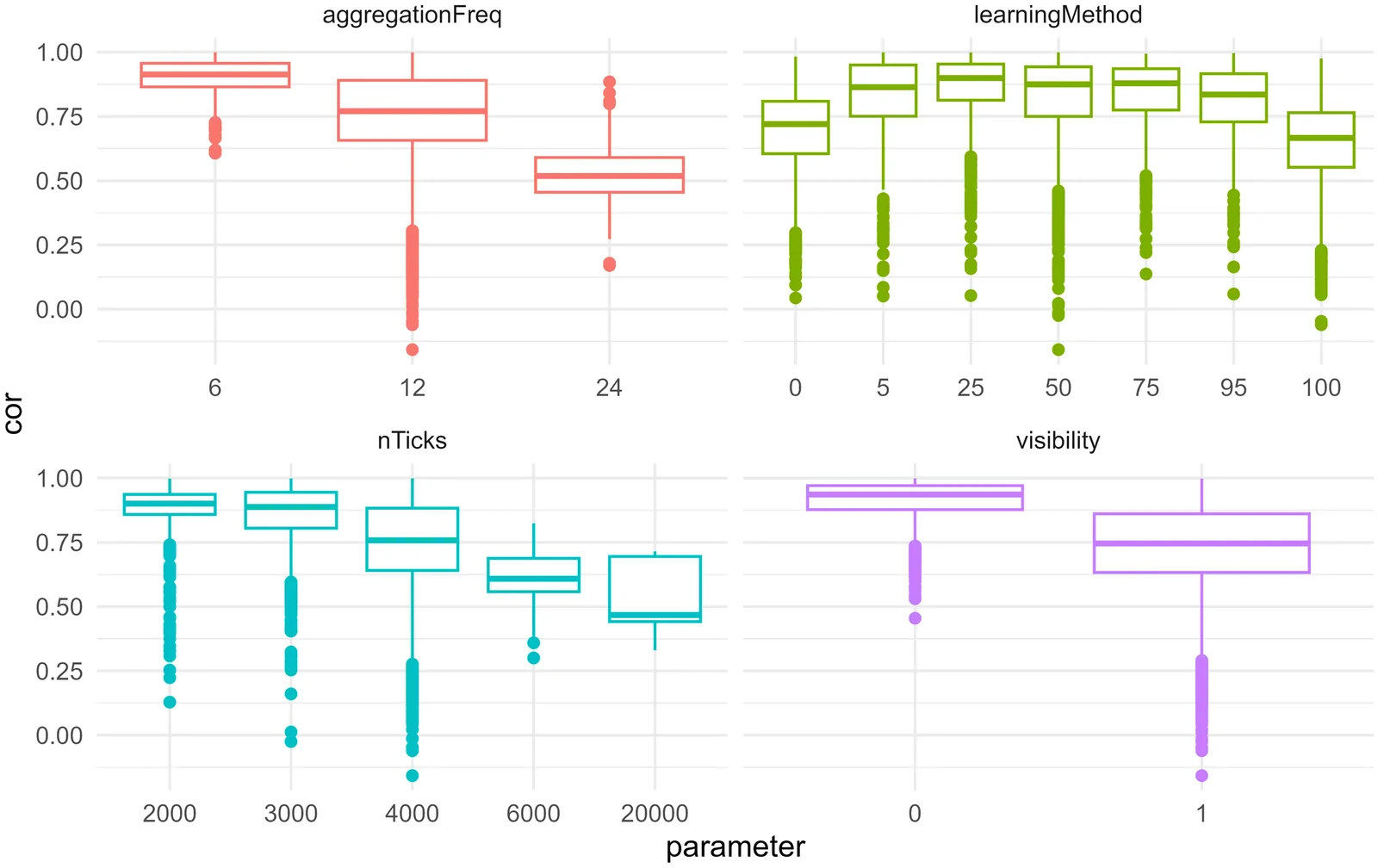
Boxplots showing graph correlations between the visiting interaction network and point material culture similarity network for the four parameters with the strongest effects in the generalized additive model (GAM). For *aggregationFreq*, values are monthly time steps between gatherings, so smaller values represent more frequent aggregation. For *visibility*, 0 indicates that visibility does not affect transmission and 1 indicates that it does.

The *learningMethod* was formatted as a factor in the GAM model, as the values represent a continuum from 0 where all learning is based on conformism to 100 where all learning is based on prestige. A value of 50 indicates that learning is split between the two methods. As shown in Fig 4, *learningMethod* has a nonlinear relationship where equal learning methods have the strongest correlations, but the correlation values decrease as the learning method leans towards one method or the other. Prestige learning does result in a slightly lower correlation than conformism.

The *nTicks* parameter controls the number of model time steps. Here, each tick represents one month. The shortest run length, 2000 ticks, corresponds to nearly 167 years. As run length increased, correlation values decreased, indicating that material culture networks were less correlated with interaction networks over longer timespans.

The *visibility* parameter controls the likelihood that certain traits are copied. When *visibility* is 0 the correlation between networks is stronger. Thus, the introduction of low-visibility traits introduces more variability in the network comparison, although the relationship is still strong.

Other variables that may have an impact–though smaller than those discussed here according to the GAM–include population size and probabilities of interaction and learning. Regardless of the parameters, with the major exception of *uniqueTraits*, most runs of the model have significant correlations between the interaction networks and material culture networks.

### ERGMs

The ERGM analysis addresses a different question from graph correlation and QAP: whether interaction and material culture networks share higher-order structural patterns after their edge weights are binarized. ERGMs estimate which local configurations can generate an observed network. Similar well-fitting configurations across interaction and material culture networks would indicate that the simulated networks share structural tendencies, not that they are identical.

Three models were used in this analysis, each incorporating an edge parameter, which is a default for ERGM analyses since a network cannot exist without edges. Each model also includes a band parameter, which assigns each node (representing a camp) to a specific band. This is important because the band each camp belongs to affects interaction frequencies. The triangle parameter captures triadic (friend-of-a-friend effect) structures where three nodes are all mutually connected, a common feature in networks.

In this ABM, each band consists of three camps, always forming a triad of strong interactions. Two related parameters, GWESP (geometrically weighted edgewise shared partners) and threetrail (two-paths or three trails), were used to capture specific network structures. GWESP measures the likelihood that two nodes sharing a common partner will form a triangle. This parameter reflects the tendency for camps connected to the same camp to form direct links between themselves. The threetrail parameter, on the other hand, identifies network structures where three nodes are connected by two edges, without forming a triangle. It captures paths to triads through intermediate nodes and is used to detect strong connections between camps from different bands, helping to form a broader network structure.

The three models together are:

1. edge + band
2. edge + gwesp + threetrail + band
3. edge + triangle + threetrail + band

ERGM fit was assessed with the Akaike Information Criterion (AIC). An AIC value is not inherently high or low and is meaningful only in comparison with candidate models fitted to the same network and data. Within each network, the model with the lowest AIC has the strongest relative support among the models compared. AIC values for the point and visiting networks should not be compared directly because they come from separate fits. We also used goodness-of-fit (GOF) tests to evaluate how well each ERGM reproduced structural properties of the observed network.

ERGMs are computationally intensive. A random sample of 100 model runs was selected and each model run was fitted to the three models for each of the five types of networks returned from ArchMatNet (visiting, trading, learning, point, and pot). Each ERGM was run for the default 1024 iterations, resulting in at least 2,000,000 total simulations depending on how many iterations each ERGM requires (between 1 and 60). Unfortunately, the *ergm* function in R has a tendency to fail when trying to replicate certain network structures and some ERGMs did not return results.

A final note for the ERGMs is that they require binary networks. All network weights were binarized by calculating the 75th quantile for the edge weights and retaining links above that quantile. This is an arbitrary cutoff but it can be useful in identifying the structural relationships among the strongest ties [see 48].

Table 4 shows the ERGM results. For clarity, only point and visiting networks are shown; the remaining networks returned similar patterns and can be explored in the Supporting Information analysis code. The GOF probabilities are high for all reported models, indicating no detected lack of fit for the tested statistics (GOF plots are provided in S1 Fig). For the point network, the triangle and threetrail model has the lowest AIC (89.25), compared with 95.38 for the GWESP and threetrail model and 104.43 for the edge-plus-band model. The triangle and threetrail model therefore has the strongest relative support among the three point-network models. For the visiting network, the GWESP and threetrail model has the lowest AIC (93.10), compared with 96.44 for the triangle and threetrail model and 106.64 for the edge-plus-band model. The GWESP and threetrail model therefore has the strongest relative support among the three visiting-network models. In both networks, models representing triadic closure and intermediary paths have stronger relative support than the edge-plus-band baseline. This is consistent with the three-camp structure of each band.

**Table 4:** ERGM Results.

Network	Formula	GOF	AIC
Point	Edge + band	0.98	104.43
Point	Edge + GWESP + threetrail + band	0.99	95.38
Point	Edge + triangle + threetrail + band	0.98	89.25
Visiting	Edge + band	0.98	106.64
Visiting	Edge + GWESP + threetrail + band	1.00	93.10
Visiting	Edge + triangle + threetrail + band	1.00	96.44

Both of the best-supported model specifications include the threetrail parameter. In ArchMatNet, this term represents intermediary paths created when some camps form weaker between-band ties while other camps lack those ties. Its presence in both best-supported specifications indicates that camps acting as intermediaries are an important part of the simulated network structure. Because the candidate specifications combine several terms, however, the AIC comparisons do not isolate an independent effect of threetrail.

After identifying well-fitting ERGM specifications, we generated simulated networks that replicated key structural properties of observed networks. We then compared correlations between simulated and observed networks. For 10,000 simulated random visiting networks, the average graph correlation with the observed visiting network was 0.15, with a maximum of 0.55. This is far below the near-1 correlations observed among ArchMatNet interaction networks. Comparing 10,000 simulated point networks with the observed visiting network yielded an average correlation of 0.05 and a maximum of 0.5. These tests indicate that the high correlations reported above are unlikely to be chance outcomes.

### Network centrality

Centrality measures are often central to network analysis. We calculated the same measures used by Golitko [5]: betweenness, closeness, degree, and eigenvector centrality. These results highlight a limitation of ArchMatNet. Because the network is highly symmetrical (each camp has two strong within-band ties and weaker ties to other bands), centrality scores are tightly clustered. For example, in a sample run, eigenvector centrality ranges from 0.15 to 0.24 and degree centrality from 4.0 to 4.1. Betweenness and closeness show similarly narrow ranges. With so little variation, correlations between centrality scores in interaction and material culture networks are weak (approximately −0.2 to 0.2). We restricted this analysis to runs with four or more bands to increase variation, but adding bands had little effect and in some cases reduced correlation. This limitation of a generalized ABM suggests that additional model complexity is needed to better match empirical centrality structure.

### Individual networks

While comparing thousands of model runs is essential for statistical validity, examining individual networks is also useful for understanding structure. Fig 5 compares networks without unique cultural traditions (*uniqueTraits* = false; panel a) and with unique cultural traditions (*uniqueTraits* = true; panel b). In panel (a), all camps begin with identical traits; in panel (b), each camp begins with distinct traits. All five network types used in this analysis are shown.

**Fig 5:**
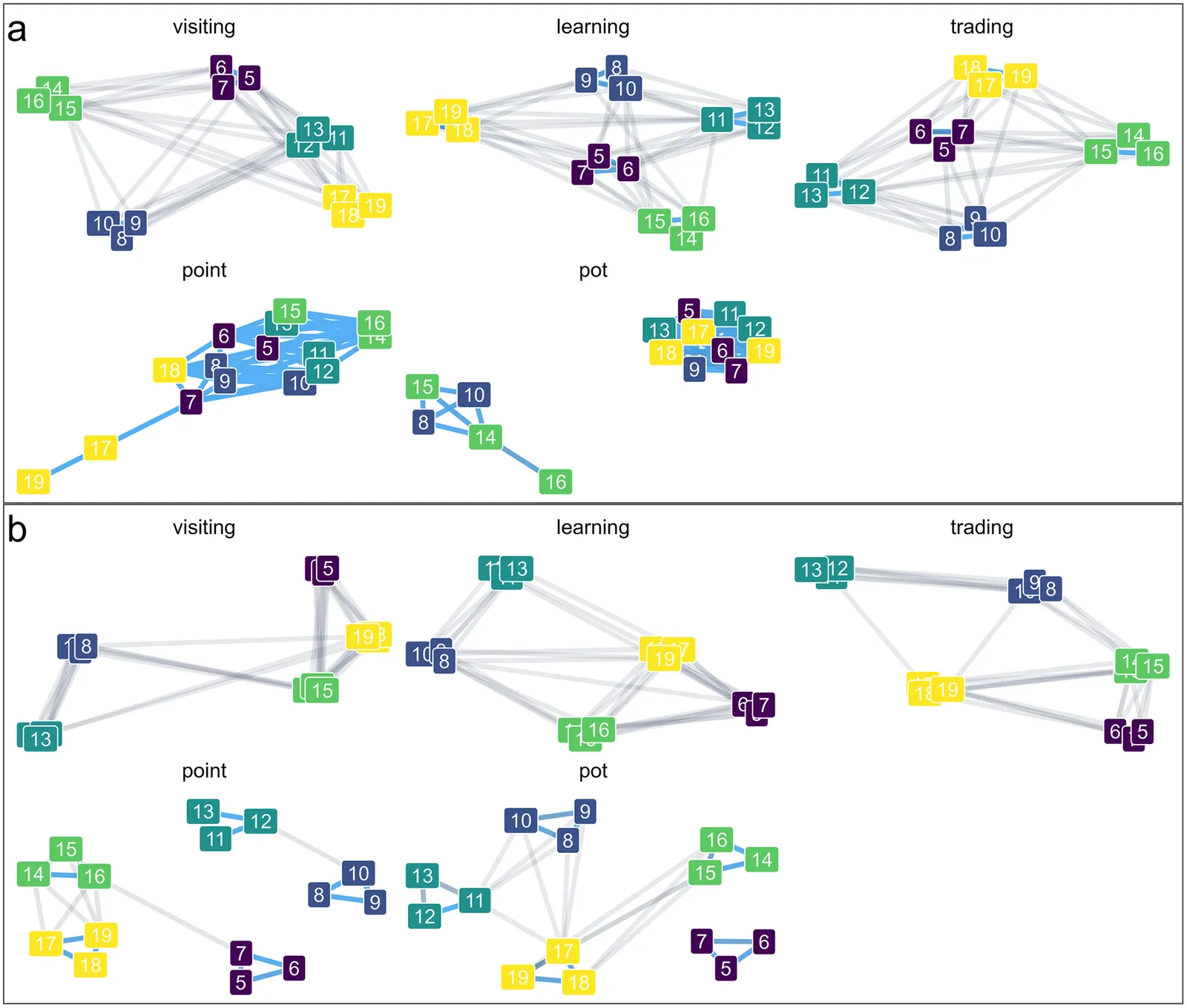
Interaction and material culture networks from two ArchMatNet runs. In panel (a), uniqueTraits is false and every camp begins with identical traits. In panel (b), uniqueTraits is true and each camp begins with distinct traits. Nodes represent camps, are colored by band, and are labeled with camp numbers. Only the strongest 50% of links are shown; edge color and width represent relationship strength.

The visiting, learning, and trading networks are all comparable across both model runs. There is a significant difference between the point and pot runs. For the networks that do not have unique starting traits, the point and pot networks are a jumbled mess. The network structure in no way resembles the interaction networks and in the case of the pot network, no differentiation is possible at all, even with only the strongest 50% of links shown in the network graphs. On the other hand, the pot and point networks with unique starting traits closely resemble the interaction networks. All of the intra-band triads are represented. There are a number of differences in the weaker connections between camps and bands. Besides the necessity of having material culture networks with enough variation, the point and pot networks with unique starting traits demonstrate that these networks struggle with exact replication of weaker links.

Fig 3 shows that the pot and point networks had the weakest correlations (although they were still high). Caution should be applied when interpreting weak connections between networks based on material culture. These weak connections can be important parts of network analysis, but material culture may not always be adequate for determining specific weak relationships between nodes.

A final observation is that point and pot networks differ in some details despite sharing the same major relationships. These differences imply that combining multiple material culture classes may provide a more complete representation of interaction.

## Discussion

This study uses ArchMatNet to investigate hypothetical relationships between social interaction and material culture networks. By simulating these networks under varied conditions and analyzing them with graph correlations, generalized additive models (GAMs), and exponential random graph models (ERGMs), we identify conditions under which material culture can serve as a proxy for social interaction within this model.

Our main findings can be summarized in three points.

1. Within the simulated system, material culture networks (i.e., artifact-similarity networks between camps) can approximate social interaction networks (i.e., counts of interactions between individuals from different camps), provided that material culture variation is sufficiently high, as controlled here by *uniqueTraits*. We do not yet quantify how much variation is required; this remains an important area for future empirical work and may vary by region and period. The observed graph correlations exceeded nearly all values obtained after random relabeling in the QAP tests.
2. Material culture networks are less precise than social interaction networks in these simulations (see Figs 3 and 5). Correlations between material and social networks were weaker than correlations among interaction networks, indicating that artifact-based reconstructions can blur relationship strength. Strong ties may appear weaker, and weak or absent ties may appear stronger. Even so, the strongest relationships generally remained strong in both network types.
3. Different simulated material culture classes can yield different network signals. Although point and pot networks were broadly similar, detailed comparisons (see Fig 5) showed differences in the strongest inter-band ties. This result suggests that using multiple material culture classes may improve reconstruction of interaction networks, but this expectation requires testing with empirical cases.

The model results also show that despite the stochastic nature of agent-based models, certain parameters consistently affected the correlation between social and material networks. Specifically, the interval between aggregations, learning method, number of time steps, and visibility of traits shape these simulated relationships. Because *aggregationFreq* measures the time between gatherings, the results indicate that more frequent aggregation produces stronger correspondence between visiting and material culture networks. This is a conditional result of the model rather than evidence that any particular empirical population followed the same relationship.

Model sensitivity to learning method also underscores the importance of transmission processes. Gravel-Miguel [33] found that even low rates of cultural transmission produced similarities between reconstructed and observed social networks. The nonlinear relationship between learning method (conformist vs. prestige-based) and network correlation suggests that a balance between these strategies produces the strongest correspondence.

This pattern aligns with ethnographic studies of cultural transmission, which also find that mixed prestige-conformist models often perform best [29,82–84]. The mechanisms in those studies differ from those in ArchMatNet, but the broader relationship is similar. In this ABM, low prestige transmission appears to limit trait spread, whereas high prestige transmission appears to spread traits too broadly for material culture to track social processes closely. In ethnographic contexts, conformism helps maintain distinct cultural identities while prestige supports the spread of advantageous traits.

Trait visibility also emerges as a critical factor: enabling differential learning by trait visibility decreases correlation between interaction and material culture networks. This suggests that visibility introduces additional variability into network structure. Current ArchMatNet outputs do not yet allow independent testing of high- and low-visibility trait effects; future work should evaluate these pathways directly.

The ERGMs provide a complementary structural comparison. Within ArchMatNet, models containing triadic closure and intermediary paths received stronger relative support than the edge-plus-band baseline for both visiting and point networks. This indicates that the simulated interaction and material culture networks share some higher-order structural tendencies. It does not establish that the same generative processes operated in empirical social systems.

The results suggest that ArchMatNet is a useful tool for investigating hypothetical relationships between social and material culture networks, but they also highlight key limitations. The most important is the model’s nearly regular network structure: each camp has two strong within-band ties and broadly similar, weaker ties to camps in other bands. This symmetry produces too little centrality variation for robust comparisons across network types. Future model development should prioritize stronger variation in long-distance ties.

The model also does not represent avoidance behavior, even though avoiding a camp may be as socially important as visiting one. People may seek or avoid camps for many reasons. Adding preferential attachment for some inter-band ties while allowing avoidance among other nodes could increase structural variation and improve evaluation of node position in the network. A further necessary step is to test ArchMatNet against ethnographic or archaeological cases with independently documented interaction patterns. An inverse or retrofitting application could also begin with an observed material culture network and evaluate which parameter combinations reproduce it, followed by assessment of whether those combinations are plausible for the case.

ArchMatNet shows how assumptions about interaction opportunities, learning strategies, trait visibility, and material variation can produce stronger or weaker correspondence between social and material culture networks in a controlled simulated setting. These conditional results help identify assumptions that should be examined when archaeological similarity networks are interpreted, while empirical validation remains necessary before the relationships are applied to particular past societies.

## Supporting information

S1 Text ArchMatNet ODD protocol and extended model documentation. Extended documentation of the model’s purpose, entities, processes, and scheduling.
(PDF)

S1 Table Full list of ArchMatNet parameters and default values. Parameter names, descriptions, and default values used in the simulations.
(CSV)

S2 Table Full generalized additive model (GAM) results for parameter effects on graph correlation. Complete GAM output for the parameter-effect analysis.
(CSV)

S1 Fig ERGM goodness-of-fit plots for simulated and observed networks. Goodness-of-fit plots for simulated and observed networks.
(PDF)

## References

[1] Peeples MA. Finding a place for networks in archaeology. J Archaeol Res. 2019;27(4):451–99. doi: 10.1007/s10814-019-09127-8

[2] Peeples MA, Mills BJ, Haas WRJ, Clark JJ, Roberts JMJ. Analytical Challenges for the Application of Social Network Analysis in Archaeology. In: Brughmans T, Collar A, Coward F, editors. The Connected Past: Challenges to Network Studies in Archaeology and History. Oxford: Oxford University Press; 2016. pp. 59–84.

[3] Peeples MA, Roberts JR, Yin Y. Challenges for network research in archaeology. In: Peeples MA, Munson J, Mills B, Brughmans T, editors. The Oxford Handbook of Archaeological Network Research. Oxford University Press; 2024. pp. 34–49. doi: 10.1093/oxfordhb/9780198854265.013.2

[4] Sindbæk SM. Networks and nodal points: The emergence of towns in early Viking Age Scandinavia. Antiquity. 2007;81(311):119–32. doi: 10.1017/s0003598x00094886

[5] Golitko M. A performance test of archaeological similarity-based network inference using New Guinean ethnographic data. J Anthropol Archaeol. 2023;72:101550. doi: 10.1016/j.jaa.2023.101550

[6] Gravel-Miguel C, Coward F. Palaeolithic social networks and behavioural modernity. In: Peeples MA, Munson J, Mills B, Brughmans T, editors. The Oxford Handbook of Archaeological Network Research. Oxford: Oxford University Press; 2024. pp. 443–58.

[7] Gosselain OP. Materializing identities: An African perspective. J Archaeol Method Theory. 2000;7(3):187–217. doi: 10.1023/a:1026558503986

[8] Hegmon M. Archaeological research on style. Ann Rev Anthropol. 1992;21:517–36. doi: 10.1146/annurev.anthro.21.1.517

[9] Hodder I. Symbols in Action: Ethnoarchaeological Studies of Material Culture. Cambridge: Cambridge University Press; 1982. Available from: https://market.android.com/details?id=book-TSE9AAAAIAAJ

[10] Peeples MA. Connected Communities: Networks, Identity, and Social Change in the Ancient Cibola World. Tucson: University of Arizona Press; 2018. Available from: https://market.android.com/details?id=book-fyw-DwAAQBAJ

[11] Wiessner P. Style and social information in Kalahari San projectile points. Am Antiq. 1983;48:253–76. doi: 10.2307/280450

[12] Sackett JR. Approaches to style in lithic archaeology. J Anthropol Archaeol. 1982;1(1):59–112. doi: 10.1016/0278-4165(82)90008-3

[13] Sackett JR. Isochrestism and style: A clarification. J Anthropol Archaeol. 1986;5(3):266–77. doi: 10.1016/0278-4165(86)90008-5

[14] Wiessner P. Style or isochrestic variation? A reply to sackett. Am Antiq. 1985;50(1):160–6. doi: 10.2307/280643

[15] Carr C. Building a unified middle-range theory of artifact design. In: Carr C, Neitzel JE, editors. Style, society, and person. Boston: Springer; 1995. pp. 151–70. doi: 10.1007/978-1-4899-1097-4_6

[16] Carr C. A unified middle-range theory of artifact design. In: C C, Neitzel JE, editors. Style, society, and person. Boston: Springer; 1995. pp. 171–258. doi: 10.1007/978-1-4899-1097-4_6

[17] Clark JJ. Tracking Prehistoric Migrations: Pueblo Settlers Among the Tonto Basin Hohokam. Tucson: University of Arizona Press; 2001. Available from: https://play.google.com/store/books/details?id=27Tk3xNT-6UC

[18] Dietler M, Herbich I. Habitus, Techniques, Style: An Integrated Approach to the Social Understanding of Material Culture and Boundaries. In: Stark MT, editor. The Archaeology of Social Boundaries. Washington, DC: Smithsonian Institution; 1998. pp. 232–63.

[19] Huntley DL. Ancestral Zuni Glaze-Decorated Pottery: Viewing Pueblo IV Regional Organization through Ceramic Production and Exchange. Tucson: University of Arizona Press; 2008.

[20] Lemonnier P. The study of material culture today: Toward an anthropology of technical systems. J Anthropol Archaeol. 1986;5(2):147–86. doi: 10.1016/0278-4165(86)90012-7

[21] Lyons PD. Ancestral Hopi Migrations. 1st ed. Tucson: University of Arizona Press; 2003.

[22] Neuzil AA. In the Aftermath of Migration: Renegotiating Ancient Identity in Southeastern Arizona. Tucson: University of Arizona Press; 2008.

[23] Wobst HM. Stylistic Behavior and Information Exchange. In: Cleland CE, editor. For the Director: Research Essays in Honour of the Late James B Griffin. Ann Arbor: University of Michigan; 1977. pp. 317–42.

[24] Van Oyen A. Historicising material agency: From relations to relational constellations. J Archaeol Method Theory. 2016;23:354–78. doi: 10.1007/s10816-015-9244-0

[25] Wenger E. Communities of Practice: Learning, Meaning, and Identity. Cambridge: Cambridge University Press; 1998. Available from: https://valenciacollege.edu/faculty/development/teaching-learning-academy/documents/CommunityofPractice.pdf

[26] Henrich J. Cultural group selection, coevolutionary processes and large-scale cooperation. J Theor Biol. 2004;220:577–90.

[27] Morgan TJH, Cross CP, Rendell LE. Nothing in human behavior makes sense except in the light of culture: Shared interests of social psychology and cultural evolution. Evolutionary Perspectives on Social Psychology. Cham: Springer International Publishing. 2015. pp. 215–28. doi: 10.1007/978-3-319-12697-5_17

[28] Muthukrishna M, Morgan TJH, Henrich J. The when and who of social learning and conformist transmission. Evol Hum Behav: Off J Hum Behav Evol Soc. 2016;37:10–20. doi: 10.1016/j.evolhumbehav.2015.05.004

[29] Henrich J, McElreath R. The evolution of cultural evolution. Evol Anthropol. 2003;12(3):123–35. doi: 10.1002/evan.10110

[30] Padilla-Iglesias C, Bischoff RJ. Hunter-gatherer mobility patterns influence the reconstruction of social networks from archaeological assemblages. J Archaeol Sci: Rep. 2024;59:104798. doi: 10.1016/j.jasrep.2024.104798

[31] Premo LS. Agent-based models as behavioral laboratories for evolutionary anthropological research. Arizona Anthropol. 2006;17:91–113.

[32] Epstein JM. Generative Social Science: Studies in Agent-Based Computational Modeling. Princeton University Press; 2006. Available: https://play.google.com/store/books/details?id=T8\_zh3F7rMIC

[33] Gravel-Miguel C. The impacts of geography and climate change on magdalenian social networks. Tempe: School of Human Evolution; Social Change, Arizona State University; 2017. http://hdl.handle.net/2286/R.I.44052

[34] Bischoff RJ, Padilla-Iglesias C. A description and sensitivity analysis of the ArchMatNet agent-based model. PeerJ Comput Sci. 2023;9:e1419. doi: 10.7717/peerj-cs.1419 37547383 PMC10403171

[35] Bischoff RJ, Padilla-Iglesias C, Gravel-Miguel C. Evaluating the Effects of Randomness on Missing Data in Archaeological Networks. CAA 2021 Conference: Limassol, Cyprus (in press). CAA; 2021. doi: 10.31235/osf.io/vdy9b

[36] Collar A, Coward F, Brughmans T, Mills BJ. Networks in archaeology: Phenomena, abstraction, representation. J Archaeol Method Theory. 2015;22(1):1–32. doi: 10.1007/s10816-014-9235-6

[37] Brughmans T, Peeples M. Trends in archaeological network research: A bibliometric analysis. J Histor Netw Res. 2017;1:1–24. doi: 10.25517/jhnr.v1i1.10

[38] Peeples MA, Munson J, Mills B, Brughmans T. Introduction. In: Peeples MA, Munson J, Mills B, Brughmans T, editors. The Oxford Handbook of Archaeological Network Research. Oxford University Press; 2024. pp. 1–14. https://books.google.com/books?hl=en&lr=&id=2Q3iEAAAQBAJ&oi=fnd&pg=PA103&ots=ZpuHBWJxOc&sig=BDb3npMKg--sZdgqDIgpKIngqpI

[39] Holland-Lulewicz J. From categories to connections in the archaeology of eastern North America. J Archaeol Res. 2021;29(4):537–79. doi: 10.1007/s10814-020-09154-w

[40] Feinman GM, Neitzel JE. Excising culture history from contemporary archaeology. J Anthropol Archaeol. 2020;60:101230. doi: 10.1016/j.jaa.2020.101230

[41] Terrell JE, Golitko M, Dawson H, Kissel M. Modeling the Past: Archaeology, History, and Dynamic Networks. Berghahn Books; 2023. Available from: https://play.google.com/store/books/details?id=ziWBEAAAQBAJ

[42] Brughmans T, Peeples MA. Network Science in Archaeology. Cambridge University Press; 2023.

[43] Brandes U, Robins G, McCranie A, Wasserman S. What is Network Science? Netw Sci. 2013;1:1–15. doi: 10.1017/nws.2013.2

[44] Mills BJ, Clark JJ, Peeples MA, Haas WR Jr, Roberts JM Jr, Hill JB, et al. Transformation of social networks in the late pre-Hispanic US Southwest. Proc Natl Acad Sci U S A. 2013;110(15):5785–90. doi: 10.1073/pnas.1219966110 23530201 PMC3625298

[45] Scheidel W. The shape of the Roman world: modelling imperial connectivity. J Roman archaeol. 2014;27:7–32. doi: 10.1017/s1047759414001147

[46] Terrell JE. Human biogeography in the Solomon Islands. Fieldiana. 1977;68:1–47.

[47] Fraser D. The cutpoint index: A simple measure of point connectivity. Area. 1980;12:301–4.

[48] Peeples MA, Roberts JM. To binarize or not to binarize: Relational data and the construction of archaeological networks. J Archaeol Sci. 2013;40:3001–10. doi: 10.1016/j.jas.2013.03.014

[49] Borgatti SP. Centrality and network flow. Soc Netw. 2005;27:55–71. doi: 10.1016/j.socnet.2004.11.008

[50] Prignano L, Morer I, Diaz-Guilera A. Wiring the past: A network science perspective on the challenge of archeological similarity networks. Front Digit Humanit. 2017;4. doi: 10.3389/fdigh.2017.00013

[51] Gjesfjeld E. Network analysis of archaeological data from Hunter-Gatherers: Methodological problems and potential solutions. J Archaeol Method Theory. 2015;22(1):182–205. doi: 10.1007/s10816-014-9232-9

[52] Daems D, Coco E, Gillreath-Brown A, Kafetzaki D. The effects of time-averaging on archaeological networks. J Archaeol Method Theory. 2023;31(2):473–506. doi: 10.1007/s10816-023-09608-7

[53] Romanowska I, Wren CD, Crabtree SA. Agent-Based Modeling for Archaeology: Simulating the Complexity of Societies. Santa Fe: The Santa Fe Institute Press; 2021.

[54] Rogers JD, Cegielski WH. Opinion: Building a better past with the help of agent-based modeling. Proc Natl Acad Sci U S A. 2017;114(49):12841–4. doi: 10.1073/pnas.1718277114 29208733 PMC5724291

[55] Barton CM, Riel-Salvatore J. The formation of lithic assemblages. J Archaeol Sci. 2014;46:334–52. doi: 10.1016/j.jas.2014.03.031

[56] Brantingham PJ. A neutral model of stone raw material procurement. Am Antiq. 2003;68(3):487–509. doi: 10.2307/3557105

[57] Creanza N, Kolodny O, Feldman MW. Greater than the sum of its parts? Modelling population contact and interaction of cultural repertoires. J R Soc Interface. 2017;14(130):20170171. doi: 10.1098/rsif.2017.0171 28468920 PMC5454306

[58] White A. An abstract model showing that the spatial structure of social networks affects the outcomes of cultural transmission processes. J Artif Soc Soc Simul. 2013;16(3). doi: 10.18564/jasss.2219

[59] Barton CM, Riel-Salvatore J, Anderies JM, Popescu G. Modeling human ecodynamics and biocultural interactions in the Late Pleistocene of Western Eurasia. Hum Ecol. 2011;39(6):705–25. doi: 10.1007/s10745-011-9433-8

[60] Brughmans T, Poblome J. Roman bazaar or market economy? Explaining tableware distributions through computational modelling. Antiquity. 2016;90(350):393–408. doi: 10.15184/aqy.2016.35

[61] Crabtree SA. Inferring Ancestral Pueblo social networks from Simulation in the Central Mesa Verde. J Archaeol Method Theory. 2015;22(1):144–81. doi: 10.1007/s10816-014-9233-8

[62] Wilensky U. NetLogo. Evanston, Illinois: Center for Connected Learning; Computer-Based Modeling, Northwestern University; 1999.

[63] Drennan RD. Long‐distance transport costs in pre‐Hispanic Mesoamerica. Am Anthropol. 1984;86(1):105–12. doi: 10.1525/aa.1984.86.1.02a00100

[64] Romanowska I, Crabtree SA, Harris K, Davies B. Agent-based modeling for archaeologists: Part 1 of 3. Adv Archaeol Pract. 2019;7(2):178–84. doi: 10.1017/aap.2019.6

[65] Jennewein DM, Lee J, Kurtz C, Dizon W, Shaeffer I, Chapman A, et al. The Sol Supercomputer at Arizona State University. Practice and Experience in Advanced Research Computing. New York, NY, USA: Association for Computing Machinery; 2023. pp. 296–301. doi: 10.1145/3569951.3597573

[66] Peeples MA, Brughmans T. Online Companion to Network Science in Archaeology. 2023. Available from: https://archnetworks.ne

[67] Butts CT. sna: Tools for Social Network Analysis. 2023. Available from: https://CRAN.R-project.org/package=sna

[68] R Core Team. R: A Language and Environment for Statistical Computing. Vienna, Austria: R Foundation for Statistical Computing. 2024. Available: https://www.R-project.org/

[69] Krackardt D. QAP partialling as a test of spuriousness. Social Networks. 1987;9(2):171–86. doi: 10.1016/0378-8733(87)90012-8

[70] Handcock MS, Hunter DR, Butts CT, Goodreau SM, Krivitsky PN, Morris M. ergm: Fit, Simulate and Diagnose Exponential-Family Models for Networks. The Statnet Project; 2018. Available: https://CRAN.R-project.org/package=ergm 10.18637/jss.v024.i03 PMC2743438 19756229

[71] Hunter DR, Handcock MS, Butts CT, Goodreau SM, Morris M. ergm: A package to fit, simulate and diagnose exponential-family models for networks. J Stat Softw. 2008;24(3):nihpa54860. doi: 10.18637/jss.v024.i03 19756229 PMC2743438

[72] Amati V, Mol A, Shafie T, Hofman C, Brandes U. A framework for reconstructing archaeological networks using exponential random graph models. J Archaeol Method Theory. 2020;27(2):192–219. doi: 10.1007/s10816-019-09423-z 32508485 PMC7252583

[73] Brughmans T, Keay S, Earl G. Introducing exponential random graph models for visibility networks. J Archaeol Sci. 2014;49:442–54. doi: 10.1016/j.jas.2014.05.027

[74] Cegielski WH. Networks, Agent-Based Modeling, and Archaeology. In: Brughmans T, Mills BJ, Peeples MA, Munson J, editors. The Oxford Handbook of Archaeological Network Research. Oxford, England: Oxford University Press; 2024. pp. 280–293.

[75] Wang L-Y, Marwick B. A Bayesian networks approach to infer social changes from burials in northeastern Taiwan during the European colonization period. J Archaeol Sci. 2021;134:105471. doi: 10.1016/j.jas.2021.105471

[76] Gjesfjeld E, Colby Phillips S. Evaluating adaptive network strategies with geochemical sourcing data: A case study from the Kuril Islands. In: Knappett C, editor. Network analysis in archaeology: New approaches to regional interaction. Oxford University Press; 2013. pp. 281–305.

[77] Golitko M, Meierhoff J, Feinman GM, Williams PR. Complexities of collapse: the evidence of Maya obsidian as revealed by social network graphical analysis. Antiquity. 2012;86(332):507–23. doi: 10.1017/s0003598x00062906

[78] Hermann A, Forkel R, McAlister A, Cruickshank A, Golitko M, Kneebone B, et al. Pofatu, a curated and open-access database for geochemical sourcing of archaeological materials. Sci Data. 2020;7(1):141. doi: 10.1038/s41597-020-0485-8 32393758 PMC7214434

[79] Ladefoged TN, Gemmell C, McCoy M, Jorgensen A, Glover H, Stevenson C, et al. Social network analysis of obsidian artefacts and Māori interaction in northern Aotearoa New Zealand. PLoS One. 2019;14(3):e0212941. doi: 10.1371/journal.pone.0212941 30870446 PMC6417682

[80] Hastie TJ, Tibshirani RJ. Generalized Additive Models. London: Chapman & Hall; 1990.

[81] Wood SN. Generalized Additive Models: An Introduction with R. Chapman; Hall/CRC; 2017.

[82] Chudek M, Heller S, Birch S, Henrich J. Prestige-biased cultural learning: bystander’s differential attention to potential models influences children’s learning. Evol Hum Behav. 2012;33:46–56. doi: 10.1016/j.evolhumbehav.2011.05.005

[83] Henrich J, Boyd R. The evolution of conformist transmission and the emergence of between-group differences. Evol Hum Behav. 1998;19(4):215–41. doi: 10.1016/s1090-5138(98)00018-x

[84] Henrich J, Broesch J. On the nature of cultural transmission networks: Evidence from Fijian villages for adaptive learning biases. Philos Trans R Soc Lond B Biol Sci. 2011;366(1567):1139–48. doi: 10.1098/rstb.2010.0323 21357236 PMC3049092

