# Supplementary material for: Bridging material culture networks and social networks in archaeology
Source: PLoS One. 2026 Oct 16;21(10):e0358860. doi: 10.1371/journal.pone.0358860 (PMC13674714; doi:10.1371/journal.pone.0358860)
Supplement: Supplementary file 1 [file pone.0358860.s001.pdf]

ArchMatNet: Archaeological Material Networks

ODD: Overview, Design concepts, and Details

Cecilia Padilla-Iglesias, Claudine Gravel-Miguel, and Robert Bischoff *

2022-12-23

Contents

Purpose 3

Process overview and scheduling 3

Design concepts 4
- Basic principles 4
- Emergence 4
- Adaptation 4
- Objectives 5
- Learning 5
- Prediction 5
- Sensing 5
- Stochasticity 5
- Collectives 5
- Observation 5

Input data 6

Submodels 6
- Social activities 6
- Cultural transmission 6
- Cultural innovation 7
- Creating the archaeological record 8

Global 8
- Extensions 8
- Global Variables 8
- Agent Types 9
- Agent Properties 9
- Patch Variables 10
- Link Variables 10

Procedures 10
- setup 10
- setupPatches 12
- setupBandsAndTerritories 13
- setupCampsAndPeople 14

*authors listed alphabetically by first name–each contributed equally to the agent based model.

setupMasterTables 16
setupVisibility 16
go 16
changeActivity 19
aggregate 20
disperse 20
hunt 20
createAlliance 21
cleanUpAlliances 22
visit 23
calculatePrestige 24
tradeItems 24
dropItem 26
returnToCamp 27
updateCampInteractions 27
craftItem 28
migrate 29
recordCampPrestige 29
luckyLeap 30
createVariant 31
loseTrait 32
getNextNumber 33
getFitTrait 33
deconstructingTrait 34
getTraitNumber 35
nextLetter 35
traitVisibility 35
getMostlyVisibleTraits 36
getVariantsOfMainType 37
getLastVariant 37
updateVisibility 38
transmitCulture 38
transmitConformism 39
transmitPrestige 40
totalTraits 41
printMetrics 41
assemblageSize 42
interactionFreq 42
getBRBands 43
getBRCamps 43
calculateBR 44
listTraits 45
getTraitFreqTable 45
getFreqDistList 46
modularityBand 47
modularityCamp 47
resetTempLinksForModularity 48
networkMetric 48
setupJaccardDistBetweenTraits 49
setupJaccardDistBetweenObjects 49
jaccardDist 49
jaccardDistWeighted 50
PhiSTCamps 51
PhiSTBands 52

nTraits . . . 53
compileArtifactSimilarities . . . 53
compileCampInteractions . . . 54
updateAdjacencyTables . . . 54
fileNameBase . . . 54
exportResults . . . 55
exportFineTunedData . . . 56
getJaccardBand . . . 56
getJaccardCamp . . . 57
calculateJaccard . . . 57
adjCompare . . . 58
adjCorr . . . 58

GUI elements **59**

References cited **60**

Purpose

The purpose of the model is to investigate how different factors affect the ability of researchers to reconstruct prehistoric social networks from artifact stylistic similarities, as well as the overall diversity of cultural traits observed in archaeological assemblages. Given that cultural transmission and evolution are affected by multiple interacting phenomena, our model allows to simultaneously explore six sets of factors that may condition how social networks relate to shared culture between individuals and groups:

1. Factors relating to the structure of social groups
2. Factors relating to the cultural traits in question
3. Factors relating to individual learning strategies
4. Factors relating to the environment
5. Factors relating to the context in which different types of cultural traits are learned
6. Factors relating to the method used to reconstruct ancient social networks

Process overview and scheduling

At the beginning of each time-step, the model checks what type of environment is set. If it is only one large biome, it rolls the die to see if the environment will change. If it does, the new environment is a random value between 0 and 99. Patches get that new environment.

If the user wants highResolutionData, the model checks if it is time to export data (every 1000 time-steps). If it is the case, it calls procedures that compile data and the procedure that exports it to the correct CSV file.

People remove any superfluous alliances they may have accumulated in previous tick, using the cleanUpAlliances procedure.

People run the possibility to invent new types and new variants and create one pot and point each. A person may create a new pot and/or point each tick. Each new object is created based on a randomly selected trait from the person's trait list. Each object also records the tick when it was created for use in subsequent analyses.

The model then checks if it is time to aggregate (based on the aggregation-freq slider). If it is, it sets aggregation to T, and calls the aggregate procedure, which moves people to their respective aggregation camps.

While aggregating, the model keeps track of the aggregation's length. If it has reached the aggregationDuration limit, people go back to their own camp. When they are aggregating, people "visit" with all the people they

are aggregated with, which is recorded in the interactions' "visiting" counter, as well as the inter-camps campInteractions "visiting" counters. Aggregated people can go hunting with a certain probability.

When an aggregation is not occurring, people can do different activities, with a given probability. They can create a new alliance with someone from another camp. They can go visit an ally. They can go hunting.

When hunting, people gather other people from their band and go to a different location together. When the hunt occurs during an aggregation, the hunting site is within 15 patches of the aggregation site. When the hunt occurs at other times, the hunting site is simply within the band territory. On a hunt, the hunters calculate their pointPrestige, and have different probabilities to drop a point, trade points with one another, and transmit trait knowledge to one another. Hunters keep track of how many times they hunted together. This interaction is also recorded by the campInteractions counter between camps. When the hunt is over, everyone returns to their own camp.

When visiting, one person chooses one ally to go visit at random. They move to that ally's camp and visit with all the allies present there. The people who are involved in the visit keep track of how many times they visited one another, which is also recorded by the campInteractions visiting counter. The people who participate in the visit have different probabilities to drop one pot, drop one point, trade objects with one another, and transmit trait knowledge to one another. When those activities are done, the visitor returns to their own camp.

If recordPrestige is T, camps call the recordCampPrestige procedure. This procedure helps to follow the progression of mean and max prestige of the people of each camp, to see how those values change over time.

All people have a certain probability to call the loseTrait procedure, in which they forget one pot or point trait.

All people call the transmitCulture procedure to share knowledge of traits, and tradeItems to trade objects from their inventories.

Then, all people call the dropItem procedure, in which they have a certain probability to drop one pot or one point.

After the first 1000 ticks, people check if they want to migrate or not at every tick, based on a probability set by the user. When genderedMigration is T, only people with gender 1 roll the die and they have twice the probability to migrate as everyone does when genderedMigration is F. When migration occurs, the person who is migrating chooses another person to switch place with. When genderedMigration is T, only people of gender 1 can be selected. The two people switch home camps.

The model checks to see if it has reached the maximum number of ticks. If it has and the user wants to see the metrics, it computes and prints all the metrics to the Command Center, exports results as a CSV file if saveResults is T, and stops.

Design concepts

Basic principles

We built this model to allow testing assumptions that archaeologists have made about the possibility to reconstruct social networks from material culture, as well as improve our methods to reconstruct said networks.

Emergence

We expect styles to emerge over time within well-connected people who share the same traits and innovate on the shared known traits.

Adaptation

The people do not adapt to their environment or their context. Their behavior is constant throughout the run.

Objectives

One objective people have is to learn fitter traits (when learning via Prestige).

Learning

People regularly learn from the people around them. This is the base of the cultural transmission subprocedure. While the learning is not considered adaptation, people will learn the traits that are most popular at any given time, traits which change as innovations gain popularity or are lost.

Prediction

There is no prediction.

Sensing

People sense the environment onto which they hunt, and it affects their prestige. When meeting one another, people can sense which traits the others know. People also know who their allies are, and how often they have visited them. Camps and bands sense the knowledge of their inhabitants. None of the knowledge involve errors. Interaction Interactions make the most important part of the model. People hunt together and visit one another. When interacting, people can trade items or share knowledge of some traits.

Stochasticity

To keep the model general, we use stochasticity often. A lot of the activities are done with a certain probability, controlled by Interface sliders. Traded items are chosen at random amongst traders' inventories. When creating a new variant, the type to innovate upon is chosen randomly amongst the traits known by the innovator. Forgotten traits are also selected at random. This use of stochasticity is also because we do not know how often and how those activities were done in the past. Therefore, it is simpler to use probabilities to get a wider range of behaviors.

Collectives

People are grouped by camps, which are grouped by bands. There are three camps per band. People aggregate and hunt with the people from their band, therefore, their group affect who they will interact with the most, which in turn affects the similarity of their known traits and created objects.

Observation

The model can produce different types of outputs. It can calculate and print the following metrics (in the following names, X can be point or pot): 1) AssemblageSizeX: the sum of pots or points discarded at camps and bands; 2) interactionFreqY (where Y can be hunting, visiting, learning, and trading): the sum of all Y interactions between people; 3) brDistBandX: mean Brainerd Robinson distance between known traits, grouped by bands; 4) brDistCampX: mean Brainerd Robinson distance between known traits, grouped by camps; 5) meanPeoplePrestige: mean hunting prestige of all people; 6) modBandX: modularity metric which evaluates if there is more between-band links than within-band links, based on the Jaccard similarity of traits known by people; 7) modCampX: modularity metric which evaluates if there is more between-camp links than within-camp links, based on the Jaccard similarity of traits known by people; 8) networkMetricX: difference between the clustering coefficient of the network created by people who interacted with one another (hunting and learning) and the clustering coefficient of the network created by similarities in objects (point and pot); 9) PhiSTXBand: PhiST measure to see if variation in known traits is structured around bands; 10) PhiSTXCamp: PhiST measure to see if variation in known traits is structured around camps; 11) nTraitX: sum of the known traits at the end of the simulation (includes duplicates); and 12) elapsedTime: time elapsed since the start of the run.

In addition, the model can produce CSV outputs recording the parameter values, all the interactions between people, as well as the characteristics of all objects discarded at camp/band sites.

Input data

This model does not import external data.

Submodels

Social activities

People meet one another through hunting and visiting. Hunting is done with bandmates only, but visiting is done between allies, which can be of other bands, as hunter-gatherers in the ethnographic literature tend to travel further distances to visit acquaintances than to carry out subsistence activities (Hewlett et al. 1982; Weissner, 1977; Hill et al. 2014)

When someone initiates a hunt, they select a certain number of bandmates to bring with them. They all move to the hunting spot, which is either within the band territory, or within 15 patches of the aggregation site (when the hunt happens during an aggregation). All people taking part in one hunt update their and their camps' interaction "hunting" counter. Hunting people calculate the fitness of their known traits in the current environment, by calculating how many of their point traits were invented in the current environment. They then have a certain probability of dropping a point, trading items and/or transmit trait knowledge with a fellow hunter. After that, all hunters return to their own camp.

To visit an ally, a person has first to have allies. Allies are formed randomly and with a given probability at each tick. When forming an alliance, a person chooses another person at random. That ally can be of any camp other than ego's. When someone has more than the limit of allies allowed, they remove one. To select which alliance will go, the person uses weighted sampling, with the number of visits serving as the inverted weight (alliances of people who visit one another often will have less chances of being removed).

When visiting an ally, a person moves to the camp of one of their allies, choosing at random. If they have other allies in that camp, they all participate in the visit and they all update their and their camps' interaction "visiting" counter. They then have a certain probability of dropping a point and a pot, trading items and/or transmit trait knowledge with a fellow ally. After that, the visitor returns to their own camp. Item exchange

The tradeItems procedure controls exchange between people. It is only run if the person has a point or pot to give and has found a trading partner on the same patch who also has an object to trade in its inventory. This is done first for points, and then for pots. The trading partners for both objects do not need to be the same, but they can be. Both partners choose one item at random from their inventory, and swap them. The interaction link between the agents and the campInteraction links between their camps record that interaction.

Cultural transmission

Cultural transmission happens when the transmitCulture is called (in GO, hunt, and visit). This procedure identifies if the transmission will take place (based on probabilities) and the type of transmission. For pots, and then points, people roll the die to see if they will transmit information. When successful, they will call transmitPrestige with a probability set by the learningMethod slider. Every other time, they will call transmitConformism. These procedures return a set of new traits, following the rules detailed below.

After learning the new traits, people craft a new item (calling the craftItem procedure) and remove any duplicate traits they might have just learned. The transmitConformism [toolType] reporter takes as toolType either "pot" or "point" and is used to determine which attributes are copied.

1. Ego asks all people on the same patch (including itself) to select their traits of the given toolType.

2. If visibility changes how often traits are transmitted, everyone uses the getMostlyVisibleTraits reporter to get mostly visible traits from their list. High visibility traits are chosen for transmission 98% of the time. This value is arbitrary but generally fits expectations from a broad reading of the ethnographic literature.
3. Everyone pools their list together and the reporter reports the most common trait. Then, the interaction links between all people update their learning counter (+1).

The transmitPrestige [toolType] takes as toolType either "pot" or "point" and is used to determine which attributes are copied.

1. Ego identifies who on the same patch they will learn from. For points, they select the person with the highest pointPrestige. If potPrestige is T, they choose the person with the highest potPrestige, but if potPrestige is F, they choose someone at random. This person is now the "teacher."
2. The teacher adds its list of traits to a temporary global list.
3. If visibility affects transmission, the list is filtered through the getMostlyVisibleTraits reporter.
4. The reporter chooses and reports one of the traits in the teacher's list. The interaction links between the teacher and Ego updates its learning counter (+1).

The getMostlyVisibleTraits [traitList] reporter selects and reports mostly visible tools from the traitList given. There is some error as the visibility of a tool becomes its probability of being chosen.

1. This first separates the traitList into two lists. One that focuses on the main types, and one that focuses on types with variants. For the main types, it simply pairs this new list with a list of 2s of the same length, as all main tools have high visibility. For variants, it pairs the list with each variants' visibility obtained from the visibilityTable.
2. It then merges both paired lists and samples traits from that list, using their score as the probability to be chosen. The list of chosen traits is as long as the original list and includes replacements.
3. The reporter then removes any duplicates and reports this list.

Cultural innovation

Innovation can occur through the procedures luckyLeap (where a person creates a new main type) and createVariant (where a person creates a variant of a main type they know) (see Kolodny et al. 2015; Creanza et al. 2017 for a similar implementation of cultural dynamics in an ABM. This allows creating innovations that are dependent on other innovations, which is a key feature of cultural innovations. For example, the invention of a new snare hunting method may enable new kinds of game hunting and in turn the further invention of specialized tools for processing these novel foods. Such dependence in culture is what makes it cumulative over time.These procedures add the newly invented trait to a person's repertoire and to the master pot and point traits tables. Main types are coded as integers, and their variants are coded as letters.

The luckyLeap [nextTrait toolType] procedure is called by a person (ego) in Go if a random value is below the threshold set in pLuckyLeap (Interface). The value is multiplied by 2 for pots so that pots are twice less likely to see random innovations.

The nextTrait is the number of the next trait in the list of main tools to invent, calling getNextNumber to calculate it. The getNextNumber reporter takes the list of pot or point attributes ego has and reports the number following the number ego already knows. That number is converted as a string to be used by luckyLeap. The toolType can be either "pot" or "point" and is used to determine which traits are innovated.

1. Ego adds the nextTrait to its relevant trait list (point or pot).
2. It then removes any duplicates.
3. If this type is not in its relevant master table, it adds an entry for it.
4. If toolType is "point" and the traitEnvironment table does not have it already, ego adds an entry to that table with the key = trait, and the value = the environmentZone of the patch where ego stands.

The createVariant [toolType] procedure is called by a person in Go if a roll die is successful. It takes one main type from the agent's known traits and adds a new variant to it.

1. It first populates a temporary variable with the list of traits the agent knows for the given toolType.

2. It then chooses one trait at random from that list. It uses the getTraitNumber reporter to get the number of the trait (main type). It also identifies the last variant the agent owns of that type, using the getLastVariant reporter.
3. The procedure then checks if ego has reached the limit of possible variants ("o"). If they have not, they identify the next variant in the alphabet. This letter becomes the newVariation.
4. Ego merges the newVariation to the main type to create a new type with variant.
5. If toolType is "point" and the traitEnvironment table does not have it already, ego adds an entry to that table with the key = trait, and the value = the environmentZone of the patch where ego stands.

Creating the archaeological record

The dropItem procedure removes objects from a person's inventory based on the pointsBreakageRate or potsBreakageRate parameters, which are now set to 0.3. If the person dropping an object is hunting, the probability of discard is unchanged. If not hunting, the probability is divided by twenty to represent less likelihood of losing a point while engaged in other activities. This value was chosen to represent the probabilities of a point being discarded at a camp based on Claudine Gravel-Miguel et al. 2021. The potsBreakageRate does not vary. When dropping an item, a person removes the first item in its inventory (the oldest item made). If the person is on a camp or aggregation site, that site records the discarded object in its pot or point assemblage.

Global

Extensions

Description These are the extensions used in the model. Note that stats is not bundled and must be downloaded and placed in the extensions folder of the Netlogo app or in the same directory as the model. sr is commented out as it requires additional setup, but can be enabled by uncommenting.

```
extensions [
  profiler
  rnd
  nw
  table
  matrix
  stats
;  sr
]
```

Global Variables

Description These are the global variables defined for the model.

```
globals [
  inventorySizePoints ; size of point inventory allowed per person
  inventorySizePots ; size of pot inventory allowed per person
  groupColors ; list of colors possible for each group
  currentView ; determines whether environmental zones or territories are displayed
  aggregation  ; Monitor whether people are currently aggregating or not
  aggregationCounter  ; A counter to monitor the aggregation period
  hideLinksInteractions ; whether links should start out hidden or not
  randomWho ; random person to record activity for
  traitLetters ; for trait variations (new variants on main types)
```

```
  pointTraitsTable ; table that records all point traits created
  potTraitsTable ; table that records all pot traits created
  traitEnvironments ; table recording environment in which each point trait got created
↪  (the trait, not the object itself)
  visibilityTable ; table that records which letter traits are visible (2) or not (1)
  potBreakageRate; rate that pots break -- do not break while hunting
  pointsBreakageRate; rate that points break -- more likely to break while hunting
  campVisitingAdjacency ; record visits between their agent and other people
  campTradingAdjacency ; record trades between their agent and other people
  campHuntingAdjacency ; record hunts between their agent and other people
  campLearningAdjacency ; record learning between their agent and other people
  campPointAdjacency ; record the number of similar points between camps
  campPotAdjacency ; record the number of similar pots between camps
  fileHighRes ; file to record the high resolution dataset
]
```

Agent Types

Description Each agent type forms a nested hierarchy–bands contain camps which contain people.

```
breed [ bands band ] ; bands represent a higher-level grouping of people
breed [ camps camp ] ; camps represent a small group of people
breed [ people person ] ; people belong to a camp and its band

undirected-link-breed [ interactions interaction ] ; keep track of interactions between
↪  people
undirected-link-breed [ campInteractions campInteraction ] ; to record interactions
↪  between people, compiled by camps
undirected-link-breed [ tempLinks tempLink ] ; links created temporarily between
↪  individuals, camps, or bands to calculate different metrics
```

Agent Properties

Description These are the variables specific to each type of agent.

```
camps-own [
  potAssemblage ; pots deposited at camp
  pointAssemblage ; points deposited at camp
  ownedBy ; which band owns the camp
  otherCamps ; record camps that are not this one
  otherCampsMyband ; records camps that are in this camp's band
  meanPrestige ; record mean prestige per camp
  maxPrestige ; record prestige of most prestigious individual in a camp
  allPoints ; point traits known by the camp's people (with duplicates)
  allPots ; point traits known by the camp's people (with duplicates)
]

bands-own [
  potAssemblage ; pots deposited at band
  pointAssemblage ; points deposited at band
  allPoints ; point traits known by the band's people (with duplicates)
  allPots ; pot traits known by the band's people (with duplicates)
```

```
]

people-own [
  activity ; variable to hold the activity the agent is engaged in
  originalHome ; location of originalHome camp
  homeBand ; origin band for each person
  homeCamp ; camp the person lives at
  pointTraits ; point traits known (can be used to create a point or be transfered to
↪  someone else)
  potTraits ; pot traits known (can be used to create a pot or be transfered to someone
↪  else)
  pointInventory ; points the person possesses
  potInventory ; pots the person possesses
  pointPrestige ; value influenced by the traits possessed
  potPrestige ; value given at random
  huntResults ; list of last 10 hunting trip success rates
  myAllies ; people with whom person has an alliance
  gender ; 0 or 1 to represent two different genders (for gendered migration only)
]
```

Patch Variables

Description These variables are specific to patches.

```
patches-own [
  environmentZone ; type of environment for patch
  pOwnedBy ; which band owns the patch
]
```

Link Variables

Description These variables are specific to links.

```
links-own [
  ally?  ; switch to determine whether links will be "ON" or "OFF"
  lastActive ; counter to record the last tick that the link was active if switched off
  jaccard ; record the Jaccard distance

  ; keep track of every time people interact for the following categories
  learning
  visiting
  hunting
  trading
]
```

Procedures

setup

Description Setup the model.

```
to setup

;  sr:setup ; setup R extension -- comment out if not using adjcorr reporter

  ; reset everything
  reset-timer ; track time
  stop-inspecting-dead-agents ; remove any open turtle windows for now dead turtles
  clear-all ; reset everything but time
  reset-ticks ; reset time

  ; set a random seed for each run if option is true - this allows the model to record
↪  the seed for reproducibility
  if randomizeSeed [ set randomSeed random 10000 ]
  random-seed randomSeed ; set random seed

  ; track one person's activity
  if recordPerson [
    if file-exists? "results/outputActivity.csv" [ file-delete
↪  "results/outputActivity.csv" ] ; overwrites the file if it already exists
    file-open "results/outputActivity.csv"
    file-print "activity, tick"
    file-close
    set randomWho nofBands * 3 + nofBands + campPopulation
    show ( word "tracked person is " randomWho ) ; prints who is followed in the command
↪  center
  ]

  ; create a new file to log into data at every 1000 tick if wanted
  if HighResolutionData [
    set fileHighRes (word "results/outputHighRes-" filenamebase ".csv")
    file-open fileHighRes
  ]

  ; set some defaults values or parameter settings
  set potBreakageRate 0.3
  set pointsBreakageRate 0.3
  set hideLinksInteractions true ; by default, links are not visible (for World clarity)
  set aggregation false ; the simulation starts with people in their respective camps
  set inventorySizePoints 10 ; people can only carry up to 10 points
  set inventorySizePots 10 ; people can only carry up to 10 pots
  set traitLetters ( list "a" "b" "c" "d" "e" "f" "g" "h" "i" "j" "k" "l" "m" "n" "o" ) ;
↪  creates the possible set of new variant letters

  ; procedures to create master tables, patches with environments, bands, camps, and
↪  people
  setupMasterTables
  if visibility [ setupVisibility ] ; assign visibility to specific variants if it
↪  affects transmission
  setupPatches
  setupBandsAndTerritories
  setupCampsAndPeople
```

```
  ; create empty matrices (or adjacency tables) that log:
  set campVisitingAdjacency matrix:make-constant count camps count camps 0 ; visiting
↪  interactions between camps at each wanted iteration
  set campTradingAdjacency matrix:make-constant count camps count camps 0 ; trading
↪  interactions between camps at each wanted iteration
  set campHuntingAdjacency matrix:make-constant count camps count camps 0 ; hunting
↪  interactions between camps at each wanted iteration
  set campLearningAdjacency matrix:make-constant count camps count camps 0 ; learning
↪  interactions between camps at each wanted iteration
  set campPointAdjacency matrix:make-constant count camps count camps 0 ; similarities in
↪  points between camps at each wanted iteration
  set campPotAdjacency matrix:make-constant count camps count camps 0 ; similarities in
↪  pots between camps at each wanted iteration

end
```

setupPatches

Description Procedure to create patch environments (1 or 3).

```
to setupPatches

  ; define initial viewpoint as environmental zones
  set currentView "zone"

  ; if chooser gradualEnvChange on - homogenous environmentZone that changes over time
  ifelse gradualEnvChange [
    ask patches [ set environmentZone 67 ] ; all patches get the same environmentZone
  ][
    let pZones [ 47 67 37 ] ; colors for patches

    ; each pZone finds an unassigned patch, make it their own and create a buffer around
↪  it
    foreach pZones [
      a -> ask one-of patches with [ environmentZone = 0 ][
        set environmentZone a
        set pOwnedBy a ; borrowing this variable to speed up the code as it is not set
↪  yet
      ]
    ]

    ; sample patches to speed up code
    let assignedPatches patches with [ pOwnedBy != 0 ] ; only the 3 patches that have an
↪  environment (will be the center of each environment)

    ; Each patch without an environmentZone takes the environmentZone of the closest
↪  patch with one
    ask patches with [ environmentZone = 0 ][
      set environmentZone [ environmentZone ] of min-one-of assignedPatches [ distance
↪  myself ]
    ]
```

```
  ]

  ask patches [ set pcolor environmentZone ]

end
```

setupBandsAndTerritories

Description Procedure to create bands and their territories. Each band is separated by a minimum distance.

```
to setupBandsAndTerritories

  ; create invisible bands that will define the extent of the band's territory
  ; important - this code will separate each band by a specific radius, if the
  ; radius is too high then the code will print an error

  let radiusBand 15 ; radius for min distance between band centers
  let nBands ( count bands ) ; number of bands (update through the while loop)
  set groupColors [ 14 24 34 44 54 64 74 84 94 104 114 124 134 ] ; define colors for
↪  bands
  let tmpColor groupColors ; list of colors the bands take from (and remove the color
↪  they took so that each band has a unique color)
  let counter 0 ; to break the while loop in certain circumstances

  while [( nBands < nOfBands ) and ( counter < 10 )][
    let potentialPatches 0
    create-bands 1 [
      set hidden? false
      set shape "circle"
      set size 3
      set label who
      set label-color white
      ; set color from list but make sure it is unique
      set color ( item 0 tmpColor )
      set tmpColor ( remove-item 0 tmpColor )
      move-to one-of patches

      ; if there are other bands too close, the band will move somewhere else
      if any? other bands in-radius radiusBand [
        set potentialPatches patches with [ not any? bands in-radius radiusBand ]
        ifelse any? potentialPatches [
          move-to one-of potentialPatches
        ][
          set counter 10 ; to kill the while loop
          die
        ]
      ]
    ]
    set nBands ( count bands )
    set counter counter + 1
  ]
```

```
  ; warning message if radius is too high and not all bands can be placed
  if nBands < nOfBands [ print "did not succeed in placing all bands" ]

  ; define default variable for pOwnedBy
  ask patches [ set pOwnedBy 9999 ]

  ; setup bands as aggregation sites (and their inventories)
  ask bands [
    set pointAssemblage []
    set potAssemblage []
    set size 10
    set label ""
    set hidden? false
    set shape "campsite"
    ask patches in-radius radiusBand [ set pOwnedBy [ who ] of myself ] ; set band
↪  territory
  ]

  let tmpPatches up-to-n-of 100 patches with [ pOwnedBy != 9999 ]

  ask patches with [ pOwnedBy = 9999 ][
    set pOwnedBy [ pOwnedBy ] of min-one-of tmpPatches [ distance myself ]
  ]

end
```

setupCampsAndPeople

Description Procedure to set up camps and their people.

```
to setupCampsAndPeople

  ; create camp sites within band territory
  ; follows the same rules above by not letting camps be too close
  ; but in this case all the camps are created first and then moved

  let nOfCamps 3 ; number of camps per band
  let radiusCamp 10 ; radius for camps to be spread out

  ask bands [
    hatch-camps nOfCamps [
      ; initialize lists to hold deposited items
      set pointAssemblage []
      set potAssemblage []
      set size 6
      set label ""
      set hidden? false
      set shape "campsite"
      set ownedBy [ who ] of myself
      set meanPrestige [] ; list of mean prestige of the camp per tick
      set maxPrestige [] ; list of max prestige of the camp per tick
```

```
    ]
  ]

  ; set additional variables after all camps are created
  ask camps [
    set otherCamps other camps
    set otherCampsMyband other camps with [ ownedBy = [ ownedBy ] of myself ]
    create-campInteractions-with otherCamps [
      set hidden? true
    ]
  ]

  ; move camps to their location
  ask bands [
    let tmpPatches patches with [ pOwnedBy = [ who ] of myself ] ; identifies the band's
↪  territory
    ask camps with [ ownedBy = [ who ] of myself ][ move-to one-of tmpPatches ] ; asks
↪  their camp to move to their territory
  ]

  ; if the camp is too close to someone else, they try to move
  ask camps [
    if any? other camps in-radius radiusCamp [
      let potentialPatches patches with [ pOwnedBy = [ OwnedBy ] of myself and not any?
↪  camps in-radius radiusCamp ]
      ifelse any? potentialPatches [
        move-to one-of potentialPatches
      ][
        show ( word [ who ] of one-of other camps in-radius radiusCamp " is too close" )
      ]
    ]
  ]

  ; create user specified number of people per camp
  ask camps [
    hatch-people campPopulation [
      set shape "person" ; make it look like a person
      set size 2 ; change size
      set homeBand [ pOwnedBy ] of patch-here ; set home band
      set homeCamp myself ; set home camp
      set originalhome [ who ] of myself; set original home camp
      set pointInventory [] ; create empty list to hold point inventory
      set potInventory [] ; create empty list to hold pot inventory
      set pointTraits [] ; create empty list to hold point traits
      set potTraits [] ; create empty list to hold pot traits
      set huntResults [] ; list of hunting results
      set myAllies ( turtle-set ) ; set this as turtle-set that will be filled later
      set potPrestige precision ( random-normal 10 5 ) 2 ; each person gets a value from
↪  random distribution with mean 10 and standard deviation 5 (arbitrary)

      ; get its first known trait
      let minTrait 1 ; set the min trait to create objects from (by default is 1)
      if uniqueTraits [ set minTrait [ who ] of homeCamp * 10 ] ; but if we want
↪  different values per camp, this changes it trait set for each camp
```

```
      luckyLeap ( word minTrait ) "point" ; this also creates one point at the beginning
      luckyLeap ( word minTrait ) "pot" ; this does NOT create a pot at the beginning
    ]
    ; each camp asks 1/2 of its population to set their gender to 1
```

setupMasterTables

Description Observer procedure. Creates 3 tables, one for point traits, one for pot traits, and one for points' environment (in which environment aare they fit?).

```
to setupMasterTables

  set pointTraitsTable table:make  ; Table that will take on the learned values of main
↪  traits
  table:put pointTraitsTable "mainType" "variations" ; For easier visualization of which
↪  values are what

  set potTraitsTable table:make  ; Table that will take on the learned values of main
↪  traits
  table:put potTraitsTable "mainType" "variations" ; For easier visualization of which
↪  values are what

  set traitEnvironments table:make  ; Table that will take on the learned values of main
↪  traits
  table:put traitEnvironments "trait" "environment" ; For easier visualization of which
↪  values are what

end
```

setupVisibility

Description Observer procedure. Creates visibility table.

```
to setupVisibility

  set visibilityTable table:make

end
```

go

Description This is the main procedure that calls the others. In this order: the visibility table is updated; the environment is changed (if necessary), the adjacency tables are updated, alliances are cleaned, innovations are made, people craft new object, aggregation occurs (if applicable), people do activities, prestige gets calculated, Traits are lost, traits and items are transmitted, objects are dropped, migration occurs (if applicable). Then, if the run has reached its end, metrics are outputted (if applicable).

```
to go

  ; update visibility table
  if visibility [ updateVisibility ]
```

```
  ; change the environmentZone
  if gradualEnvChange and random-float 100 < pEnvChange [ ; if the environment is set to
↪  change over time and the roll die is successfull...
    print ( word "Climate change at tick: " ticks )
    let newZone random 100 ; random number (integer)
    ask patches [ set environmentZone newZone ]
  ]

  ; Update adjacency tables when in need of high resolution data (need to figure out
↪  timing, right now, it updates every 1000 ticks)
  if HighResolutionData and ticks > 0 and remainder ticks 1000 = 0 [ ; this is active
↪  only if we need it
    compileArtifactSimilarities
    compileCampInteractions
    exportFineTunedData
    ; clear camp interaction links and camp assemblages
    ask campInteractions [
      set learning 0
      set visiting 0
      set hunting 0
      set trading 0
    ]
    ask camps [
      set potAssemblage []
      set pointAssemblage []
    ]
  ]

  ; people remove alliancess if they are over the limit, before doing anything else
  ask people [ cleanUpAlliances ]

  ; Innovate at very low probability
  ask people [
    if random-float 1000 < pNewVariant [ createVariant "point" ] ; if die is successful,
↪  create a new point variant
    if random-float 1000 < pNewVariant [ createVariant "pot"] ; if die is successful,
↪  create a new pot variant
    if random-float 1000 < pLuckyLeap [ luckyLeap ( getNextNumber "point" ) "point" ] ;
↪  if die is successful, create a new point type
    if random-float 1000 < pLuckyLeap * 2 [ luckyLeap ( getNextNumber "pot" ) "pot" ] ;
↪  if die is successful, create a new pot type (not as often as points)
  ]

  ; produce new items if needed
  ask people [
    craftItem "point" true ; the "true" calls a probability within the craftItem
↪  procedure, so that they create new objects at a certain rate
    craftItem "pot" true ; the "true" calls a probability within the craftItem procedure,
↪  so that they create new objects at a certain rate
  ]

  ; control aggregation
```

```
  if ticks > 0 and remainder ticks aggregationFreq = 0 [ ; If it is time to aggregate
↪  then aggregate
    set aggregation true
    aggregate
  ]

  ifelse aggregation = true [
    ifelse aggregationCounter = aggregationDuration [  ; If the aggregation duration
↪  period has passed then disperse
      set aggregationCounter 0
      disperse
    ][
      set aggregationCounter ( aggregationCounter + 1 ) ; if they are still aggregating,
↪  update the counter and push people to participate in some activities (hunting only,
↪  as they are already visiting)
      ask people [
        changeActivity "aggregating"
        if random-float 100 < pHunting [ ; random decision to hunt based on slider
↪  specified odds
          changeActivity "hunting"
          hunt  ; procedure to create travel groups and destinations
        ]
      ]
    ]
  ][

    ; trading, hunting and visiting only if not aggregated
    ask people [
      if random-float 100 < pNewAlliance [ createAlliance ] ; procedure to create
↪  alliances who can be outside one's band
    ]

    ; do activities (the same person can both go hunting and go visiting in the same tick
    ask people [
      if random-float 100 < pHunting [ ; random decision to hunt based on slider
↪  specified odds
        changeActivity "hunting"
        hunt  ; procedure to create travel groups and destinations
      ]
      if random-float 100 < pVisiting and any? myAllies [ ; you cannot go visit anyone if
↪  you dont have any allies
        changeActivity "visiting"
        visit  ; procedure to create travel groups and destinations
      ]
    ]
  ]

  if recordPrestige [ ; if recordPrestige switch is on
    ask camps [ recordCampPrestige ] ; camps record the prestige variables from their
↪  people (mean and max)
  ]

  ; give each person chance to lose a trait (forget what they know) - equal chance to
↪  lose pot or point trait
```

```
  ask people [
    if random-float 100 < pLoss [
      ifelse random 2 = 0 [ ; roll the dice to see if they will lose a point or pot trait
↪  that time
        set pointTraits ( loseTrait pointTraits )
      ][
        set potTraits ( loseTrait potTraits )
      ]
    ]
  ]

  ; cultural transmission of known traits and exchange of objects
  ask people [
    transmitCulture
    tradeItems
  ]

  ; drop object from inventory at random
  ask people [
    dropItem "point" true
    dropItem "pot" true
  ]

  if ticks >= 1000 [
    ask people [
      ; Migration (or exogamy) procedure
      ifelse genderedMigration = TRUE [ ; when genderedMigration occurs, only people with
↪  gender 1 will move
```

changeActivity

Parameters Description needs to be a string identifying the new activity. It can be “hunting”, “learning”, “trading”, or “visiting”.

Description Change the activity of the person and if recordPerson is true then records the activity.

```
to changeActivity [ description ]

  set activity description

  if recordPerson and who = randomWho [ ; if tracking someone, open the file, write the
↪  new activity with timestamp, and close the file
    file-open "results/outputActivity.csv"
    file-print ( word ( activity ) "," ( ticks ))
    file-close

    if debug [ show ( word activity " on tick " ticks )] ; if debugging, show the new
↪  activity and timestamp
  ]

end
```

aggregate

Description Observer procedure. Tells people to go to their band's spot, which serves as the aggregation camp. All people in the same camp add 1 visiting to their counter. People can then create alliance and exchange items with people in the same camp.

```
to aggregate

  if debug [ print "aggregate" ] ; for debugging

  ; people move to their band
  ask people [ move-to band homeBand ]

  ask interactions with [ link-length = 0 ][ ; this focuses only on links between people
↪  who are in the same aggregation camp.
    set visiting visiting + 1 ; this assumes that everyone aggregating with one another
↪  has some sort of social contact (counted only once, not every month of aggregation)
    updateCampInteractions "visiting" end1 end2 ; updates the camp counter
```

disperse

Description Observer procedure. Called when the aggregation is over. Tells people to return to their home camp.

```
to disperse

  if debug [ print "disperse" ] ; for debugging

  set aggregation false ; reset the variable
  set aggregationCounter 0 ; restart the counter
  ask people [ returnToCamp ] ; people go back to their camp

end
```

hunt

Description People procedure. People go hunting with band mates.

```
to hunt

  let party ( turtle-set self ) ; create turtle-set of hunting or visiting party

  let activityLocation 0
  let availablePeople 0

  ; sets the activity location (when aggregating, it is nearby, when not aggregating, it
↪  is simply within the band territory)
  set activityLocation ifelse-value aggregation [ one-of patches in-radius 15 ][ one-of
↪  patches with [ pOwnedBy = [ homeBand ] of myself ]]
  move-to activityLocation ; move to the set location

  ; identify the hunting party (always band mates)
```

```
  set availablePeople ( turtle-set other people with [ homeBand = [ homeBand ] of myself
↪  ])
  ask up-to-n-of huntingGroupSize availablePeople [ ; tell a specific number of those
↪  people to join the hunt
    changeActivity "hunting"
    set party ( turtle-set party self )
    move-to activityLocation

    ; add to counters for activity (each member of the party does it once with the person
↪  starting the hunt, so everythin counts only once).
    updateCampInteractions "hunting" self myself ; this updates the counter of the camps
    ask interaction [ who ] of self [ who ] of myself [ set hunting hunting + 1 ] ;
↪  update the people counter
  ]

  ask party [
    calculatePrestige ; this assumes that the hunt occurred and everyone evaluated how
↪  well their knowledge fit the current environment
    dropItem "point" true ; this calls the dropItem procedure to see if the people lose
↪  one of their points ("true" means that it will happen with a certain probability)
    tradeItems ; people hunting together have the opportunity to trade points with one
↪  another
    transmitCulture ; people hunting together also share knowledge with one another
  ]

  ; when all members of the party have had the chance to do all activities, they all
↪  return home
  ask party [ returnToCamp ] ; this needs to be separated so that people don't leave
↪  before others have had the time to share with them

end
```

createAlliance

Description People procedure. People create a new alliance. When aggregating, they choose someone new (who is not already their ally) in the same camp. At other times, they choose someone new from another camp, favoring people from nearesst camps.

```
to createAlliance

  if debug [ print "createAlliance" ] ; for debugging

  let newAlly 0 ; temp

  changeActivity "making allies"

  ifelse aggregation = true [
    if any? other people-here [ ; When aggregating, they choose someone in the same band
↪  who is not already their ally, who can get a new ally, and who is not from the same
↪  camp
      set newAlly ( one-of other people-here with [ not member? self myAllies and count
↪  myAllies < maximumAlliesN and homeCamp != [ homeCamp ] of myself ])
```

```
    ]
  ][ ; when not aggregating, they can form an alliance with someone outside their band
↪  (they favor people from nearby camps, though)
    let allOtherCamps []
    let distAllOtherCamps []
    ask [ otherCamps ] of homeCamp [ ; this fills in the two temporary lists above (the
↪  camps are in the same order)
      set allOtherCamps lput self allOtherCamps ; list of camps other than homeCamp
      set distAllotherCamps lput precision ( distance [ homeCamp ] of myself ) 0
↪  distAllOtherCamps ; list of their distance from homeCamp (without decimals)
    ]

    let maxDist max distAllOtherCamps ; the furthest camp
    set distAllOtherCamps ( map [ x -> abs( x - maxDist )] distAllOtherCamps ) ; inverts
↪  the distance so that the closest camp has the highest probability
    let pairs ( map list allOtherCamps distAllOtherCamps ) ; Pairs the camps with their
↪  probabilities

    let selectedCamp first rnd:weighted-one-of-list pairs [[ p ] -> last p ] ; chooses
↪  one camp at random, but favoring closest ones
    set newAlly one-of people with [ homeCamp = selectedCamp and not member? self
↪  myAllies and count myAllies < maximumAlliesN ] ; select one person from that camp who
↪  is not yet its ally
  ]

  if is-turtle? newAlly [ ; in case no newAlly was found
    ask interaction [ who ] of self [ who ] of newAlly [ set ally? true ] ; activate the
↪  alliance

    ; reset the agentset to only allies from active links and tell the new ally to reset
↪  theirs as well
    set myAllies ( turtle-set [ other-end ] of my-interactions with [ ally? ])
```

cleanUpAlliances

Description People procedure. People count the number of allies they have and drop one or more if they have more than the limit. Allies with strong ties have less chance to get dropped than allies with weak ties.

```
to cleanUpAlliances ; to make sure no one ever has more allies than allowed

  if debug [ print "cleanUpAlliances" ] ; for debugging

  ; remove alliances with probability inversely proportional to the number of times
↪  you've visited that ally
  if count myAllies > maximumAlliesN [ ; if you have more allies than maximum
    let allyVisitsList ([ visiting ] of my-interactions with [ ally? ]) ; create a
↪  temporary variable with the list of all links' visiting counters
    set allyVisitsList ( map [ x -> x + 1 ] allyVisitsList ) ; add one to avoid dividing
↪  by zero when no visit have yet occurred
    let sumVisits sum allyVisitsList ; calculate the sum of the number of visits to other
↪  people
    let allyProbList allyVisitsList ; create a copy list that will take in the calculated
↪  probabilities of each visit value
```

```
  foreach range length allyProbList [ itemN  -> ; itemN takes the value of each item in
↪  the allyProbList list
    let currentVisitN item itemN allyProbList ; Reports the value of each link (number
↪  if visits)
    set allyProbList replace-item itemN allyProbList ( 1 / ( currentVisitN / sumVisits
↪  )) ; the probability that the link will be lost will be inversely related to network
↪  counter
  ]

  let pairs ( map list allyVisitsList allyProbList ) ; Pairs the link counters with
↪  their 0-1 probabilities
  let chosenVisitN rnd:weighted-one-of-list pairs [[ p ] -> last p ] ;
↪  rnd:weighted-one-of-list reports a random agent from the 'pairs' list, taking their
↪  probability (p) into account
  let chosenLink one-of interactions with [ visiting = ( first chosenVisitN ) - 1 ] ;
↪  The link with the chosen cost (see above) is used as an alliance.
  ask chosenLink [
    set ally? false ; link becomes inactive (alliance lost)
    set lastActive ticks
  ]
  set myAllies ( turtle-set [ other-end ] of my-interactions with [ ally? ]) ; reset
↪  agentset to only active links after making some inactive
```

visit

Description People procedure. People go visit one of their allies.

```
to visit

 let party ( turtle-set self ) ; will take on all people participating in the activity
 move-to one-of myAllies ; moving to the ally should be the same as moving to its camp.
                         ; as we are choosing amongst allies, a camp with multiple
↪  allies will have more chances to be chosen (same as when choosing amongst camps)

 let alliesHere people-here with [ member? myself myAllies ] ; identifies all the allies
↪  within one camp
 ask alliesHere [ ; visit only allies in destination camp
   changeActivity "visiting"
   set party ( turtle-set party self ) ; add themselves to the party (to do activities
↪  later on)

   ; add to counters for activity (each person visited does it once with the person
↪  visiting, so everythin counts only once).
   updateCampInteractions "visiting" self myself ; this updates the counter of the camps
   ask interaction [ who ] of self [ who ] of myself [ set visiting visiting + 1 ] ;
↪  this is done only by the allies, so it counts only once.
 ]

 ask party [ ; then everyone does a few activities
   dropItem "point" true ; they will drop a point with a certain probability
   dropItem "pot" true ; they will drop a pot with a certain probability
```

```
    tradeItems ; they will drop a pot with a certain probability
    transmitCulture
  ]

  returnToCamp ; this is separate from the "ask alliesHere" because allies stay in their
↪  camp. Only ego goes home.

end
```

calculatePrestige

Description People procedure. People take their inventory of points and calculate how many of them are fit in the current environment. This gets added to their huntResults variable. They take the average of the last 10 of those values as their prestige.

```
to calculatePrestige

  if debug [ print "calculatePrestige" ] ; for debugging

  if length pointInventory > 0 [ ; prestige is only calculated on the points in the
↪  inventory
    let onlyPoints ( map [ x -> last x ] pointInventory ) ; points in pointInventory have
↪  the tick and then the object. This filters out the tick to keep only the point
    let toolList ( map [ x -> table:get traitEnvironments x ] onlyPoints ) ; this
↪  extracts the environment in which the trait of each object in inventory was invented
    let myZone [ environmentZone ] of patch-here ; variable to determine environmental
↪  zone of location
    let usefulToolList filter [ i -> i = myZone ] toolList ; keeps only the points'
↪  environments that fit the current environment
    let fitness length usefulToolList / length toolList ; the ratio of points the person
↪  has that is good for the current environment

    set huntResults lput fitness huntResults ; add the latest fitness to the list of
↪  huntResults
    if length huntResults > 10 [ set  huntResults remove-item 0 huntResults ] ; this list
↪  can only have the latest 10 hunts, so the oldest 11st gets dropped, when appropriate
    set pointPrestige mean huntResults ; pointPrestige is the average of the last 10
↪  hunts' fitness
  ]

end
```

tradeItems

Description People procedure. Two people in the same place exchange one of their point and/or pot with one another. When they do so, they add 1 to their "trading" counter.

```
to tradeItems

  if debug [ print "tradeItems" ] ; for debugging

  let egoCurrentActivity activity ; so they can go back to their activity after the
↪  exchange
```

```
  let partnerCurrentActivity 0 ; for the partners to remember what they were doing too
  let theirGift 0 ; hold their present

  ;; POT ;;

  if random-float 100 < pTrading and activity != "hunting" and length potInventory > 0 [
↪   ; pot trading cannot occur during a hunt or if you have nothing to trade
    let tradingPartners other people-here with [ length potInventory > 0 ] ; find other
↪   people who also have pots to trade
    if any? tradingPartners [ ; the trade occurs only if they found someone who had
↪   something to trade
      changeActivity "trading pots"
      let myGift one-of potInventory ; choose gift
      let posGift position myGift potInventory ; identify the position of the item to be
↪   given
      set potInventory remove-item posGift potInventory ; remove that specific object
↪   from the inventory
      ask one-of tradingPartners [ ; exchange with one person
        set partnerCurrentActivity activity ; identify the activity the partner was doing
        changeActivity "trading pots" ; change activity (for debugging, mostly)
        set theirGift one-of potInventory ; they choose one pot to trade
        set posGift position theirGift potInventory ; identify the position of the item
↪   to be given
        set potInventory remove-item posGift potInventory ; remove that specific object
↪   from the inventory
        set potInventory lput myGift potInventory ; then add the new item
        changeActivity partnerCurrentActivity ; reset to previous activity

        ; update the interaction counters for camps and people links
        updateCampInteractions "trading" self myself ; update the counter of the camps
        ask interaction [ who ] of self [ who ] of myself [ set trading trading + 1 ] ;
↪   update the people counter
      ]
      set potInventory lput theirGift potInventory ; add the partner's gift to inventory
      changeActivity egoCurrentActivity ; resets to previous activity
    ]
  ]

  ;; POINT ;;

  if random-float 100 < pTrading and length pointInventory > 0 [ ; point trading can only
↪   happen if there are points to trade (which should always be the case)
    let tradingPartners other people-here with [ length pointInventory > 0 ] ; find other
↪   people who also have points to trade
    if any? tradingPartners [ ; the trade occurs only if they found someone on the same
↪   spot who had something to trade
      changeActivity "trading points"
      let myGift one-of pointInventory ; choose gift
      let posGift position myGift pointInventory ; identify the position of the item to
↪   give
      set pointInventory remove-item posGift pointInventory ; remove that specific object
↪   from the inventory
      ask one-of tradingPartners [ ; exchange with one person
```

```
        set partnerCurrentActivity activity ; identify the activity the partner was doing
        changeActivity "trading points" ; change activity (for debugging, mostly)
        set theirGift one-of pointInventory ; they choose one point to trade
        set posGift position theirGift pointInventory ; identify the position of the item
↪  to give
        set pointInventory remove-item posGift pointInventory ; remove that specific
↪  object from the inventory
        set pointInventory lput myGift pointInventory ; then add the new item
        changeActivity partnerCurrentActivity ; reset to previous activity

        ; update the interaction counters for camps and people links
        updateCampInteractions "trading" self myself ; update the counter of the camps
        ask interaction [ who ] of self [ who ] of myself [ set trading trading + 1 ] ;
↪  update the people counter
      ]
      set pointInventory lput theirGift pointInventory
      changeActivity egoCurrentActivity ; resets to previous activity
    ]
  ]

end
```

dropItem

Parameters itemType can be "point" or "pot" so it can be called individually. prob? is a boolean used to increase flexibility of the procedure (so it can be called by breaking the object as well as if they are over their inventory limit).

Description People procedure. People drop one itemType with a probability set by the object's breakageRate (if prob? is FALSE) or automatically if they are over their hand limit (prob? TRUE). Hunters are much more likely to drop a point when hunting. If it was the last point they had, they create a new one. If the drop occurs at a camp, that camp records the object's characteristics.

```
to dropItem [ itemType prob? ]

  if debug [ print "drop-item" ] ; for debugging

  ifelse itemType = "point" [

    ;; POINT;;
    ; the threshold for breakage is lower if the agent is hunting
    let threshold ifelse-value activity = "hunting" [ pointsBreakageRate ][
↪  pointsBreakageRate / 20 ]

    ; the first part of the line below is if dropItem is called during a hunt or GO, the
↪  second is if it is called because the person has reached its hand limit
    if ( prob? and random-float 100 < threshold ) or ( not prob? ) [
      if ( length pointInventory > 0 )[ ; only drop if an item exists
        changeActivity "dropping point"
        let site ( turtle-set camps-here bands-here ) ; identifies if the person is at a
↪  camp or band
```

```
      ; if there is a site here, it records the dropped point (the oldest of the
↪  inventory)
      ask up-to-n-of 1 site [ set pointAssemblage lput ( item 0 [ pointInventory ] of
↪  myself ) pointAssemblage ]
      set pointInventory remove-item 0 pointInventory ; remove the dropped item from
↪  inventory

      ; if the inventory is empty, create a new point (hunters always need to have one
↪  point in their inventory)
      if ( length pointInventory = 0 )[ set pointInventory lput ( list ticks one-of
↪  pointTraits ) pointInventory ]
     ]
   ]
 ][

   ;;POT ;;
   ; same procedure as for points, except activity does not affect breakage rate
   if ( prob? and random-float 100 < potBreakageRate ) or ( not prob? )[
     if ( length potInventory > 0 )[ ; only drop if an item exists
       changeActivity "dropping pot"
       let site ( turtle-set camps-here bands-here ) ; identifies if the person is at a
↪  camp or band

       ; if there is a site here, it records the dropped pot (the oldest of the
↪  inventory)
       ask up-to-n-of 1 site [ set potAssemblage lput ( item 0 [ potInventory ] of
↪  myself ) potAssemblage ]
       set potInventory remove-item 0 potInventory ; remove that pot from the inventory
     ]
   ]
 ]

end
```

returnToCamp

Description People procedure. Agents move back to home camp or to the aggregation location (can be called by disperse or doActivity at the end of a hunt).

```
to returnToCamp

  if debug [ print "returnToCamp" ] ; for debugging

  ; choose where to move to depending on what's happening right now
```

updateCampInteractions

Parameters interactionType is a string that can be “learning”, “hunting”, “trading”, or “visiting”. agentA and agentB are two different people doing the interaction.

Description Observer procedure. Update the camp interaction link every time agents interact

```
to updateCampInteractions [ interactionType agentA agentB ]

  let campA [ who ] of [ homeCamp ] of agentA ; gets the number of agentA's camp
  let campB [ who ] of [ homeCamp ] of agentB ; gets the number of agentB's camp

  if campA != campB [ ; Because people interacting within one camp will not be counted
↪  here
    ask campInteraction campA campB [ ; the remaining lines update the appropriate
↪  counter
      ifelse interactionType = "learning" [
        set learning learning + 1
      ][
        ifelse interactionType = "hunting" [
          set hunting hunting + 1
        ][
          ifelse interactionType = "trading" [
            set trading trading + 1
          ][
            set visiting visiting + 1
          ]
        ]
      ]
    ]
  ]

end
```

craftItem

Parameters itemType can be "point" or "pot" so it can be called individually. prob? is a boolean used to increase flexibility of the procedure (T: it happens with a certain probability, F: it happens automatically).

Description People procedure. People have a certain probability to create an object (pot or point). When created, the object records the current tick as well as its type (one of the types its maker knows).

```
to craftItem [ itemType prob? ]

  if debug [ print "craftItem" ] ; for debugging

  ; Every tick, each individual has a small chance to produce a new object (when prob? =
↪  true).
  ; Every time people acquire a new knowledge (when prob? = false), the create a new
↪  object

  let diceRoll random-float 100 ; for easier testing and troubleshooting

  ;; POINTS ;;
  ifelse itemType = "point" [
    if ( prob? and diceRoll < pNewObject ) or ( not prob? )[
      changeActivity "making a point"
```

```
      let fitTrait getFitTrait ; chooses the trait among the fitter ones (based on
↪  environment)
      set pointInventory lput ( list ticks fitTrait ) pointInventory ; add the point to
↪  its inventory (includes the tick number it was made)
    ]
    ; if that new creation pushed them above their toolkit limit, they drop the oldest
↪  point
    if length pointInventory > inventorySizePoints [ dropItem "point" false ]
  ][
    ;; POTS ;;
    if ( prob? and diceRoll < pNewObject ) or ( not prob? ) [
      changeActivity "making a pot"
      set potInventory lput ( list ticks one-of potTraits ) potInventory ; add the pot to
↪  its inventory (includes the tick number it was made)
    ]
    ; if that new creation pushed them above their toolkit limit, they drop the oldest
↪  pot
    if length potInventory > inventorySizePots [ dropItem "pot" false ]
  ]

end
```

migrate

Description People procedure. One person chooses another camp and trade place with someone from that camp. Migration can be gendered, in which case, only people with gender 1 trade places.

```
to migrate

  if debug [ print "migrate" ] ; for debugging

  changeActivity "migrating"
  let oldCamp homeCamp ; store old camp for swapping
  let possibleCamps 0 ; will take on the ID of possible camps
  ask homeCamp [ set possibleCamps other camps ]
  set homeCamp one-of possibleCamps ; choose one of the other camps at random as its new
↪  home
  set homeBand [ ownedBy ] of homeCamp
  if aggregation = false [ move-to homeCamp ] ; when not aggregating, move to the new
↪  home (when aggregating, stay within the aggregation camp for now)

  ; find someone who can take its old place
  let myID homeCamp ; the new camp
  let myNewCampmates people with [ homeCamp = myID ] ; all the people in the new home
                                                     ; select the migration partner (if
↪  gendered, only gender 1 can be selected)
```

recordCampPrestige

Description Camp procedure. Camps calculate the mean and max prestige of their people.

```
to recordCampPrestige
```

```
  let myPeople people with [ homeCamp = myself ] ; identify the people who belong to this
↪  camp
  let meanPrestigeHere precision (( sum [ pointPrestige ] of myPeople ) / campPopulation
↪  ) 2 ; Mean prestige of its people (rounded to 2 decimals)
  set meanPrestige lput meanPrestigeHere meanPrestige ; adds that value to a list
  let maxPrestigeHere ( max [ pointPrestige ] of myPeople ) ; Max prestige of its people
  set maxPrestige lput maxPrestigeHere maxPrestige ; add that value to a list

end
```

luckyLeap

Parameters objectType can be "point" or "pot". nextTrait is the trait that will be innovated (added to the list).

Description People procedure. People create a new object (point or pot) with the trait set to the given nextTrait. They then create a new object.

```
to luckyLeap [ nextTrait objectType ]

  if debug [ print "luckyLeap" ] ; for debugging

  ifelse objectType = "point" [
    changeActivity "inventing point"
    ; create a new main POINT type
    set pointTraits lput nextTrait pointTraits ; add the next number to the list
    set pointTraits remove-duplicates pointTraits ; make sure there are no duplicates
    if not table:has-key? pointTraitsTable nextTrait [
      table:put pointTraitsTable nextTrait [] ; if this is a brand new type, create a new
↪  entry in the table
    ]
    ; assign the new type to its environment if not already invented
    ; but only for points as pots' fitness does not change with environment
    if not table:has-key? traitEnvironments nextTrait [
      let numberHere [ environmentZone ] of patch-here ; record the environment where the
↪  type is created
      table:put traitEnvironments nextTrait numberHere ; list recording environment in
↪  which each main axis type was created
    ]
    craftItem "point" false ; then create a new point
  ][
    changeActivity "inventing pot"
    ; create a new main POT type
    set potTraits lput nextTrait potTraits ; add the next number to the list
    set potTraits remove-duplicates potTraits ; make sure there are no duplicates
    if not table:has-key? potTraitsTable nextTrait [
      table:put potTraitsTable nextTrait [] ; if this is a brand new type, create a new
↪  entry in the table
    ]
    craftItem "pot" false ; then create a new pot
  ]
```

```
end
```

createVariant

Parameters objectType can be “point” or “pot”.

Description People procedure. Add a variant to a mainType already known by ego. Then create an object.

```
to createVariant [ objectType ]

  if debug [ print "addVariant" ]

  changeActivity word "inventing variant" objectType

  ; set up temporary variables
  let reachedLimit? false ; will help stop the addition of variation when it reached the
↪  limit ("o" for sequential, and all letters used for non-sequential
  let newTypeVariation "" ; temp variable that will be filled later
  let newVariation "a" ; by default, the newVariation will be "a" so that it can be used
↪  if there is not already a variant

  ; select one known trait of the correct type
  let traitKit ifelse-value objectType = "point" [ pointTraits ][ potTraits ] ; define a
↪  temporary set of the relevant traits based on objectType
  let traitA one-of traitKit ; choose one known trait at random.
  let traitNumber getTraitNumber traitA ; focus only on the main number (the main type)
  let lastVariant getLastVariant traitA objectType ; gets the last variant that person
↪  has of the main type

  if lastVariant != "" [ ; if ego knew some variants...
    ifelse lastVariant != "o" [ ; ... and that variant is not the last possible one
↪  (there can only be 15 variations on each type)...
      set newVariation nextLetter lastVariant ; ...get the next possible letter
↪  alphabetically
    ][
      set reachedLimit? true ; if the type has reached its variation limit ("o"), the
↪  following block of code will not get called
    ]
  ]

  ; After the new type variant has been chosen (if not passed the limit), this updates
↪  the traitkit and the tables.
  if reachedLimit? = false [ ; only if the type has variations remaining (including "a")
↪  does it create a new one
    set newTypeVariation ( word traitNumber "-" newVariation ) ; add a letter to the
↪  type's variation
                                                                ; add the new trait to the
↪  relevant trait list
    ifelse objectType = "point" [
      ; POINT ;
      set pointTraits lput newTypeVariation pointTraits ; add the new trait to the
↪  person's known traits list
```

```
      ; determine if the point type already has an environment attached to it, and adds
↪  it if not
      if not table:has-key? traitEnvironments newTypeVariation [
        let numberHere [ environmentZone ] of patch-here ; record the environment number
        table:put traitEnvironments newTypeVariation numberHere ; list recording
↪  environment in which each type was created
      ]

      ; Add this new variant to the master table
      let listTraitKits table:get pointTraitsTable ( word traitNumber ) ; focus on all
↪  variations already done for this type
      let newTraitKit remove-duplicates ( sentence listTraitKits newVariation ) ; add the
↪  new type variation to that type's list only if it's not already there
      table:put pointTraitsTable ( word traitNumber ) newTraitKit  ; replace the list in
↪  the table by that updated one

      craftItem "point" false ; then create a new point

    ][ ; POT ;
      set potTraits lput newTypeVariation potTraits ; ads the new trait to the person's
↪  trait list

      ; Add this new type variant to the master table
      let listTraitKits table:get potTraitsTable ( word traitNumber ) ; focus on all
↪  variations already done for this type
      let newTraitKit remove-duplicates ( sentence listTraitKits newVariation ) ; add the
↪  new type variation to that type's list only if it's not already there
      table:put potTraitsTable ( word traitNumber ) newTraitKit  ; replace the list in
↪  the table by that updated one
    ]
  ]

end
```

loseTrait

Parameters traitsList represents the list of known trait by ego (either potTraits or pointTraits).

Return Returns the trait or traits that ego remembers after forgetting one trait (and its variant if it was a main type).

Description People procedure. People forget one of the traits they know, at random. If a main trait is forgotten, ego forgets its variants as well.

```
to-report loseTrait [ traitsList ]

  let lossTrait 0
  let rememberedTraitsList traitsList ; by default, if they do have enough traits to
↪  lose, they keep what they had

  if length traitsList > 1 [ ; do not remove trait if only one trait exists
```

```
    changeActivity "losing trait"
    set lossTrait one-of traitsList ; choose one of the known traits at random

    let listToFilter map [ i -> ifelse-value member? lossTrait i [ "loss" ][ i ]]
↪   traitsList ; creates a list with "loss" if the trait needs to be lost, and the trait
↪   if it is kept
    set rememberedTraitsList filter [ i -> i != "loss" ] listToFilter ; filters the list
↪   to keep only the traits remembered (remove all "loss")

    ; if they forgot the only main type they had and its variants (would happen a lot at
↪   first), they do not forget anything
    if length rememberedTraitsList = 0 [ set rememberedTraitsList traitsList ]
  ]

  report rememberedTraitsList

end
```

getNextNumber

Parameters objectType can be "pot" or "point".

Return Returns the max number already known + 1.

Description People procedure. Called with LuckyLeap only. It finds the next number (type) to innovate.

```
to-report getNextNumber [ objectType ] ; People reporter, reports the next number for the
↪   objectType (can be "pot" or "point")(for luckyLeap)

  let tempListTraits [] ; temp variable to allow using this reporter for both points and
↪   pots
  ifelse objectType = "point" [
    set tempListTraits pointTraits ; populate it with point traits
  ][
    set tempListTraits potTraits ; populate it with point traits
  ]

  let listOfNumbers ( map [ trait -> getTraitNumber trait ] tempListTraits ) ; transform
↪   the traits into their main trait number
  let nextNumber ( max listOfNumbers ) + 1

  report ( word nextNumber ) ; get the max number already known and add 1 for the next
↪   (as a string)

end
```

getFitTrait

Return Returns one point type with a relatively high fitness, given the current environment.

Description People procedure. Looks at the person's pointTraits's fitnesses in the current environment, and then chooses one of the traits from that list (fit traits have more chance to be selected than non-fit traits).

```
to-report getFitTrait

  if debug [ print "getFitTrait" ] ; for debugging

  let myZone [ environmentZone ] of patch-here ; environmental zone of the current
↪  location

  let traitFitnessList ( map [ x -> table:get traitEnvironments x ] pointTraits ) ; this
↪  extracts the environment of each point trait known

  ; for each point trait known, it replaces the trait with its fitness (5 if the trait
↪  was invented in the same environment as myZone, 2 if not)
  set traitFitnessList map [ x -> ifelse-value ( x = myZone ) [ 5 ] [ 2 ]]
↪  traitFitnessList
  let pairs ( map list pointTraits traitFitnessList ) ; Pairs the pointTraits counters
↪  with their current fitness
  let fitTrait first rnd:weighted-one-of-list pairs [[ p ] -> last p ] ; chooses one
↪  trait, favoring the fitter ones (based on environment)

  report fitTrait ; report the chosen trait

end
```

deconstructingTrait

Parameters trait is a trait known by the person calling this reporter. It can be either a point or a pot trait (not the object made with it).

Return Returns the last part of the trait in its correct format (either a letter as a string or a number as a number).

Description People procedure. Used to define if a trait is a main type or a type with a variant, so the person innovating onto it can add the correct variant (called in createVariant).

```
to-report deconstructingTrait [ trait ] ; people procedure called in createVariant to set
↪  the last item to its type (string or number) without erroring-out

  let traitWithType 0 ; temporary variable

  carefully [ ; this allows separating the trait without creating errors (if the trait is
↪  a main type, it should not be read-from-string).
    set traitWithType read-from-string last trait ; that's when the trait is a main type,
↪  which is transformed into a number (was a string of the number)
  ][
    set traitWithType last trait ; that's when the trait has a variant, which is a string
  ]

  if traitWithType = e [ set traitWithType "e" ] ; e is read as a number, so this fixes
↪  the error it would cause.

  report traitWithType
```

```
end
```

getTraitNumber

Parameters trait is a trait known by the person calling this reporter. It can be either a point or a pot trait (not the object made with it).

Return Returns the number of the trait as a number (main type).

Description People procedure. Gets the main type of the object (without variants).

```
to-report getTraitNumber [ trait ]

  ; first identify the position of the "-" (when there is a variant) or the length of the
↪  trait (when it is a main type)
  let p position "-" trait
  if p = false [ set p length trait ] ; if there was no "-", p became "false," so this
↪  gives it the length of the whole trait

  ; then extract the part of the trait up to p (a.k.a., up to the "-" or the whole trait)
  let traitN substring trait 0 p ; removes the variation letter and keeps only the number
  set traitN read-from-string traitN ; transform the string into its number form

  report traitN

end
```

nextLetter

Parameters currentLetter is the letter of the variant upon which the person will innovate.

Return returns the next letter alphabetically.

Description People procedure. Helps find the next letter when innovating on a trait with variant.

```
to-report nextLetter [ currentLetter ]

  let thisPosition position currentLetter traitLetters ; finds the position of the
↪  currentLetter in the alphabetical list set earlier
  let nextPosition thisPosition + 1 ; get the next position

  report item nextPosition traitLetters ; returns the letter following currentLetter
↪  alphabetically

end
```

traitVisibility

Parameters trait is one trait known by ego (can be for points or pots). The trait needs to be one with variant.

Return Returns the visibility assigned to the trait provided.

Description People procedure. Identifies the visibility of a certain trait. Called by checkVisibility only for traits with variants.

```
to-report traitVisibility [ trait ]
  let result 0
  carefully [set result table:get visibilityTable trait][; get the assigned visibility of
↪  that variant
    set result 0
  ]
  report result

end
```

getMostlyVisibleTraits

Parameters traitList can be a pointTraits list or the potTraits list.

Return Returns a list of some of the most visible traits from the traitList provided.

Description People procedure. Samples traits from the traitList with repeat. Visible traits have higher chances to get selected.

```
to-report getMostlyVisibleTraits [ traitList ]



  ;  ; all main types are highly visible, so we can automatically separate them to assign
↪  visibility
  ;  let mainTypes filter [ x -> is-number? deconstructingTrait x ] traitList
  ;
  ;  let variantTraits filter [ x -> is-string? deconstructingTrait x ] traitList ;
↪  filters only the traits with variants

  ; for variants

  let traitsVisibility ( map [ x -> traitVisibility x ] traitList ) ; computes each
↪  variants' visibility

  let traitsPairedVisibility ( map list traitList traitsVisibility ) ; pairs each variant
↪  with its visibility

  ; filter out low visibility traits
  let traitsVisibile []

  foreach traitsPairedVisibility [
    x -> if item 1 x = 3 [
      set traitsVisibile insert-item 0 traitsVisibile item 0  x
    ]
  ]
```

```
  ; combine mainTypes and high-visibility variants
  ;  let visibleTraits sentence mainTypes traitsVisibile
  report traitsVisibile

end
```

getVariantsOfMainType

Parameters trait is the trait considered, objectType can be “point” or “pot”.

Return Returns a list of all the variants of the trait’s mainType already known by ego (NOT all variants created in the simulation).

Description People procedure. Identifies all the variants of trait’s mainType known by ego.

```
to-report getVariantsOfMainType [ trait objectType ]

  ; first get the mainType number
  let mainTypeNumber getTraitNumber trait

  ; create a temp variable that takes on the traits considered here, based on what the
↪  objectType is
  let traits ifelse-value objectType = "point" [ pointTraits ][ potTraits ]

  ; then produce a list of that type's variants (from the relevant list)
  let mainTypeVariants map [ i -> ifelse-value getTraitNumber i = mainTypeNumber [
↪  deconstructingTrait i ][ "" ]] traits

  ; This list now has the variants of the main tool or the main tool itself, and "" for
↪  all other tools
  report mainTypeVariants

end
```

getLastVariant

Parameters trait is the trait considered, objectType can be “point” or “pot”.

Return Returns the last variant ego knows of the type of the trait provided (variant based on alphabetical order).

Description People procedure. Identifies the last variants ego knows of the type of the given trait.

```
to-report getLastVariant [ trait objectType ]

  let variants getVariantsOfMainType trait objectType ; call the reporter to get a list
↪  of ego's traits that contains the mainType, its variants, and "" for anything else
  let positionVariants map [ i -> position i traitLetters ] variants ; get the position
↪  of each variant in the alphabet (returns false if the variant is not part of the
↪  alphabet)
  let maxVariant ""
```

```
  carefully [ ; if the type of the trait did not have a variant, this prevents an error
↪  and goes to get any other variants that would be for that type
    let maxVariantPos max positionVariants ; get the max position (furthest along the
↪  alphabet)
    set maxVariant item maxVariantPos traitLetters ; get the variant associated with that
↪  position in the alphabet
  ][  ] ; there is nothing in the second bracket, because if there is an error in the
↪  first, nothing changes (maxVariant remains "")

  report maxVariant

end
```

updateVisibility

Description Observer procedure. Assigns visibility of 1 (low visibility) or 2 (high visibility) to a trait or variant.

```
to updateVisibility

  let a remove-duplicates reduce sentence [potTraits] of people
  let b remove-duplicates reduce sentence [pointTraits] of people
  let allTraits remove-duplicates sentence a b

  foreach allTraits [
    k ->
    if not table:has-key? visibilityTable k [
      table:put visibilityTable k (random 2) + 1 ; if this is a brand new type, create a
↪  new entry in the table
    ]
  ]

end
```

transmitCulture

Description People procedure. Calls the appropriate procedure to transmit traits based on the transmissionRate, learning method, and object selected.

```
to transmitCulture

  if debug [ print "transmitCulture" ] ; for debugging
  let newTrait 0
  ;; POT ;;

  if random-float 100 < transmissionRate [ ; If they are learning from someone
    changeActivity "learning pots"

    ; add one learned trait (the source varies based on the learningMethod)
    set newTrait ifelse-value random-float 100 < learningMethod [ transmitPrestige "pot"
↪  ][ transmitConformism "pot" ]
    if newTrait != 0 [
```

```
      set potTraits lput newTrait potTraits

      craftItem "pot" false ; then create a new pot
      set potTraits remove-duplicates potTraits ; make sure that no duplicates are added
↪  through transmission
    ]
  ]

  ;; POINT ;;

  if random-float 100 < transmissionRate [ ; If they are learning from someone
    changeActivity "learning points"
    ; add one learned trait (the source varies based on the learningMethod)
    set newTrait ifelse-value random-float 100 < learningMethod [ transmitPrestige
↪  "point" ][ transmitConformism "point" ]
    if newTrait != 0 [
      set pointTraits lput newTrait pointTraits
      craftItem "point" false ; then create a new point
      set pointTraits remove-duplicates pointTraits ; make sure that no duplicates are
↪  added through transmission
    ]
  ]

end
```

transmitConformism

Parameters objectType can be “point” or “pot”

Return Returns the most common trait

Description People procedure. Ego identifies the most common trait known by most people at the same spot. All people who contributed add 1 to their learning counter with ego.

```
to-report transmitConformism [ objectType ]

  if debug [ print "transmitConformism" ] ; for debugging

  let learnedTrait 0
  let traitList []
  let currentTraits []

  ask people-here [
    ; everyone (including the agent asking) adds their traits to the list (if visibility
↪  is taken into consideration, the list has more visible traits
    set currentTraits ifelse-value objectType = "point" [
      ifelse-value visibility and random-float 100 < 98 [ getMostlyVisibleTraits
↪  pointTraits ][ pointTraits ]
    ][
      ifelse-value visibility  and random-float 100 < 98 [ getMostlyVisibleTraits
↪  potTraits ][ potTraits ]
    ]
```

```
    set traitList sentence traitList currentTraits ; adds the traits to the list
  ]
  if not empty? currentTraits [
    set learnedTrait one-of modes traitList ; choose the trait known the most (mode) by
↪  everyone

    ; update the interaction links counter
    ask other people-here [
      updateCampInteractions "learning" self myself ; update the counter of the camps
      ask interaction-with myself [ set learning learning + 1 ] ; identify all links from
↪  the people here to the person asking
    ]
  ]

  report learnedTrait ; reports that trait

end
```

transmitPrestige

Parameters objectType can be “point” or “pot”.

Return Returns the trait chosen from the person with higher prestige.

Description People procedure. Ego identifies the person with highest prestige around and takes one of its traits. Ego adds 1 to its learning counter with the person with prestige.

```
to-report transmitPrestige [ objectType ]

  if debug [ print "transmitPrestige" ] ; for debugging

  let learnedTrait 0 ; variable for the new trait
  let currentTraits []
  let teacher 0

  ifelse objectType = "point" [
    set teacher one-of people-here with-max [ pointPrestige ] ; prestige is updated at
↪  each hunt
    set currentTraits [ pointTraits ] of teacher
  ][
    ; set the teacher based on if potteryPrestige is ON or not
    set teacher ifelse-value potteryPrestige [ one-of people-here with-max [ potPrestige
↪  ]][ one-of people-here ]
    set currentTraits [ potTraits ] of teacher
  ]

  if visibility  and random-float 100 < 98 [ set currentTraits getMostlyVisibleTraits
↪  currentTraits ] ; if visibility is in play, this reduces the traits to the most
↪  visible
  if not empty? currentTraits [

    ; choose amongst the teacher's traits one that has higher fitness based on the
↪  current environment (points) or at random (pots)
```

```
    set learnedTrait ifelse-value objectType = "point" [ getFitTrait ][ one-of
↪  currentTraits ]

    ; update the interaction link counter
    if teacher != self [
      ask teacher [
        updateCampInteractions "learning" self myself ; update the counter of the camps
        ask interaction-with myself [ set learning learning + 1 ] ; identify the link
↪  from the teacher to the person asking
      ]
    ]
  ]
  report learnedTrait

end
```

totalTraits

Parameters trait can be "potTraits" or "pointTraits".

Return Returns the number of traits currently known by the population.

Description Observer procedure. Called by the Trait counts plot.

```
to-report totalTraits [ trait ]

  let traits length remove-duplicates reduce sentence trait ; calculates the number of
↪  DIFFERENT traits known by the population

  report traits

end
```

printMetrics

Description Observer procedure. Print multiple metrics in the Command Center.

```
to printMetrics

  print word "assemblageSizePoints: " assemblageSize "point"
  print word "assemblageSizePots: " assemblageSize "pot"
  print word "elapsedTime: " timer
  print word "interactionFreqHunting: " interactionFreq "hunting"
  print word "interactionFreqLearning: " interactionFreq "learning"
  print word "interactionFreqTrading: " interactionFreq "trading"
  print word "interactionFreqVisiting: " interactionFreq "visiting"
  print word "brDistBandPoints: " getBRBands "point"
  print word "brDistBandPots: " getBRBands "pot"
  print word "brDistCampPoints: " getBRCamps "point"
  print word "brDistCampPots: " getBRCamps "pot"
  print word "meanPeoplePrestige: " precision (mean [ pointPrestige ] of people) 3
```

```
  print word "modBandPoint: " modularityBand "point"
  print word "modBandPot: " modularityBand "pot"
  print word "modCampPoint: " modularityCamp "point"
  print word "modCampPot: " modularityCamp "pot"
  print word "networkMetricPoint: " networkMetric "point"
  print word "networkMetricPot: " networkMetric "pot"
  print word "PhiPointCamp: " PhiSTCamps "point"
  print word "PhiPotCamp: " PhiSTCamps "pot"
  print word "PhiPointBand: " PhiSTBands "point"
  print word "PhiPotBand: " PhiSTBands "pot"
  print word "nTraitsPoints: " nTraits "point"
  print word "nTraitsPots: " nTraits "pot"

end
```

assemblageSize

Parameters objectType can be “pot” or “point”.

Return Returns the number of objects discarded at camps and bands.

Description Observer procedure. Calculates the number of objects (pot or point) discarded at camps and bands.

```
to-report assemblageSize [ objectType ] ; objectType can be "pot" or "point"

  let assemblageLength 0 ; will take on all the camps and bands' assemblage lengths

  ask camps [ ; camps add their assemblage length first
    set assemblageLength ifelse-value objectType = "pot" [ assemblageLength + length
↪  potAssemblage ][ assemblageLength + length pointAssemblage ]
  ]

  ask bands [ ; then bands
    set assemblageLength ifelse-value objectType = "pot" [ assemblageLength + length
↪  potAssemblage ][ assemblageLength + length pointAssemblage ]
  ]

  report assemblageLength

end
```

interactionFreq

Parameters activityName is a string describing the activity for which we want the interactions (hunting, learning, trading, or visiting).

Return Returns the rounded sum of all interactions of the given activity.

Description Observer procedure. Calculates the sum of the interactions of the given activity.

```
to-report interactionFreq [ activityName ]

  let sumInteractions (ifelse-value
    activityName = "hunting" [ sum [ hunting ] of interactions ] ; take on the sum of all interactions of the given activity, based on the name given
    activityName = "learning" [ sum [ learning ] of interactions ]
    activityName = "trading" [ sum [ trading ] of interactions ]
    activityName = "visiting" [ sum [ visiting ] of interactions ])

  report precision sumInteractions 3 ; return that value, rounded to the nearest 3 decimals.

end
```

getBRBands

Parameters objectType can be "pot" or "point".

Return Returns the mean of all inter-band distances (Brainerd-Robinson). Scaled where 1 is most similar, and 0 is no similiarity.

Description Observer procedure. Computes the Brainerd-Robinson similiarity between bands.

```
to-report getBRBands [ objectType ]

  ; temporary variable
  let allDist []

  ; create links between bands and calculate distances between each linked band
  ask bands [
    create-tempLinks-with other bands [ set hidden? false ] ; this makes sure distances are not calculated twice
    set allDist lput calculateBR objectType allDist ; call the procedure that actually calculates the distance and add it to the master list
  ]

  set allDist ( reduce sentence allDist ) ; remove all square brackets in the master list (that were added as each band reports 2 distances as one list)
  report precision mean allDist 3 ; report the mean of all inter-band distances

end
```

getBRCamps

Parameters objectType can be "pot" or "point".

Return Returns the mean of all inter-CAMP distances (Brainerd-Robinson). Scaled where 1 is most similar, and 0 is no similiarity.

Description Observer procedure. Computes the Brainerd-Robinson similiarity between camps of the same band.

```
to-report getBRCamps [ objectType ]

  ; temporary variable
  let allDist []

  ; calculate distances between each camp within the same band and calculate distances
↪  between each linked camp
  ask camps [
    create-tempLinks-with other camps with [ ownedBy = [ ownedBy ] of myself ] [ set
↪  hidden? false ] ; this makes sure distances are not calculated twice
    set allDist lput calculateBR objectType allDist ; call the procedure that actually
↪  calculates the distance and add it to the master list
  ]

  set allDist ( reduce sentence allDist ) ; remove all square brackets in the master list
↪  (that were added as each camp reports multiple distances as one list)
  report precision mean allDist 3 ; report the mean of all inter-band distances

end
```

calculateBR

Parameters objectType can be "pot" or "point".

Return Returns a list of distances (Brainerd-Robinson) between the agent asking and its linked agents. Scaled where 1 is most similar, and 0 is no similiarity.

Description Observer procedure. Computes the Brainerd-Robinson similiarity of the objectType between linked agents.

```
to-report calculateBR [ objectType ]

  ; populate variables with all distinct types of traits from people in that camp or band
  listTraits

  ; temporary variables used below
  let allTraitsA 0
  let allTraitsB 0
  let allDist []
  let dist 0

  ; calculate Brainerd-Robinson distances between linked bands or camps
  set allTraitsA ifelse-value objectType = "point" [ allPoints ][ allPots ] ; take on the
↪  traits of the band/camp's people
  set allTraitsA getTraitFreqTable allTraitsA ; get the frequency of each trait in the
↪  list
  ask tempLink-neighbors [ ; asks the linked agents (bands or camps)
    set allTraitsB ifelse-value objectType = "point" [ allPoints ][ allPots ] ; take on
↪  the traits of the band/camp's people
    set allTraitsB getTraitFreqTable allTraitsB ; get the frequency of each trait in the
↪  list
```

```
    set dist getFreqDistList allTraitsA allTraitsB ; get a list of the differences in
↪  frequency for each trait in both lists
    set dist ifelse-value ( sum dist = 0 )[ 0 ][ 1 - ( sum dist / 200 )] ; compute the
↪  Brainerd-Robinson dist with the list provided
    set allDist lput dist allDist ; add the dist calculated to the allDist list
  ]

  ask tempLinks [ die ] ; always remove the links after we used them
  report allDist

end
```

listTraits

Description Observer procedure. Tells bands and camps to create a list with all the traits known by their people (one list for point and one list for pot). Then collate this list into a table that has the frequency of each trait type.

```
to listTraits

  let traitList []
  ask bands [
    set traitList [ pointTraits ] of people with [ homeBand = [ who ] of myself ] ;
↪  compile all the pointTraits of people from this band
    set allPoints reduce sentence traitList ; remove all the square brackets around
↪  individual traitlists
    set allPoints table:counts allPoints ; count the number of each trait and enter it as
↪  a table
    set traitList [ potTraits ] of people with [ homeBand = [ who ] of myself ] ; compile
↪  all the potTraits of people from this band
    set allPots reduce sentence traitList ; remove all the square brackets around
↪  individual traitlists
    set allPots table:counts allPots ; count the number of each trait and enter it as a
↪  table
  ]

  ask camps [ ; comments are the same as above, but for camps
    set traitList [ pointTraits ] of people with [ homeCamp = myself ]
    set allPoints reduce sentence traitList
    set allPoints table:counts allPoints
    set traitList [ potTraits ] of people with [ homeCamp = myself ]
    set allPots reduce sentence traitList
    set allPots table:counts allPots
  ]

end
```

getTraitFreqTable

Parameters allTraits is a table of all traits collected by either the camp or band. That table has the traits known by their people (key) and the count of those traits (value).

Return Returns a table with the frequency of each trait given as a percentage rather than a count.

Description Camp AND Band procedure. Transforms the count of each trait into a percentage of known traits (frequency).

```
to-report getTraitFreqTable [ allTraits ]

  let sumCountTraits sum table:values allTraits ; the sum count of all traits

  foreach table:keys allTraits [ key -> ; for each key in the table
    let valCount table:get allTraits key ; get the count associated with the key
    table:put allTraits key (( valCount / sumCountTraits ) * 100 ) ; replace the count
↪   associated with each key with its frequency (percentage of all known traits)
  ]

  report allTraits

end
```

getFreqDistList

Parameters traitsTableA and traitsTableB are two tables of traits with key = traits, and values = frequency of that trait known by the people of the band/camp asking.

Return Returns a list of the frequency differences for all traits found in the two tables.

Description Band AND Camp procedure. Calculate the difference in popularity between all items in two tables.

```
to-report getFreqDistList [ traitsTableA traitsTableB ]

  ; temporary variables
  let dist []
  let tmpA 0
  let tmpB 0

  let allKeys remove-duplicates sentence table:keys traitsTableA table:keys traitsTableB
↪   ; get all keys in both tables of proportions

  foreach allKeys [ key -> ; for each key, this calculates the difference between tables
↪   A and B
    set tmpA ifelse-value table:has-key? traitsTableA key [ table:get traitsTableA key ][
↪   0 ] ; take the value associated with the key in table A, if present
    set tmpB ifelse-value table:has-key? traitsTableB key [ table:get traitsTableB key ][
↪   0 ] ; take the value associated with the key in table B, if present
    set dist lput abs ( tmpA - tmpB ) dist ; calculate their difference and add to the
↪   list
  ]

  report dist

end
```

modularityBand

Parameters objectType can be "pot" or "point".

Return Returns the modularity of bands. Modularity is "defined based on the number of in-community links versus the number of between-community links" (manual).

Description Observer procedure. Calculates the modularity of bands, based on the traits known. Creates links between people based on the similarity of their known traits and uses those links to calculate modularity.

```
to-report modularityBand [ objectType ] ; objectType can be "pot" or "point"

  let modul -999 ; because 0 is an actual possible modularity measure, this is used to
↪  troubleshooting (if this returns -999, there is a problem)

  ; reset the tempLinks, which only keeps the links with 25th percentile Jaccard distance
↪  (the most similar)
  resetTempLinksForModularity objectType
  nw:set-context people tempLinks ; set the context to compute network metrics on
  let peoplePerBands []
  ask Bands [ set peoplePerBands lput people with [ homeband = [ who ] of myself ]
↪  peoplePerBands ] ; group people per band
  set modul nw:modularity peoplePerBands ; calculate modularity by considering the number
↪  of WITHIN-BAND links versus the number of BETWEEN-BAND links

  ask tempLinks [ die ] ; always remove the links after we used them

  report modul

end
```

modularityCamp

Parameters objectType can be "pot" or "point".

Return Returns the modularity of camps. Modularity is "defined based on the number of in-community links versus the number of between-community links" (manual).

Description Observer procedure. Calculates the modularity of camps, based on the traits known. Creates links between people based on the similarity of their known traits and uses those links to calculate modularity.

```
to-report modularityCamp [ objectType ]

  let modul -999 ; because 0 is an actual possible modularity measure, this is used to
↪  troubleshooting (if this returns -999, there is a problem)

  ; reset the tempLinks, which only keeps the links with 25th percentile Jaccard distance
↪  (the most similar)
  resetTempLinksForModularity objectType

  nw:set-context people tempLinks ; set the context to compute network metrics on
  let peoplePerCamps []
```

```
  ask camps [ set peoplePerCamps lput people with [ homecamp = myself ] peoplePerCamps ]
↪  ; groups people per camp
  set modul nw:modularity peoplePerCamps ; calculate modularity by considering the number
↪  of WITHIN-CAMP links versus the number of BETWEEN-CAMP links

  ask tempLinks [ die ] ; always remove the links after we used them

  report modul

end
```

resetTempLinksForModularity

Parameters objectType can be “pot” or “point”.

Description Observer procedure. Calculates the Jaccard distance between traits of people. The most similar create a new set of links used to compute modularity.

```
to resetTempLinksForModularity [ objectType ] ; objectType can be "pot" or "point"

  ask interactions [
    setupJaccardDistBetweenTraits objectType
  ]

  let interactionsJaccardList [( list jaccard )] of interactions ; get a list of all
↪  interaction Jaccards
  let tbl stats:newtable-from-row-list interactionsJaccardList ; transform the list into
↪  a stat table, necessary for the next step (quantile)
  let threshold stats:quantile tbl 0 25 ; compute the Jaccard threshold (the 25th
↪  quantile)
  ask interactions with [ jaccard <= threshold ][ ask end1 [ create-tempLink-with [ end2
↪  ] of myself ]] ; create tempLinks between people with jaccard distance below
↪  threshold
```

networkMetric

Parameters objectType can be “pot” or “point”.

Return Returns a value between -1 and 1. If it is close to 0, it means the network from artifacts represents relatively well the network from interactions, if it is positive, the network from interactions is more clustered than the network from artifacts, if it is negative, the network from artifacts is more clustered than the network from interactions.

Description Observer reporter. Calculates the difference between the clustering coefficient of the network of people and the clustering-coefficient of the network reconstructed from similar objects.

```
to-report networkMetric [ objectType ]

  ; if it is close to 0, it means the network from artifacts represents relatively well
↪  the network from interactions
  ; if it is positive, the network from interactions is more clustered than the network
↪  from artifacts
```

```
  ; if it is negative, the network from artifacts is more clustered than the network from
↪  interactions

  ifelse objectType = "point" [
    nw:set-context people ( interactions with [ hunting > 0 ]) ; sets the context through
↪  which the metrics will be computed (hunting interactions when looking at points)
  ][
    nw:set-context people ( interactions with [ learning > 0 ]) ; sets the context
↪  through which the metrics will be computed (learning interactions when looking at
↪  pots)
  ]

  let globalClustering mean [ nw:clustering-coefficient ] of people ; calculate the
↪  clustering-coefficient of the whole network

  ; compute the clustering coefficient between camps on the basis on their shared items
  ask campInteractions [
    let A ifelse-value objectType = "point" [[ pointAssemblage ] of end1 ] [[
↪  potAssemblage ] of end1 ]
```

setupJaccardDistBetweenTraits

Parameters objectType can be "pot" or "point".

Description Link procedure. Calculates the Jaccard distance between traits of agents linked by the link asking.

```
to setupJaccardDistBetweenTraits [ objectType ] ; objectType can be "pot" or "point"

  let A ifelse-value objectType = "point" [[ pointTraits ] of end1 ] [[ potTraits ] of
↪  end1 ] ; get the correct traits to compare
```

setupJaccardDistBetweenObjects

Parameters objectType can be "pot" or "point".

Description Link procedure. Calculates the Jaccard distance between assemblages (objects) of agents linked by the link asking.

```
to setupJaccardDistBetweenObjects [ objectType ] ; objectType can be "pot" or "point"

  let A ifelse-value objectType = "point" [[ pointAssemblage ] of end1 ] [[ potAssemblage
↪  ] of end1 ]
```

jaccardDist

Parameters A and B are two lists (of traits or objects discarded) to be compared.

Return Returns the Jaccard distance between the two lists.

Description Observer procedure. This calculates the Jaccard distance between two lists (A and B).

```
to-report jaccardDist [ A B ] ; A and B represent two lists

  ; The Jaccard distance is 1- Jaccard Index
  ; The Jaccard index for two sets of numbers is: (the Union of both sets - the
↪  Intersection of both sets)/The Union of both sets

  let AIB [] ; this will be the intersection of both sets
  let AUB 0 ; this will be the union of both sets

  foreach A [ x ->
    if member? x B [ set AIB lput x AIB ] ; if the item is shared, it is added to the
↪  list
  ]

  set AIB length AIB ; the number of shared items
  set AUB length ( remove-duplicates ( sentence A B )) ; the total number of items

  ; calculate the Jaccard index if there are traits already
  let JDist ifelse-value AUB = 0 [ 2 ][ ( AUB - AIB ) / AUB ] ; to avoid errors if there
↪  are no traits (e.g., at beginning of a simulation)

  report JDist

end
```

jaccardDistWeighted

Parameters A and B are two lists (of traits or objects discarded) to be compared.

Return Returns the weighted Jaccard distance between the two lists.

Description Observer procedure. This calculates the weighted Jaccard distance between two lists (A and B).

```
;; SHOULD NOT BE USED FOR PEOPLE INTERACTIONS AS IT IS VERY SLOW
to-report jaccardDistWeighted [ A B ] ; A and B represent two lists

  ; The weighted Jaccard index is the sum of the minimum of each set divided by the sum
↪  of the max of each set
  ; 1 - Jaccard index is the similarity value

  ; faster?
  let tblA getTraitFreqTable table:counts A ; get a table of proportions of each trait in
↪  list
  let tblB getTraitFreqTable table:counts B ; get a table of proportions of each trait in
↪  list
  let allKeys remove-duplicates ( sentence table:keys tblA table:keys tblB ) ; get all
↪  keys in both tables of proportions

  ; will be used to calculate min and max of each set
```

```
  let minVals 0
  let maxVals 0

  foreach allKeys [ key -> ; for each key, this calculates the min and max value between
↪  tables A and B
    let tmpA ifelse-value table:has-key? tblA key [ table:get tblA key ][ 0 ] ; take the
↪  value associated with the key in table A, if present
    let tmpB ifelse-value table:has-key? tblB key [ table:get tblB key ][ 0 ] ; take the
↪  value associated with the key in table B, if present
    set minVals minVals + ( min ( list tmpA tmpB )) ; calculate the min of the two values
↪  and add to the previous value (cumulative sum)
    set maxVals maxVals + ( max ( list tmpA tmpB )) ; calculate the max of the two values
↪  and add to the previous value (cumulative sum)
  ]

  let JDist minVals / maxVals ; calculate the weighted Jaccard distance

  report precision JDist 3

end
```

PhiSTCamps

Parameters objectType can be “pot” or “point”.

Return Returns the PhiST statistic. If it is close to 0, variation is not significantly structured around camps.

Description Observer reporter. Calculate PhiST statistic between people’s inventories according to the camp they belong to.

```
to-report PhiSTCamps [ objectType ]

  let phi -999 ; for troubleshooting to see errors

  carefully [
    ; first compute jaccard among people between camps
    ask people [ ; create tempLinks between people who are NOT from the same camp
      let peopleOtherCamps other people with  [ homeCamp != [ homeCamp ] of myself ]
      create-tempLinks-with peopleOtherCamps
    ]

    ; calculate jaccard between traits of those linked people
    ask tempLinks [ setupJaccardDistBetweenTraits objectType ]

    ; calculate variance in jaccard distances
    let j [ jaccard ] of tempLinks
    let varBetween variance j
    ask tempLinks [ die ] ; reset the tempLinks

    ; now variance within camps
    ask people [ ; create tempLinks between people who ARE from the same camp
```

```
      let peopleMyCamp other people with  [ homeCamp = [ homeCamp ] of myself ]
      create-tempLinks-with peopleMyCamp
    ]

    ; calculate jaccard between traits of those linked people
    ask tempLinks [ setupJaccardDistBetweenTraits objectType ]

    ; calculate variance in jaccard distances
    let k [ jaccard ] of tempLinks
    let varWithin variance k
    ask tempLinks [ die ] ; always remove the links after we used them

    ; calculate PhiST
    set phi ( varBetween - varWithin ) / ( varBetween )

  ][ show "error in PhiST" ]

  report phi

end
```

PhiSTBands

Parameters objectType can be “pot” or “point”.

Return Returns the PhiST statistic between bands. If it is close to 0, variation is not significantly structured around camps.

Description Observer reporter. Calculate PhiST statistic between people’s inventories according to the camp they belong to.

```
to-report PhiSTBands [ objectType ] ; if it is close to 0, variation not significantly
↪  structured around camps

  let phi -999 ; for troubleshooting to see errors

  carefully [
    ; first compute jaccard among people between bands
    ask people [ ; create tempLinks between people who are NOT from the same band
      let peopleOtherBands other people with  [ homeBand != [ homeBand ] of myself ]
      create-tempLinks-with peopleOtherBands
    ]

    ; calculate jaccard between traits of those linked people
    ask tempLinks [ setupJaccardDistBetweenTraits objectType ]

    ; calculate variance in jaccard distances
    let j [ jaccard ] of tempLinks
    let varBetween variance j
    ask tempLinks [ die ] ; reset the tempLinks

    ; now variance within bands
```

```
    ask people [ ; create tempLinks between people who ARE from the same band
      let peopleMyBand other people with  [ homeBand = [ homeBand ] of myself ]
      create-tempLinks-with peopleMyBand
    ]

    ; calculate jaccard between traits of those linked people
    ask tempLinks [ setupJaccardDistBetweenTraits objectType ]

    ; calculate variance in jaccard distances
    let k [ jaccard ] of tempLinks
    let varWithin variance k
    ask tempLinks [ die ] ; always remove the links after we used them

    ; calculate PhiST
    set phi ( varBetween - varWithin ) / ( varBetween )

  ][ show "error in PhiST" ]

  report phi

end
```

nTraits

Parameters objectType can be "pot" or "point".

Return Returns the number of traits known of the given objectType. It considers repetitions (e.g., if 15 agents all know trait "1" only, the result will be 15).

Description Observer procedure. Calculates the number of traits known by the whole population (with repetitions).

```
to-report nTraits [ objectType ] ; objectType can be "pot" or "point"

  let nList []

  ask people [ ; each person adds their known trait to the list
    set nList ifelse-value objectType = "pot" [ sentence nList potTraits ][ sentence
↪  nList pointTraits ]
  ]

  report length nList

end
```

compileArtifactSimilarities

Description Observer procedure. Calculate the similarity of objects between camps, keep only the most similar and update the relevant adjacency tables with the similarity data.

```
to compileArtifactSimilarities
```

```
  ; For points
  ask campInteractions [
    setupJaccardDistBetweenObjects "point" ; compute the similar objects
    updateAdjacencyTables campPointAdjacency jaccard ; and that that value to the table
  ]

  ; For pots
  ask campInteractions [
    setupJaccardDistBetweenObjects "pot" ; compute the similar objects
    updateAdjacencyTables campPotAdjacency jaccard ; and that that value to the table
  ]

end
```

compileCampInteractions

Description Observer procedure. Asks the links between camps to update the adjacency tables with their counters.

```
to compileCampInteractions

  ask campInteractions [ ; each link between camps add their counter value to the
↪  adjacency table
    updateAdjacencyTables campVisitingAdjacency visiting
    updateAdjacencyTables campTradingAdjacency trading
    updateAdjacencyTables campHuntingAdjacency hunting
    updateAdjacencyTables campLearningAdjacency learning
  ]

end
```

updateAdjacencyTables

Parameters matrixName is the name of the adjacency table getting updated, and addedValue is the value that will be added to the table.

Description Link procedure. Updates the values in the adjacency table given (matrixName) by adding addedValue to the values already there.

```
to updateAdjacencyTables [ matrixName addedValue ]

  let rowN [ who ] of end1 - nOfBands ; get the row number associated with the camp at
↪  one end (as camps are created after bands, their numbers follow the band count)
```

fileNameBase

Return Returns a filename with the following structure: “day-month-year-time”.

Description Observer procedure. Takes the current time and produces a name with it.

```
to-report fileNameBase

  let dt (remove ":" remove " " date-and-time)
  let t substring dt 0 12
  let d substring dt 12 length dt
  report (word d "-" t)

end
```

exportResults

Description Observer procedure. Exports variable and values to a CSV file with filename set by fileNameBase.

```
to exportResults

  let fileName fileNameBase ; create a new file name based on date and time
  print ( word "file name is " fileName )
  let fileNameCSV ( word "results/run_outputAssemblages_" fileName ".csv" ) ; the
↪  complete CSV file name
  if not file-exists? fileNameCSV [ ; so it does not overwrite it, if it already exists.

    ; save assemblages
    file-open fileNameCSV
    file-print ( "band,site,pointAssemblage,potAssemblage,MeanPrestige,MaxPrestige" )
    ask camps [ file-print ( word ownedBy "," who "," pointAssemblage "," potAssemblage
↪  "," MeanPrestige "," MaxPrestige) ]
    ask bands [ file-print ( word "" "," who "," pointAssemblage "," potAssemblage )]
    file-close

    ; save variables to a different file
    set fileNameCSV ( word "results/run_outputVariables_" fileName ".csv" )
    file-open fileNameCSV
    file-print ( "variable,value" )
    file-print ( word "nOfBands," nOfBands )
    file-print ( word "campPopulation," campPopulation )
    file-print ( word "pVisiting," pVisiting )
    file-print ( word "pHunting," pHunting )
    file-print ( word "huntingGroupSize," huntingGroupSize )
    file-print ( word "potBreakageRate," potBreakageRate )
    file-print ( word "pointsBreakageRate," pointsBreakageRate )
    file-print ( word "pNewObject," pNewObject )
    file-print ( word "aggregationFreq," aggregationFreq )
    file-print ( word "aggregationDuration," aggregationDuration )
    file-print ( word "pMigration," pMigration )
    file-print ( word "pNewAlliance," pNewAlliance )
    file-print ( word "maximumAlliesN," maximumAlliesN )
    file-print ( word "learningMethod," learningMethod )
    file-print ( word "transmissionRate," transmissionRate )
    file-print ( word "gradualEnvChange," gradualEnvChange )
    file-print ( word "pEnvChange," pEnvChange )
```

```
    file-print ( word "nTicks," nTicks )
    file-print ( word "randomSeed," randomSeed )
    file-print ( word "uniqueTraits," uniqueTraits )
    file-close

    ; save network links to a different file
    set fileNameCSV ( word "results/run_outputLinks_" fileName ".csv" )
    file-open fileNameCSV
    file-print ( "end1,end2,learning,hunting,visiting,trading" )
```

exportFineTunedData

Description Observer procedure. Writes adjacency tables data to a separate file at 1000 ticks interval if wanted.

```
to exportFineTunedData

  file-open fileHighRes ; open the file already created and write the adjacency tables
↪  info with ticks (one per row)
  file-print ( word ticks ",campVisiting," matrix:to-row-list campVisitingAdjacency )
  file-print ( word ticks ",campTrading," matrix:to-row-list campTradingAdjacency )
  file-print ( word ticks ",campHunting," matrix:to-row-list campHuntingAdjacency )
  file-print ( word ticks ",campLearning," matrix:to-row-list campLearningAdjacency )
  file-print ( word ticks ",campPots," matrix:to-row-list campPotAdjacency )
  file-print ( word ticks ",campPoints," matrix:to-row-list campPointAdjacency )
  file-close

end
```

getJaccardBand

Parameters objectType is either "points" or "pots".

Description Observer procedure. Calculates the Jaccard distance between the traits found in the camps belonging to each band. Essentially this is a measure where 0 is most similar and 1 is most distant for sharing the same types and variants between bands.

```
to-report getJaccardBand [ objectType ]

  ; temporary variable
  let allDist []

  ; create links between bands and calculate distances between each linked band
  ask bands [
    create-tempLinks-with other bands [ set hidden? true] ; this makes sure distances are
↪  not calculated twice
    set allDist lput calculateJaccard objectType allDist ; call the procedure that
↪  actually calculates the distance and add it to the master list
  ]
  ifelse not empty? allDist [
    set allDist ( reduce sentence allDist ) ; remove all square brackets in the master
↪  list (that were added as each band reports 2 distances as one list)
```

```
  ] [
    set allDist [0]
  ]
  report precision mean allDist 3 ; report the mean of all inter-band distances

end
```

getJaccardCamp

Parameters objectType is either “points” or “pots”.

Description Observer procedure. This is the same as the getJaccardBand procedure except the values are calculated between camps.

```
to-report getJaccardCamp [ objectType ]

  ; temporary variable
  let allDist []

  ; create links between bands and calculate distances between each linked band
  ask camps [
    create-tempLinks-with other camps [ set hidden? true] ; this makes sure distances are
↪  not calculated twice
    set allDist lput calculateJaccard objectType allDist ; call the procedure that
↪  actually calculates the distance and add it to the master list
  ]
  ifelse not empty? allDist [
    set allDist ( reduce sentence allDist ) ; remove all square brackets in the master
↪  list (that were added as each band reports 2 distances as one list)
  ] [
    set allDist [0]
  ]
  report precision mean allDist 3 ; report the mean of all inter-band distances

end
```

calculateJaccard

Parameters objectType is either “points” or “pots”.

Description Observer procedure. This function calculates the Jaccard distance between two lists.

```
to-report calculateJaccard [ objectType ]

  ; populate variables with all distinct types of traits from people in that camp or band
  listTraits

  ; temporary variables used below
  let allTraitsA table:make
  let allTraitsB table:make
  let allDist []
```

```
  let dist 0

  ; calculate Jaccard distances between linked bands or camps
  set allTraitsA ifelse-value objectType = "points" [ allPoints ][ allPots ] ; take on
↪  the traits of the band/camp's people
  set allTraitsA remove-duplicates (table:keys allTraitsA)
  ask tempLink-neighbors [ ; asks the linked agents (bands or camps)
    set allTraitsB ifelse-value objectType = "points" [ allPoints ][ allPots ] ; take on
↪  the traits of the band/camp's people
    set allTraitsB remove-duplicates table:keys allTraitsB
    set dist jaccardDist allTraitsA allTraitsB
    set allDist lput dist allDist ; add the dist calculated to the allDist list
  ]

  ask tempLinks [ die ] ; always remove the links after we used them
  report allDist

end
```

adjCompare

Parameters m1 and m2 are matrices.

Return Returns a number between zero and one where one represents identical matrices and zero is no similarity.

Description Observer procedure. This function calculates the absolute differences between two matrices. It is a method to compare networks.

```
to-report adjCompare [m1 m2]
  let result 0
  carefully[
    let nr item 0 matrix:dimensions m1
    let md median reduce sentence reduce sentence matrix:to-row-list m1
    set m1 matrix:map [x -> ifelse-value (x > md) [1][0]] m1
    set md median reduce sentence reduce sentence matrix:to-row-list m2
    set m2 matrix:map [x -> ifelse-value (x > md) [1][0]] m2
    let netDiff matrix:minus m1 m2
    set netDiff matrix:map abs netDiff
    let out 1 - (sum reduce sentence reduce sentence matrix:to-row-list netDiff) / (nr *
↪  (nr - 1))
    set result precision out 3
  ][set result "NA"]
  report result
end
```

adjCorr

Parameters m1 and m2 are matrices

Return Returns a number between -1 and 1 representing the Pearson's R correlation.

Description Observer procedure. This procedure uses the sr extension which is disabled by default and this code is commented out. To enable. Uncomment the sr extension, follow the sr setup instructions, install R on your computer with the igraph and multinet packages, and uncomment this code. This procedure takes two matrices and converts them to networks. It then calculates the Pearson's R correlation between the network edges. It is a method to compare networks.

```
;to-report adjCorr [m1 m2]
;  let result 0
;  carefully[
;    let md median reduce sentence reduce sentence matrix:to-row-list m1
;    set m1 matrix:map [x -> ifelse-value (x > md) [1][0]] m1
;    set md median reduce sentence reduce sentence matrix:to-row-list m2
;    set m2 matrix:map [x -> ifelse-value (x > md) [1][0]] m2
;    sr:set "m1" matrix:to-row-list m1
;    sr:set "m2" matrix:to-row-list m2
;    (sr:run
;      "m1 = as.matrix(do.call(rbind,m1))"
;      "m2 = as.matrix(do.call(rbind,m2))"
;      "rownames(m1) = colnames(m1) = 1:nrow(m1)"
;      "rownames(m2) = colnames(m2) = 1:nrow(m2)"
;      "print(m1)"
;      "g1 = igraph::graph_from_adjacency_matrix(m1, mode = 'undirected', diag = F)"
;      "g2 = igraph::graph_from_adjacency_matrix(m2, mode = 'undirected', diag = F)"
;      "net = multinet::ml_empty('comparison')"
;      "multinet::add_igraph_layer_ml(n = net, g = g1,name = 'm1')"
;      "multinet::add_igraph_layer_ml(n = net, g = g2,name = 'm2')"
;      "comp = multinet::layer_comparison_ml(net, method = 'pearson.degree', mode =
↪  'all')"
;    )
;    set result precision sr:runresult "comp[1,2]" 3
;  ][set result "NA"]
;  report result
;end
```

GUI elements

Name	Type	Value	Properties
nTicks	INPUTBOX	2000.0	entrytype: Number
randomSeed	INPUTBOX	9433.0	entrytype: Number
randomizeSeed	SWITCH	TRUE	
saveResults	SWITCH	TRUE	
pLoss	SLIDER	33	min: 0; max: 100; incr: 1
aggregationFreq	SLIDER	12	min: 6; max: 36; incr: 1
pNewObject	SLIDER	10	min: 0.05; max: 100; incr: 0.05
aggregationDuration	SLIDER	1	min: 0; max: 12; incr: 1
pMigration	SLIDER	0.05	min: 0; max: 10; incr: 0.1
transmissionRate	SLIDER	5	min: 0; max: 100; incr: 1
nOfBands	SLIDER	3	min: 3; max: 6; incr: 1
campPopulation	SLIDER	15	min: 1; max: 100; incr: 1
pHunting	SLIDER	1	min: 0; max: 100; incr: 1
pVisiting	SLIDER	5	min: 0; max: 100; incr: 1

Name	Type	Value	Properties
huntingGroupSize	SLIDER	5	min: 1; max: 100; incr: 1
pNewAlliance	SLIDER	0.5	min: 0; max: 10; incr: 0.05
maximumAlliesN	SLIDER	50	min: 10; max: 150; incr: 1
pLuckyLeap	SLIDER	1	min: 0; max: 10; incr: 0.1
learningMethod	SLIDER	50	min: 0; max: 100; incr: 1
recordPrestige	SWITCH	TRUE	
gradualEnvChange	SWITCH	TRUE	
pEnvChange	SLIDER	0.1	min: 0; max: 20; incr: 0.1
uniqueTraits	SWITCH	TRUE	
visibility	SWITCH	TRUE	
genderedMigration	SWITCH	TRUE	
pNewVariant	SLIDER	6	min: 0; max: 100; incr: 1
potteryPrestige	SWITCH	TRUE	
pTrading	SLIDER	5	min: 0; max: 100; incr: 1
recordPerson	SWITCH	TRUE	
debug	SWITCH	TRUE	
showMetrics	SWITCH	TRUE	
HighResolutionData	SWITCH	TRUE	

References cited

Creanza, N., Kolodny, O., Feldman, M.W., 2017. Greater than the sum of its parts? Modelling population contact and interaction of cultural repertoires. Journal of The Royal Society Interface. 14, 20170171.

Derex, M., Mesoudi, A., 2020. Cumulative Cultural Evolution within Evolving Population Structures. Trends in Cognitive Sciences. 24, 654–667.

Hamilton, M.J., 2021. Collective computation and the emergence of hunter-gatherer small-worlds (preprint). Ecology.

Kolodny, O., Creanza, N., Feldman, M.W., 2015. Evolution in leaps: The punctuated accumulation and loss of cultural innovations. Proceedings of the National Academy of Sciences. 112, E6762–E6769.

Mesoudi, A., 2011. Cultural Evolution: How Darwinian Theory Can Explain Human Culture and Synthesize the Social Sciences. University of Chicago Press.

Migliano, A.B., Battiston, F., Viguier, S., Page, A.E., Dyble, M., Schlaepfer, R., Smith, D., Astete, L., Ngales, M., Gomez-Gardenes, J., Latora, V., Vinicius, L., 2020. Hunter-gatherer multilevel sociality accelerates cumulative cultural evolution. Science Advances. 6, eaax5913.

Powell, A., Shennan, S., Thomas, M.G., 2009. Late Pleistocene Demography and the Appearance of Modern Human Behavior. Science. 324, 1298–1301.

Premo, L.S., Tostevin, G.B., 2016. Cultural Transmission on the Taskscape: Exploring the Effects of Taskscape Visibility on Cultural Diversity. PLOS ONE. 11, e0161766.

Rorabaugh, A., 2015. Modeling Pre-European Contact Coast Salish Seasonal Social Networks and Their Impacts on Unbiased Cultural Transmission. Journal of Artificial Societies and Social Simulation. 18.

Salali, G.D., Chaudhary, N., Bouer, J., Thompson, J., Vinicius, L., Migliano, A.B., 2019. Development of social learning and play in BaYaka hunter-gatherers of Congo. Scientific Reports. 9.

Salali, G.D., Chaudhary, N., Thompson, J., Grace, O.M., van der Burgt, X.M., Dyble, M., Page, A.E., Smith, D., Lewis, J., Mace, R., Vinicius, L., Migliano, A.B., 2016. Knowledge-Sharing Networks in Hunter-Gatherers and the Evolution of Cumulative Culture. Current Biology. 26, 2516–2521.

Yasuoka, H., 2006. Long-Term Foraging Expeditions (Molongo) among the Baka Hunter-Gatherers in the Northwestern Congo Basin, with Special Reference to the “Wild Yam Question.” Human Ecology. 34, 275–296.
