# Supplementary material for: Bridging material culture networks and social networks in archaeology
Source: PLoS One. 2026 Oct 16;21(10):e0358860. doi: 10.1371/journal.pone.0358860 (PMC13674714; doi:10.1371/journal.pone.0358860)
Supplement: Supplementary file 2 [file pone.0358860.s004.pdf]

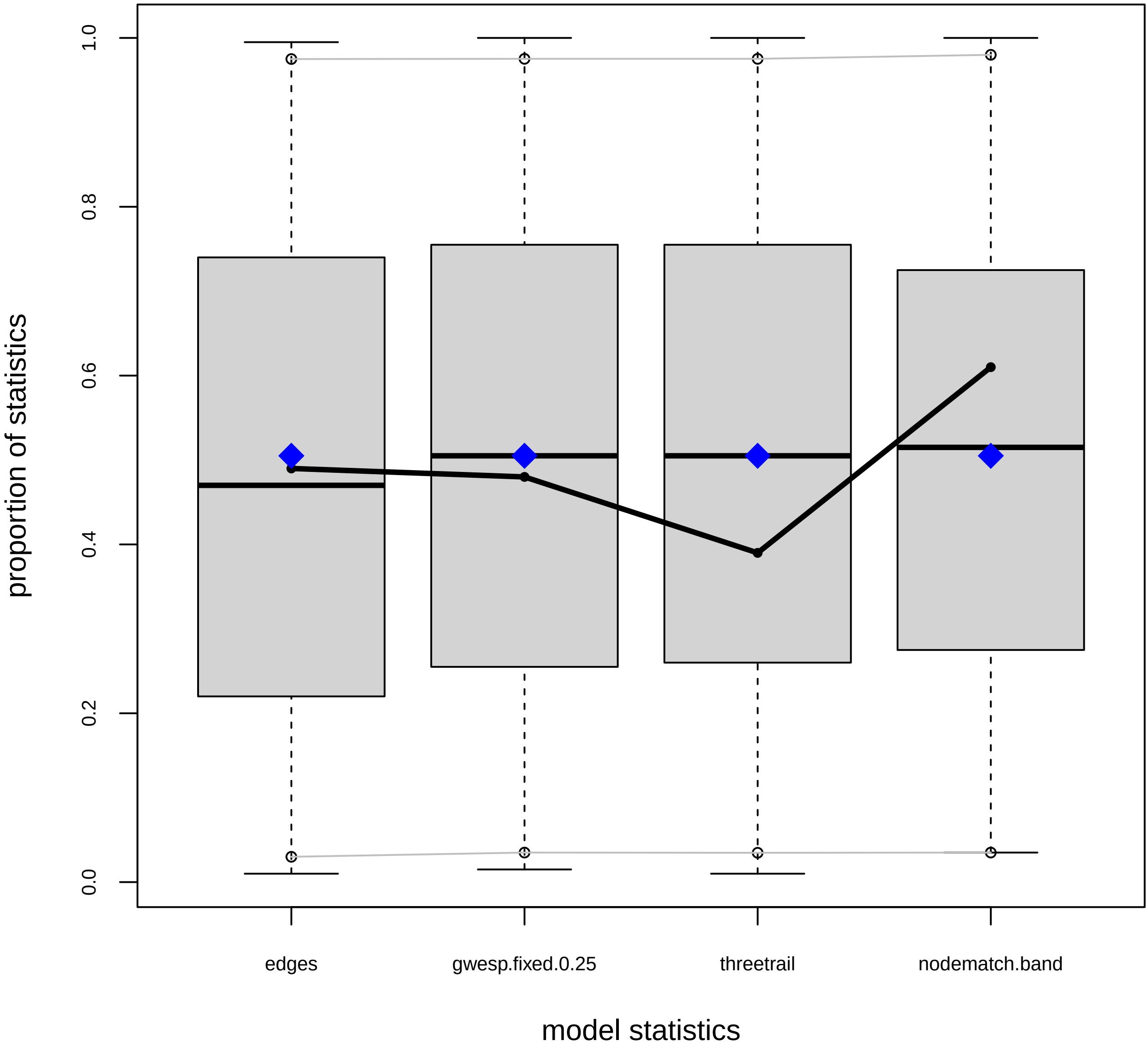

proportion of statistics
0.0
0.2
0.4
0.6
0.8
1.0
edges
gwesp.fixed.0.25
threetrail
nodematch.band
model statistics

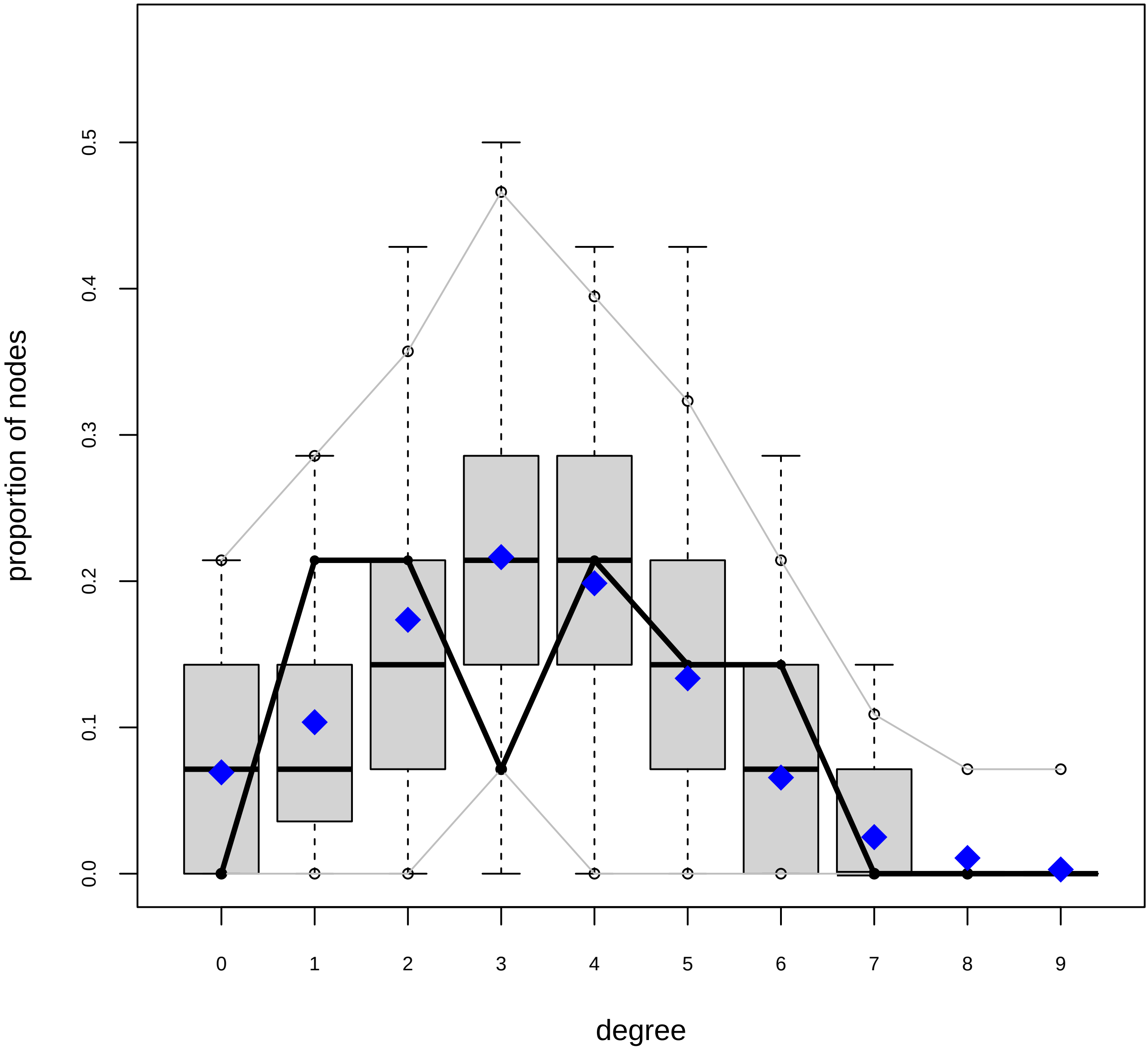

proportion of nodes
0.0
0.1
0.2
0.3
0.4
0.5
0
1
2
3
4
5
6
7
8
9
degree

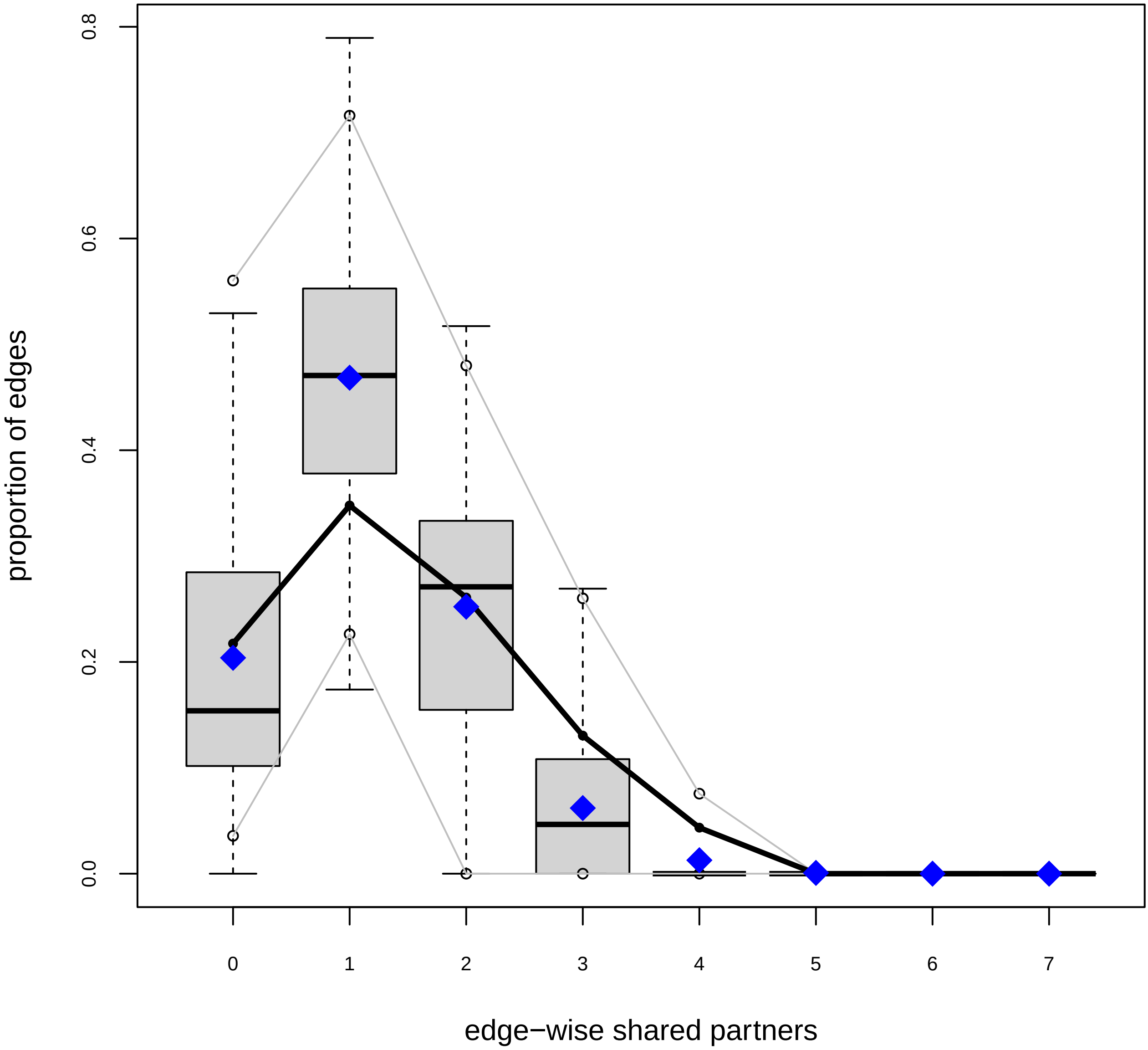

0.0
0.2
0.4
0.6
0.8
proportion of edges
0
1
2
3
4
5
6
7
edge−wise shared partners

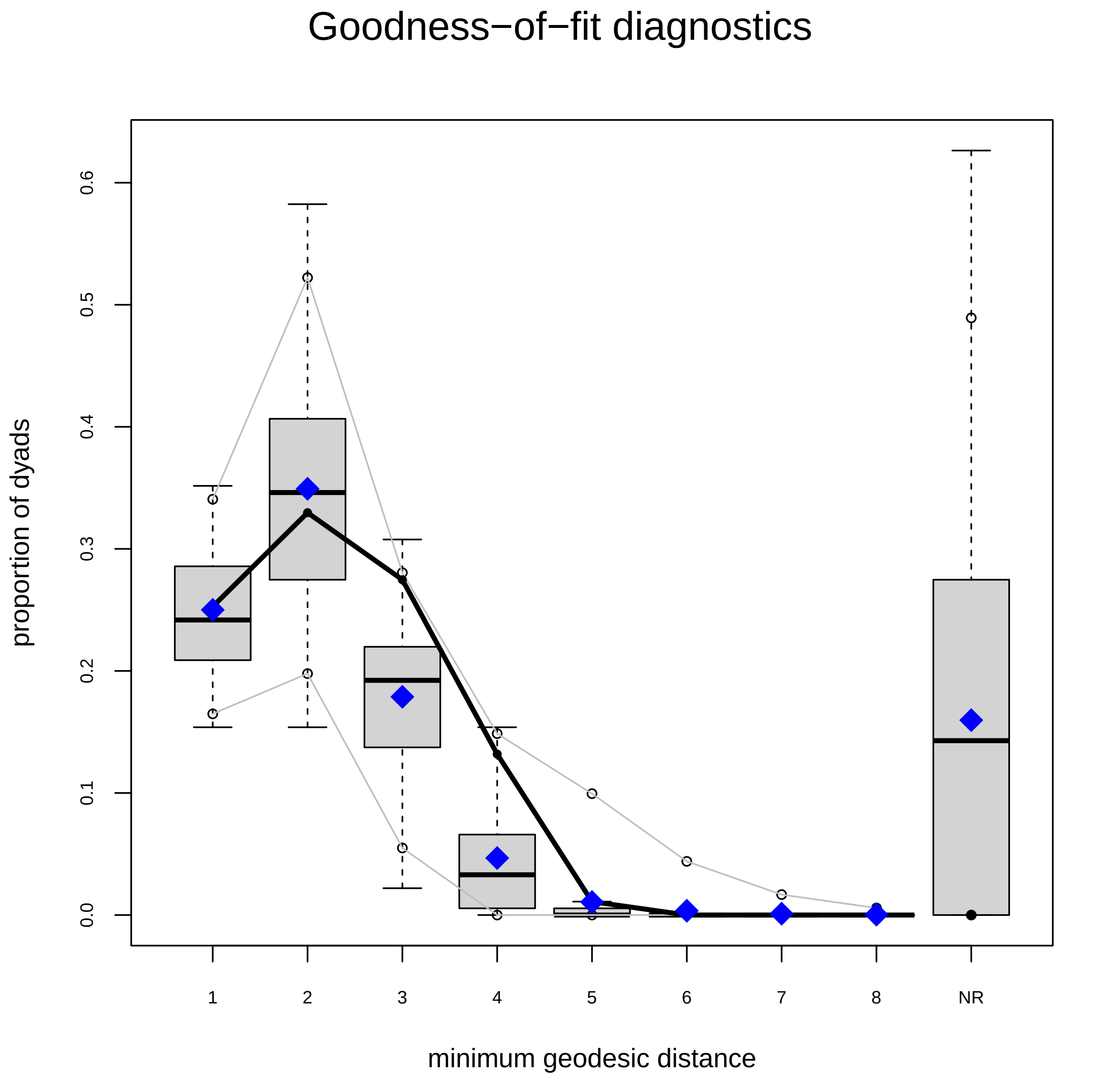
Goodness−of−fit diagnostics
proportion of dyads
minimum geodesic distance
0.0
0.1
0.2
0.3
0.4
0.5
0.6
1
2
3
4
5
6
7
8
NR

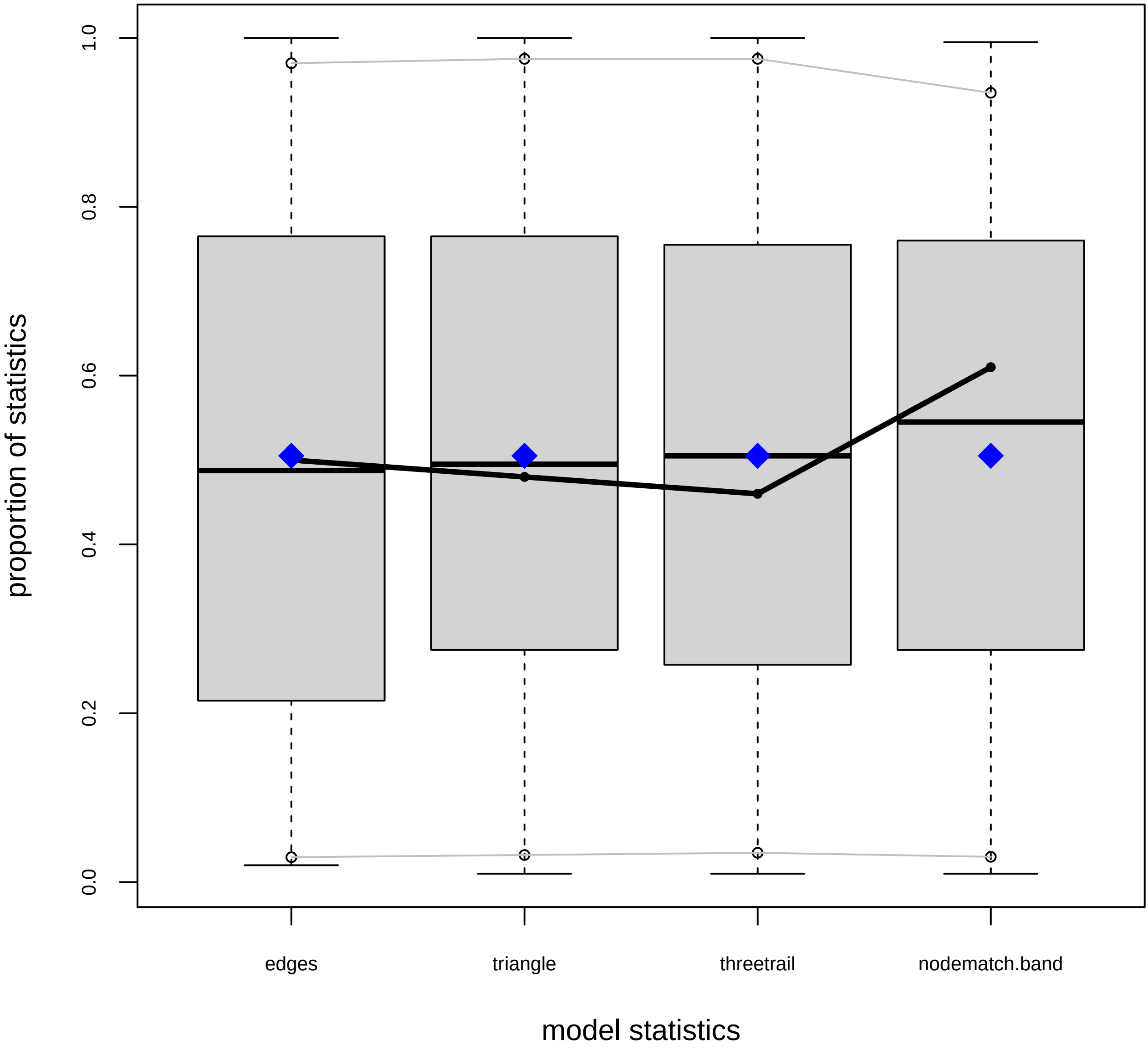

proportion of statistics
0.0
0.2
0.4
0.6
0.8
1.0
edges
triangle
threetrail
nodematch.band
model statistics

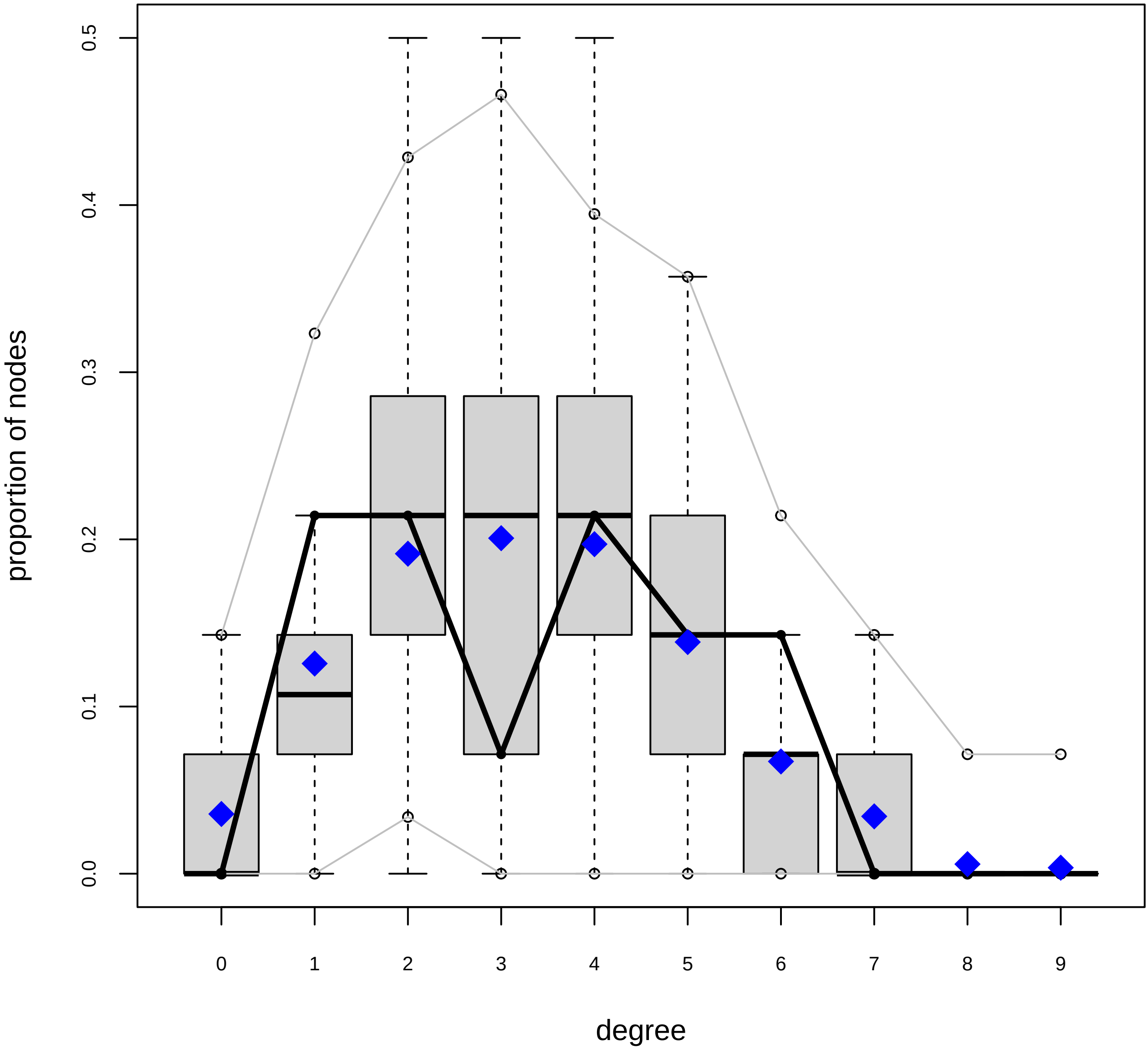

proportion of nodes
0.0
0.1
0.2
0.3
0.4
0.5
0
1
2
3
4
5
6
7
8
9
degree

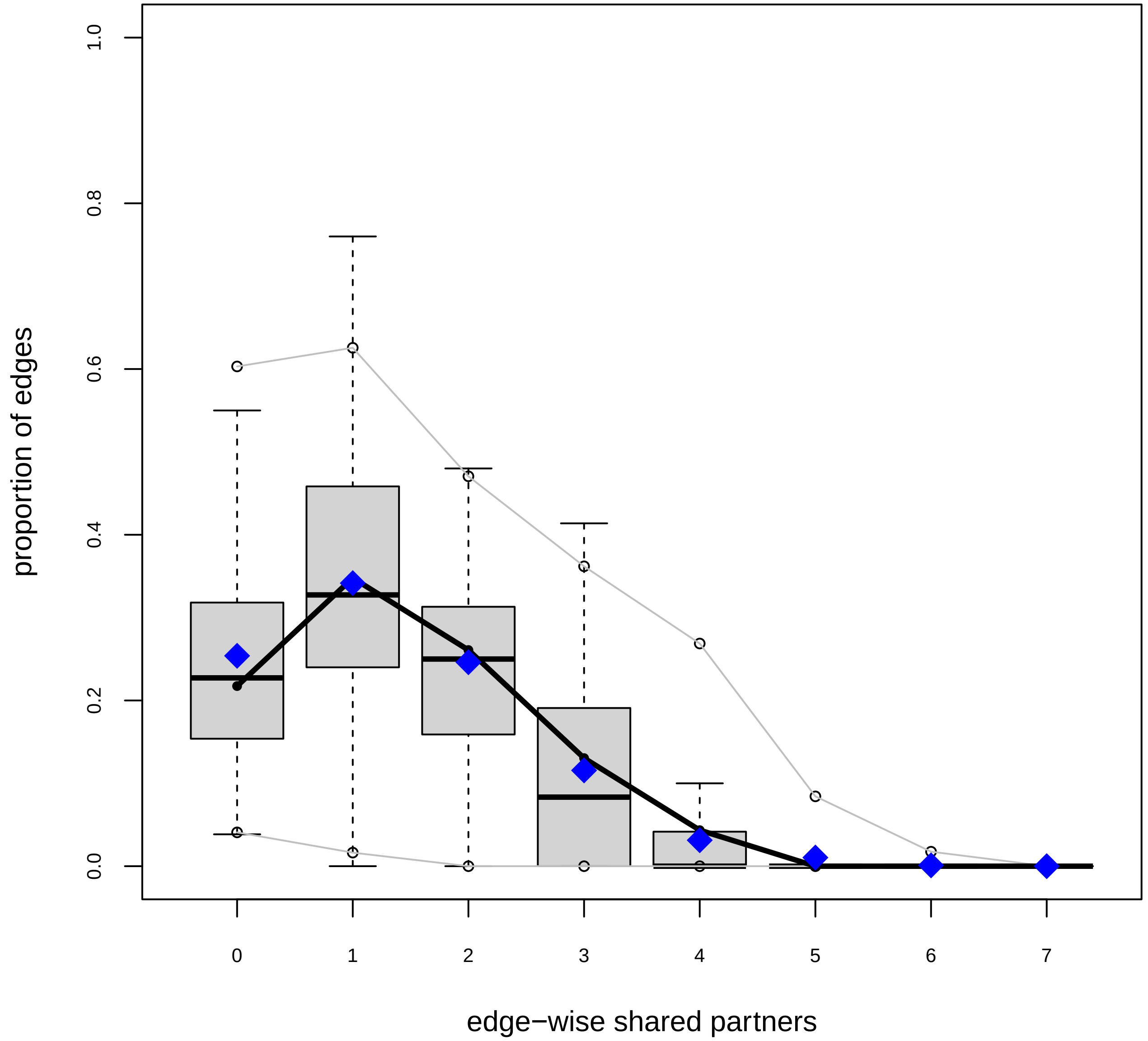

1.0
0.8
0.6
0.4
0.2
0.0
proportion of edges
0
1
2
3
4
5
6
7
edge−wise shared partners

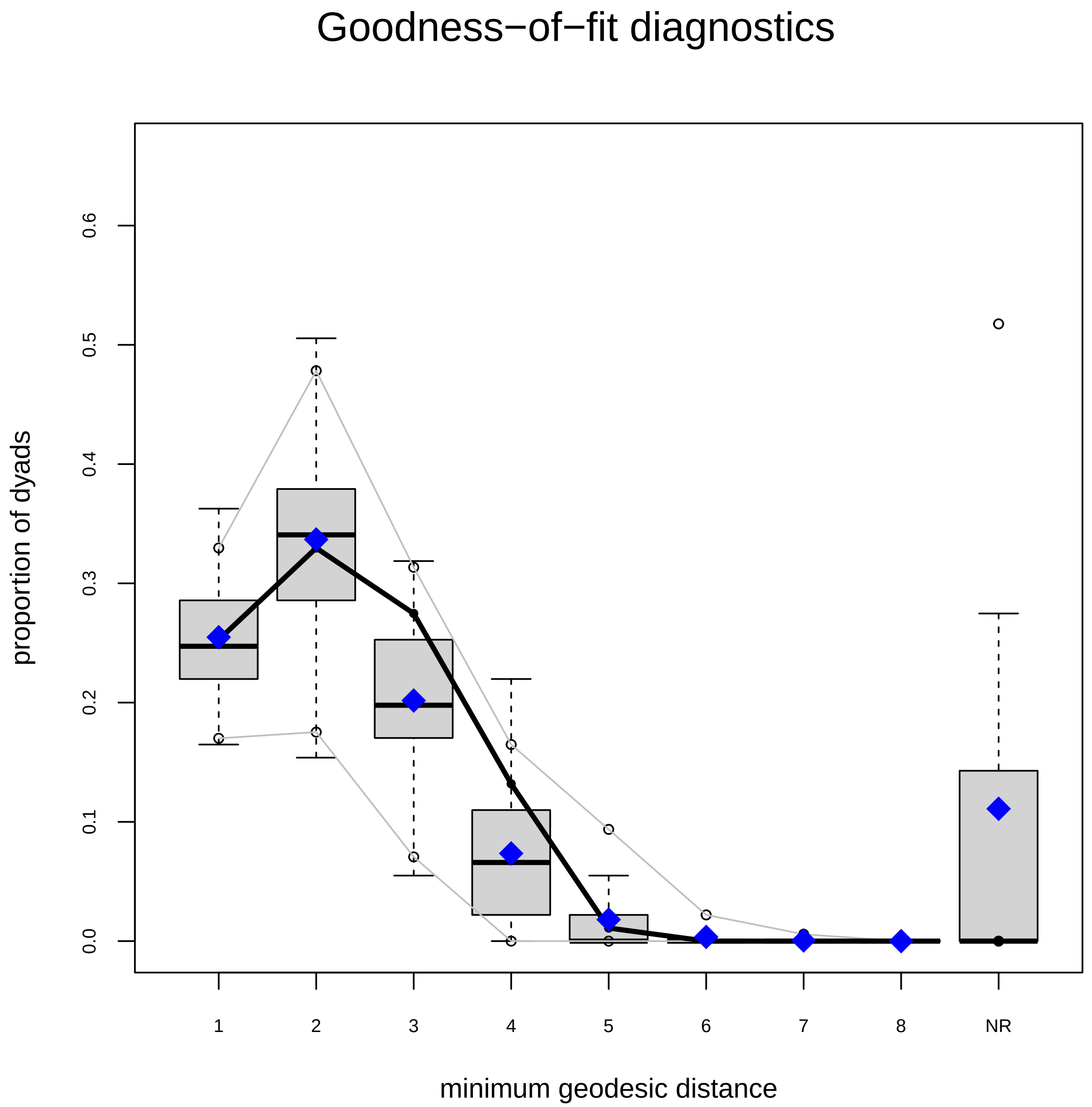

Goodness−of−fit diagnostics
proportion of dyads
minimum geodesic distance
0.0
0.1
0.2
0.3
0.4
0.5
0.6
1
2
3
4
5
6
7
8
NR

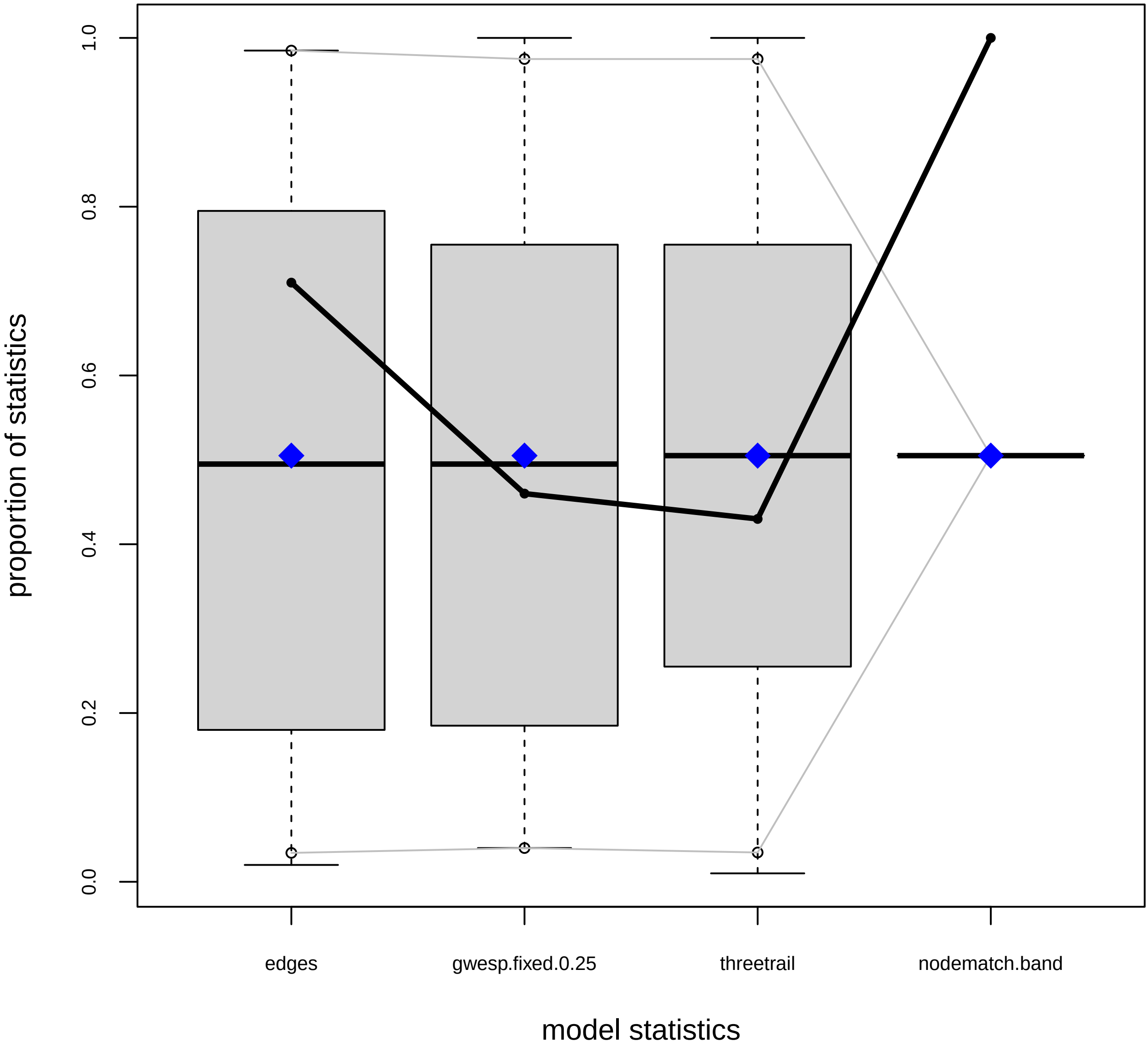

proportion of statistics
0.0
0.2
0.4
0.6
0.8
1.0
edges
gwesp.fixed.0.25
threetrail
nodematch.band
model statistics

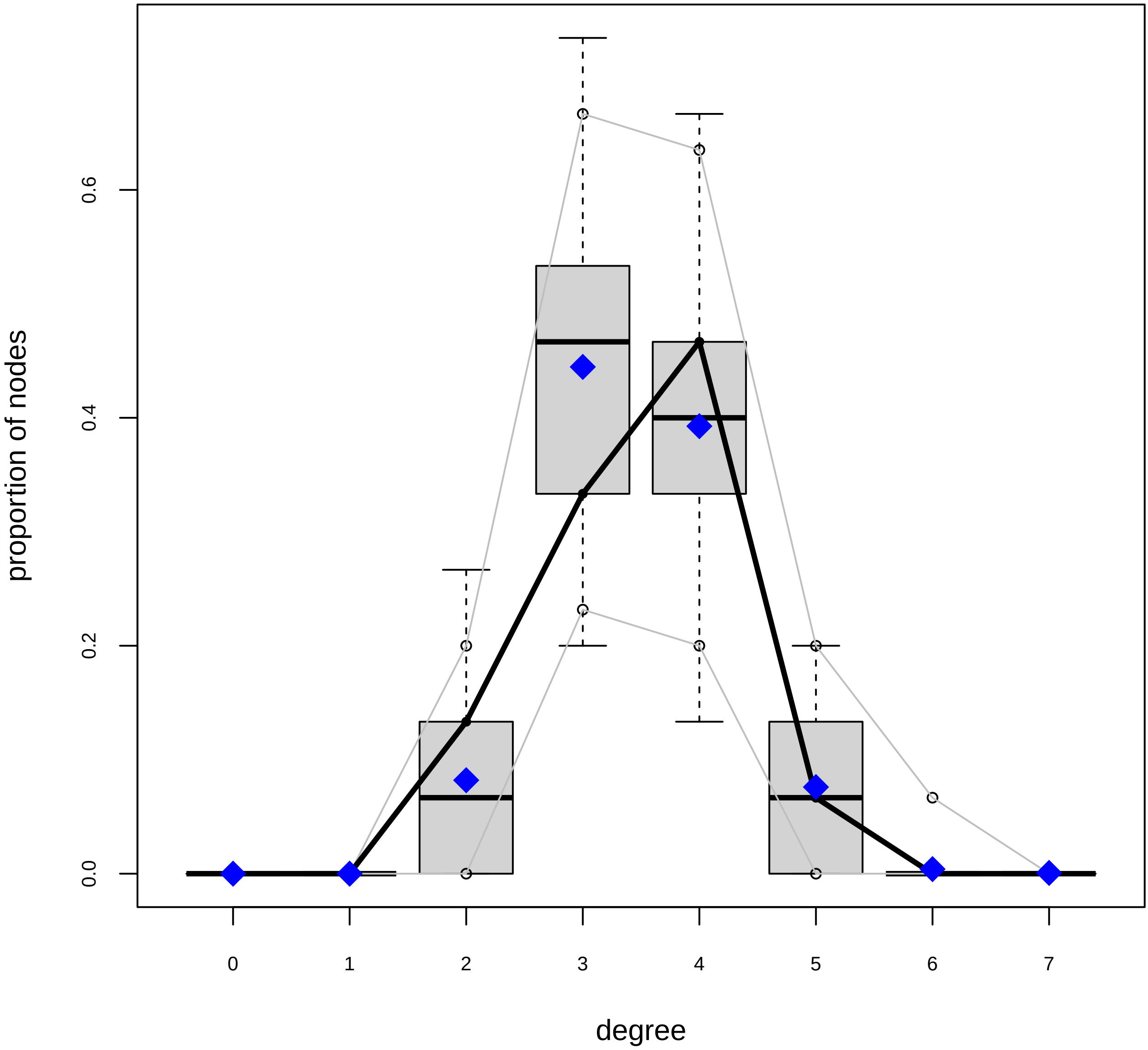

proportion of nodes
0.0
0.2
0.4
0.6
degree
0
1
2
3
4
5
6
7

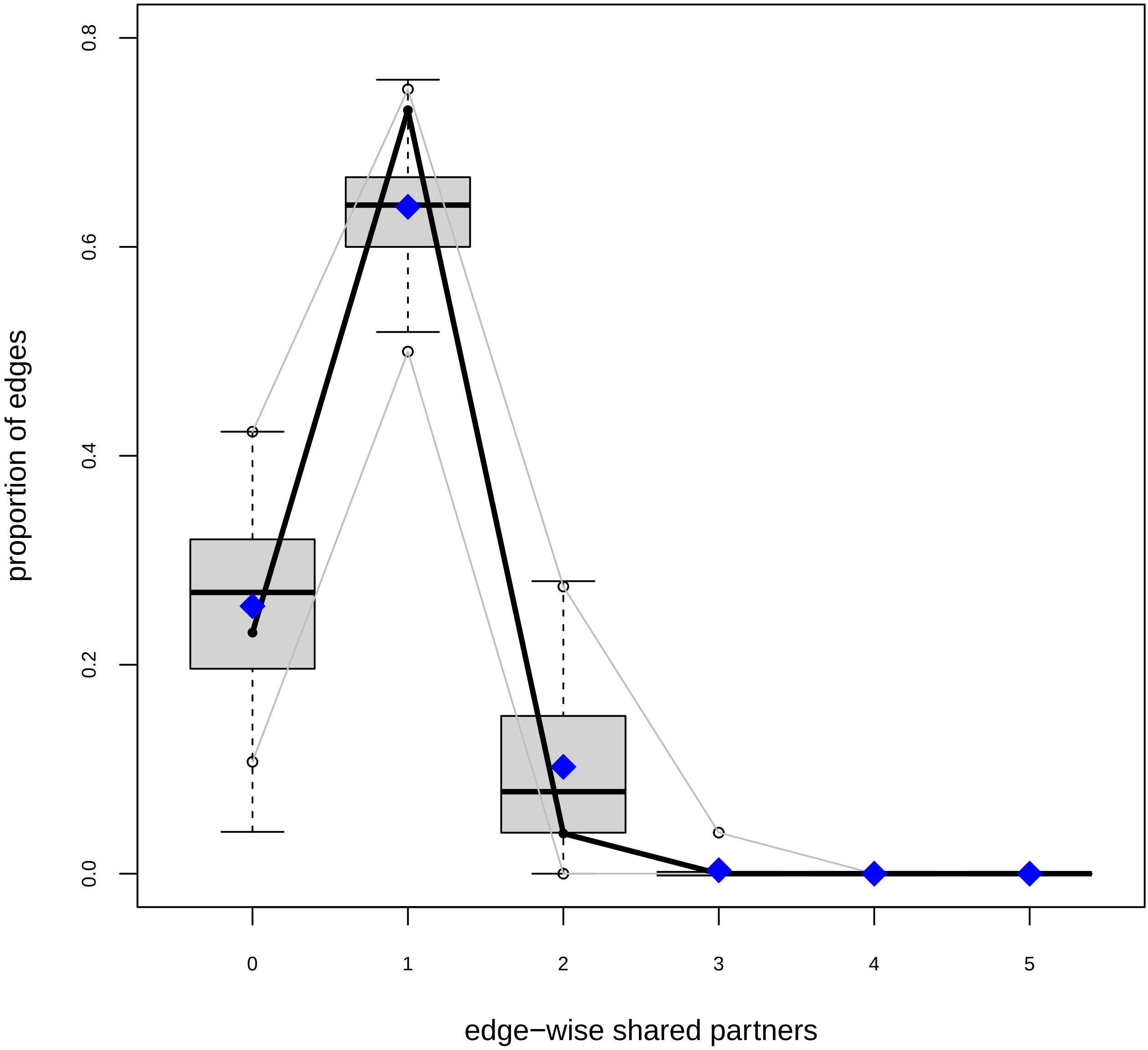

0.0
0.2
0.4
0.6
0.8
proportion of edges
0
1
2
3
4
5
edge−wise shared partners

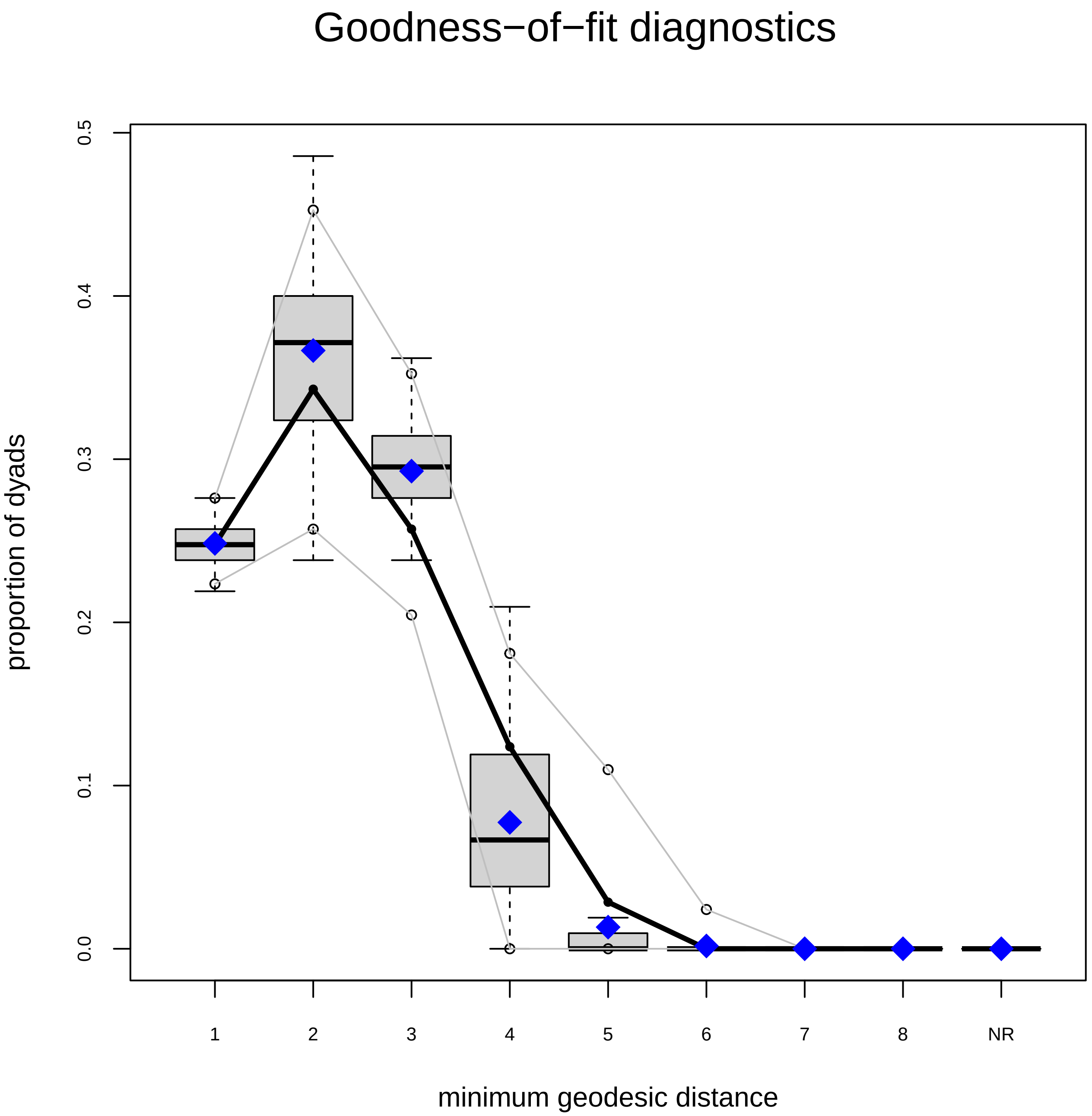

Goodness−of−fit diagnostics
proportion of dyads
minimum geodesic distance
0.0
0.1
0.2
0.3
0.4
0.5
1
2
3
4
5
6
7
8
NR

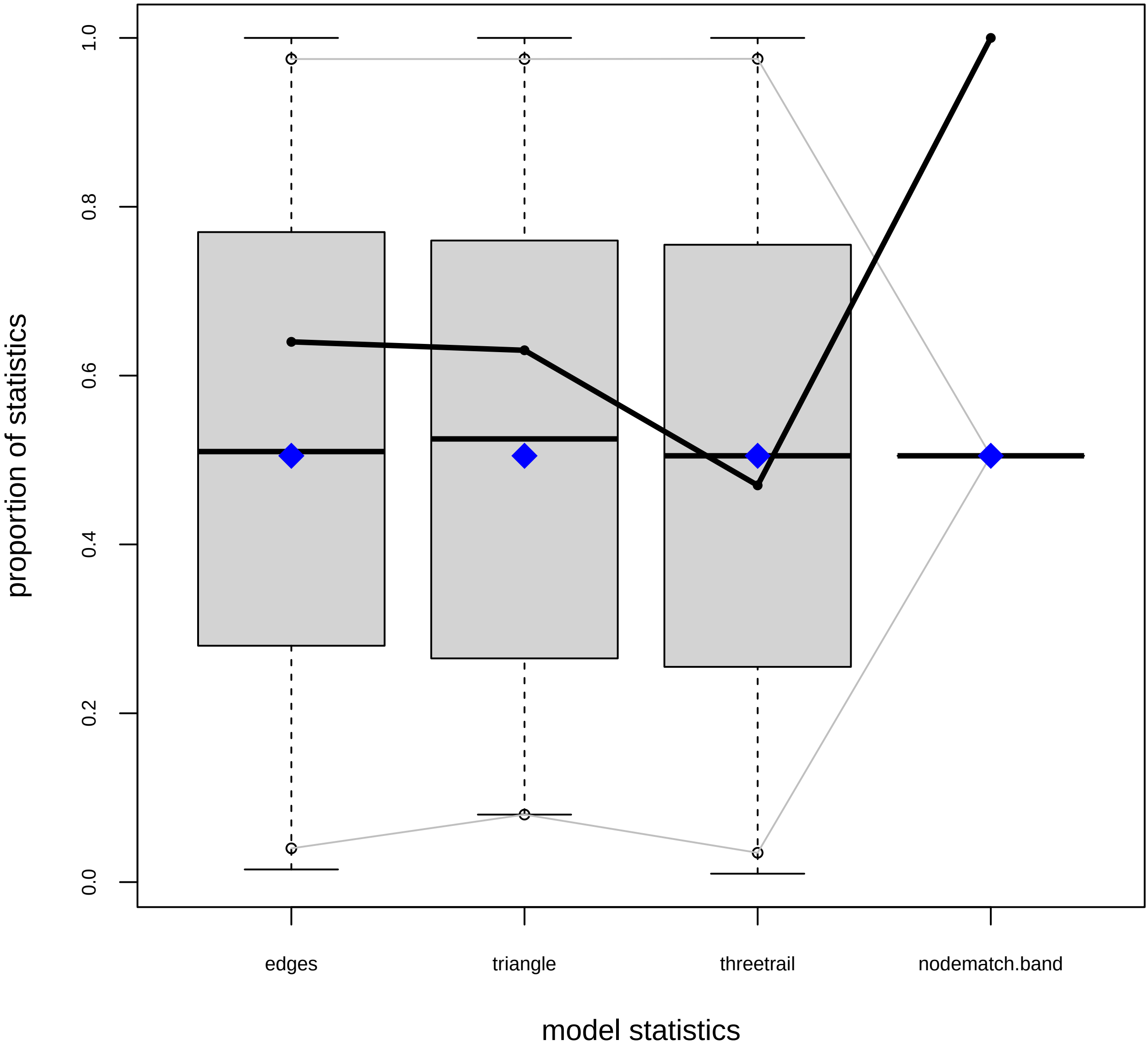

1.0
0.8
0.6
0.4
0.2
0.0
proportion of statistics
edges
triangle
threetrail
nodematch.band
model statistics

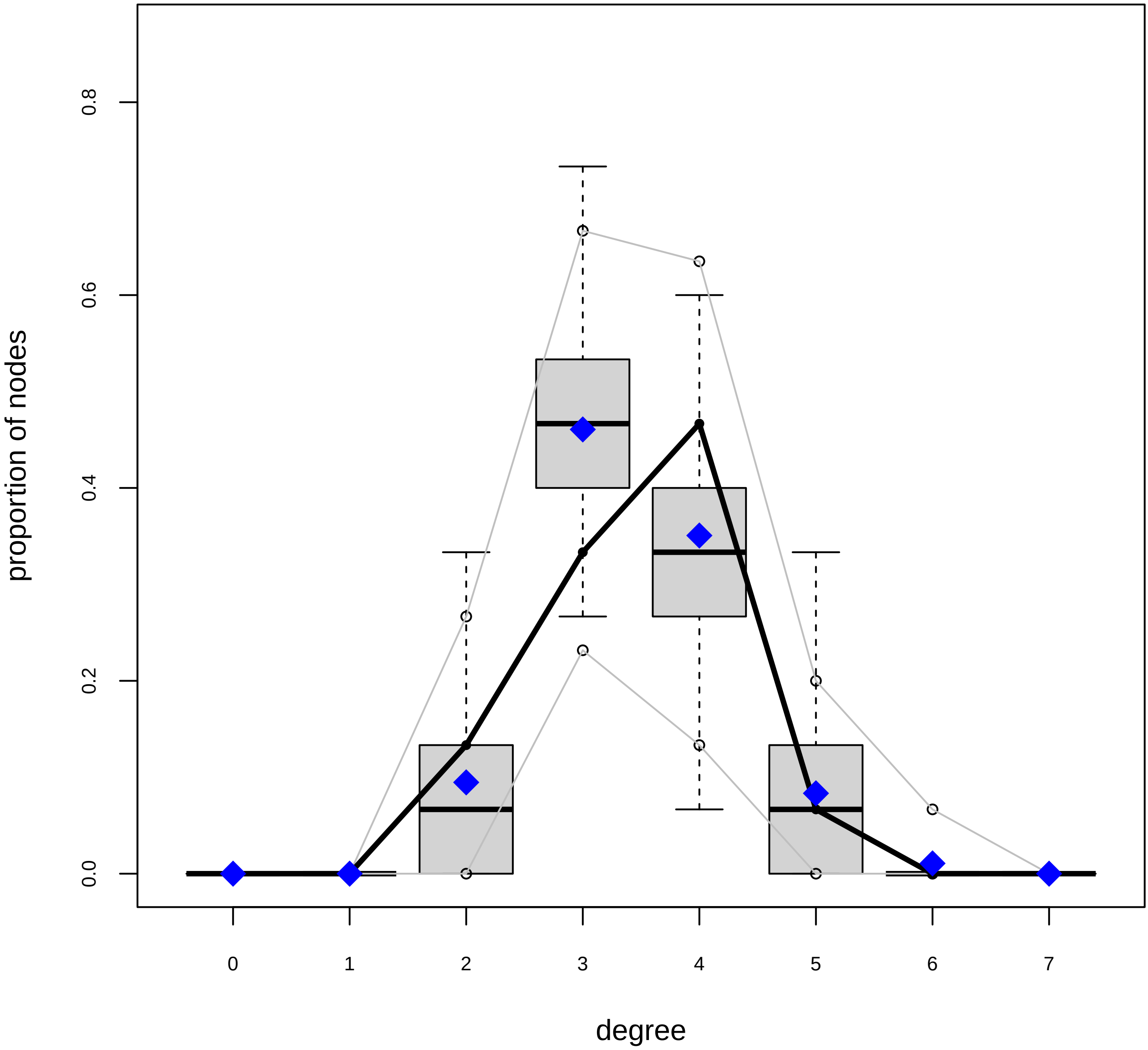

proportion of nodes
0.0
0.2
0.4
0.6
0.8
0
1
2
3
4
5
6
7
degree

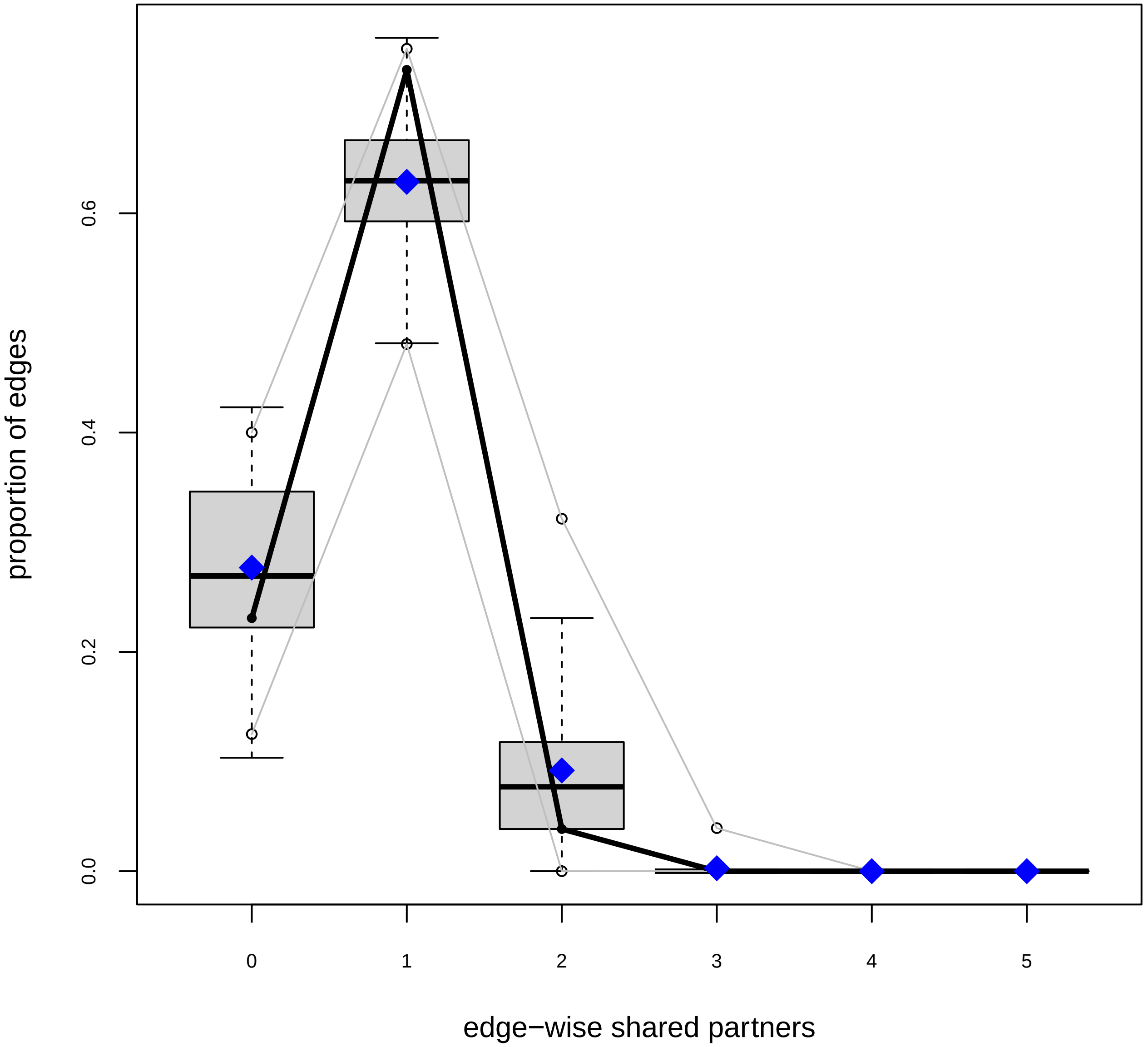

proportion of edges
0.0
0.2
0.4
0.6
0
1
2
3
4
5
edge−wise shared partners

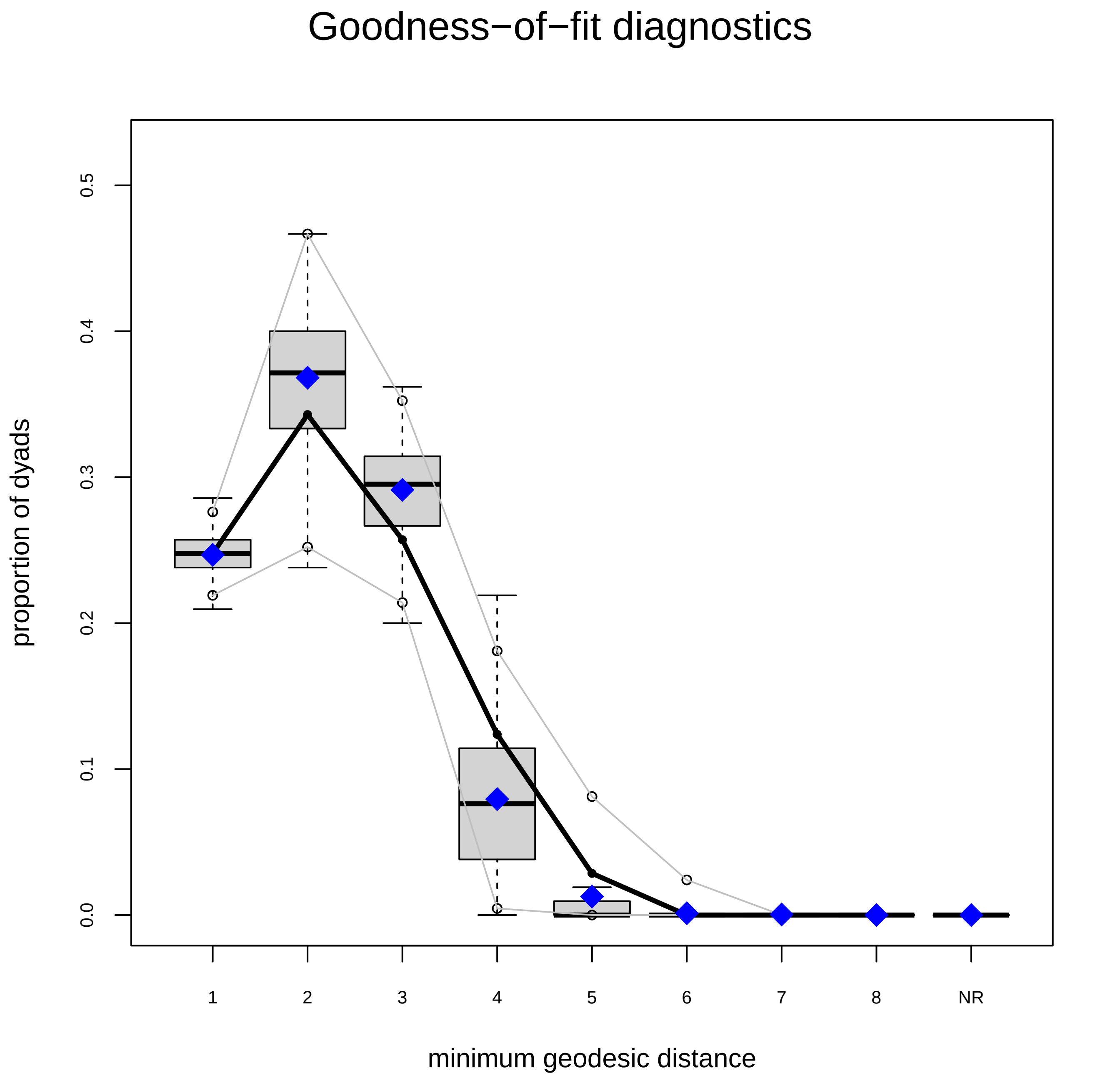
Goodness−of−fit diagnostics
proportion of dyads
minimum geodesic distance
0.0
0.1
0.2
0.3
0.4
0.5
1
2
3
4
5
6
7
8
NR
